# Carbon Emissions From Fires in Eastern Siberian Larch Forests

**DOI:** 10.1111/gcb.70247

**Published:** 2025-05-23

**Authors:** Clement J. F. Delcourt, Brendan M. Rogers, Linar Akhmetzyanov, Brian Izbicki, Rebecca C. Scholten, Tatiana A. Shestakova, Dave van Wees, Michelle C. Mack, Ute Sass‐Klaassen, Sander Veraverbeke

**Affiliations:** ^1^ Faculty of Science Vrije Universiteit Amsterdam Amsterdam the Netherlands; ^2^ Laboratoire des Sciences du Climat et de l'Environnement, LSCE/IPSL, CEA‐CNRS‐UVSQ Université Paris‐Saclay Gif‐sur‐Yvette France; ^3^ Woodwell Climate Research Center Falmouth Massachusetts USA; ^4^ Forest Ecology and Forest Management Wageningen University and Research Wageningen the Netherlands; ^5^ Forest and Nature Management Van Hall Larenstein University of Applied Sciences Velp the Netherlands; ^6^ Center for Ecosystem Science and Society and Department of Biological Sciences Northern Arizona University Flagstaff Arizona USA; ^7^ Department of Earth System Science University of California, Irvine Irvine California USA; ^8^ Department of Agricultural and Forest Sciences and Engineering University of Lleida Lleida Spain; ^9^ Joint Research Unit CTFC–AGROTECNIO–CERCA Lleida Spain; ^10^ BeZero Carbon Ltd London UK; ^11^ School of Environmental Sciences University of East Anglia Norwich UK

**Keywords:** boreal forest, carbon combustion, climate change, eastern Siberia, fire severity, larch forests, *Larix cajanderi*

## Abstract

Siberian boreal forests have experienced increases in fire extent and intensity in recent years, which may threaten their role as carbon (C) sinks. Larch forests (*Larix* spp.) cover approximately 2.6 million km^2^ across Siberia, yet little is known about the magnitude and drivers of carbon combustion in these ecosystems. To address the paucity of field‐based estimates of fuel load and consumption in Siberian larch forests, we sampled 41 burned plots, one to two years after fire, in Cajander larch (*Larix cajanderi*) forests in the Republic of Sakha (Yakutia), Russia. We estimated pre‐fire carbon stocks and combustion with the objective of identifying the main drivers of carbon emissions. Pre‐fire aboveground (trees and woody debris) and belowground carbon stocks at our study plots were 3.12 ± 1.26 kg C m^−2^ (mean ± standard deviation) and 3.50 ± 0.93 kg C m^−2^. We found that combustion averaged 3.20 ± 0.75 kg C m^−2^, of which 78% (2.49 ± 0.56 kg C m^−2^) stemmed from organic soil layers. These results suggest that severe fires in Cajander larch forests can result in combustion rates comparable to those observed in North American boreal forests and exceeding those previously reported for other forest types and burning conditions in Siberia. Carbon combustion was driven by both fire weather conditions and landscape variables, with pre‐fire organic soil depth being the strongest predictor across our plots. Our study highlights the need to better account for Siberian larch forest fires and their impact on the carbon balance, especially given the expected climate‐induced increase in fire extent and severity in this region.

## Graphical abstract

The present study provides new insights on combustion processes from Siberian larch forest fires. We estimated combustion from vegetation and organic soils at 41 burned plots and spatially extrapolated our plot‐level estimates to the areas of two large fire events. Our combustion rates were comparable to those observed in boreal North America and exceeded those previously reported for other forest types and burning conditions in Siberia. We found that carbon combustion was driven by both fire weather conditions and landscape variables. Our results highlight the need to better account for the impact of these fires on the regional carbon balance.

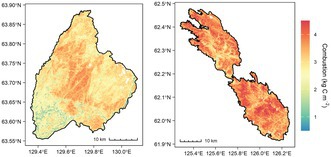

## 1 Introduction

In recent decades, warming and drying in high‐latitude regions has led to intensifying fire regimes in boreal forests, with large increases in extent and severity in North American and Siberian boreal forests (Flannigan et al. 2009; Hanes et al. 2019; Jain et al. 2024; Jones et al. 2022, 2024; Li et al. 2024; Ponomarev et al. 2016, 2021; Soja et al. 2007; Young et al. 2017; Zheng et al. 2021, 2023). The recent years of 2019, 2020, and 2021 experienced the largest burned areas in eastern Siberia since at least 2001 (Scholten et al. 2022), the beginning of the consistent satellite record from the Moderate Resolution Imaging Spectroradiometer (Giglio et al. 2018). In these regions, fires are a natural disturbance that have an important influence on the carbon (C) balance of ecosystems (Stocks et al. 2001). Boreal forests have been considered long‐term C sinks (Goodale et al. 2002; Gurney et al. 2002; Pan et al. 2011), storing around one‐third of the current terrestrial C in the world (Pan et al. 2024). Most of the C is stored belowground in the form of organic soils, as a result of cold temperatures, saturated soil conditions, and poorly decomposable plant litter that slow decomposition rates (Hobbie et al. 2000). Boreal fires emit large amounts of carbonaceous greenhouse gases to the atmosphere by both direct fire emissions from the combustion of vegetation and organic soils, and by longer‐term emissions from soil respiration after fire (Eckdahl et al. 2024; Harden et al. 2000; Kasischke et al. 2005; Kelly et al. 2024; Ueyama et al. 2019). In permafrost regions, fires can accelerate permafrost thaw and degradation, which in turn can enhance post‐fire soil emissions through changes in soil thermal and moisture conditions that influence decomposition processes (Genet et al. 2013; Gibson et al. 2018; Köster et al. 2018; O'Donnell et al. 2011; Schuur et al. 2008). After fire, as the ecosystem recovers, C is sequestered and C stocks are partially or completely replenished over a period of approximately 50 to 200 years, depending on boreal forest ecosystem types (Johnstone et al. 2010; Kharuk et al. 2021). Although regional warming has enhanced boreal forest productivity over the past two decades, fire emissions have significantly offset the resulting increase in CO_2_ uptake (Virkkala et al. 2025). Increased disturbances, including fires, have been associated with a 42% decline in the C sink of Asian Russian boreal forests from 1990 to 2019 (Pan et al. 2024). The climate‐driven increase in fire frequency and intensity has the potential to transition some boreal forest regions from a C sink to a source (Dieleman et al. 2020; Fan et al. 2022; Walker et al. 2019; Wang et al. 2021). To better understand the impacts of changing fire regimes on C dynamics, it is critical to accurately predict C emissions over space and time.

A common approach for estimating C emissions from fires is to calculate the product of burned area, fuel loads, combustion efficiency, and C concentrations (Seiler and Crutzen 1980). Most of the uncertainty in fire emissions arises from the high spatial variability of fuel loads, interactions between fuel structure and fire behavior, and the influence of fuel conditions at the time of fire on fuel consumption (i.e., the amount of C emitted per unit area burned) (French et al. 2011). Field measurements from various forest and vegetation types, climate zones, and under different burning conditions are essential to understand the magnitude and drivers of C combustion. Significant progress has been made to quantify fuel loads and consumption in Alaskan and Canadian boreal forests over recent years (Boby et al. 2010; de Groot et al. 2009; Dieleman et al. 2020; Hoy et al. 2016; Rogers et al. 2014; Turetsky et al. 2011; Walker, Rogers, et al. 2018), with more than 500 field estimates currently available across six ecoregions (Walker, Baltzer, et al. 2020), capturing broad gradients in forest composition and structure, pre‐fire ecosystem C storage, drainage conditions, and fire weather from 18 fires that burned between 2004 and 2015. The increasing volume of field observations has enabled the development of numerous emissions models in boreal North America, using statistical techniques to predict combustion based on field estimates, satellite observations, and other geospatial data (Dieleman et al. 2020; Potter et al. 2023; Rogers et al. 2014; Veraverbeke et al. 2015, 2017; Walker, Rogers, et al. 2018). Field observations are also used for the calibration and validation of regional and global process‐based models, such as the Global Fire Emission Database (GFED) (van der Werf et al. 2017). Eurasian boreal forests are undersampled and, as a result, significantly underrepresented in field databases used to constrain biogeochemical models. For example, among 58 site‐level estimates from boreal forests included in the fuel consumption database used in GFED4s (“s”, including small fires), only three were from Eurasian fires (van Leeuwen et al. 2014). While more than 70% of the total Arctic‐boreal burned area is in Eurasia, the number of field measurements of C combustion is an order of magnitude lower than in North America (Veraverbeke et al. 2021). Additionally, large areas of boreal Eurasia remain poorly studied, with currently available field data limited to northern Europe and central Siberia. This lack of representative sampling limits our understanding of fire process variability across the boreal region and makes estimates of Eurasian fire emissions much more uncertain.

Quantifying and understanding C combustion in Eurasian boreal forests is critical to determine the regional land‐atmosphere C balance, as remote sensing studies show that fire regimes differ between the North American and Eurasian continents (de Groot et al. 2013; Rogers et al. 2015; Scholten et al. 2024), and therefore knowledge from boreal North America may not directly translate to Eurasia. A central component of our understanding of fire regime differences is the disparity in dominant overstory species traits (Rogers et al. 2015). Fire embracers, such as black spruce ( *Picea mariana* Mill.) and jack pine ( *Pinus banksiana* Lamb.), prevail in boreal North America and promote stand‐replacing crown fires that spread rapidly through the overstory canopy. Crown fires are the most intense type of fire and influence surface energy budgets and forest structure for decades (Amiro et al. 2006; O'Halloran et al. 2012; Potter et al. 2020; Randerson et al. 2006; Rogers et al. 2013). Potter et al. (2023) estimated that combustion in northwestern North American boreal forests averaged 3.13 kg C m^−2^ during the 2001–2019 period, of which 90% originated from belowground C pools as a result of deep burning into organic soils. In contrast, fire resisters, particularly larch species ( *Larix sibirica* Ledeb., *Larix gmelinii* Rupr., and *Larix cajanderi* Mayr.) and Scots pine ( *Pinus sylvestris* L.), dominate in boreal Eurasia, resulting in a higher fraction of low‐intensity surface fires that spread through understory vegetation and forest floor litter. In C‐rich soils, sustained surface fires can turn into ground fires that consume the organic matter beneath the surface litter (Kharuk et al. 2021). Most field‐based estimates available for Eurasia are limited to surface fires in the pine forests of central Siberia, where fuel consumption ranged between 0.82 and 1.69 kg C m^−2^ (Ivanova et al. 2011, 2020; Kukavskaya et al. 2013, 2017; McRae et al. 2006). Many of these estimates were derived from experimental fires, which may not be representative of fire weather conditions that occur during wildfires. In particular, deeper belowground combustion might be underestimated as these fires typically exhibit lower energy release. Siberian larch forests in permafrost terrain have experienced large areas of stand‐replacing fires in recent decades (Krylov et al. 2014), yet in situ fuel consumption measurements remain scarce and are often not represented in regional emission models (Ponomarev et al. 2021). A recent study conducted in larch forests of northeastern Siberia reported combustion rates similar to those of North American boreal fires (mean = 3.36 kg C m^−2^), suggesting that emissions from this region may be higher than previously thought (Webb et al. 2024).

Climate influences the type, arrangement, and amount of fuel that accumulates over long timescales (Abatzoglou et al. 2018; Bowman et al. 2009). In boreal forests, most of the C released during wildfires originates from surface and ground fuels (Kasischke et al. 1995). Fuel loading is also related to stand age, which represents the time since the last stand‐replacing disturbance (Dieleman et al. 2020; Walker, Rogers, et al. 2018). Landscape‐level variations in drainage conditions not only control the rate of soil C accumulation but also influence combustion efficiency (Walker, Baltzer, et al. 2018). For instance, Scots pine forests, typically located at drier landscape positions, are more prone to fires than Siberian dark‐coniferous forests ( *Pinus sibirica* , *Abies sibirica*, *Picea obovata*) found in poorly drained areas (Kharuk et al. 2021). However, these well‐drained sites have relatively shallow organic soils, which limits the extent of combustion (Kukavskaya et al. 2023). Fuel consumption is also influenced by topography, permafrost conditions, plant species composition, and the timing of burning during the fire season (Kane et al. 2007; Kasischke and Hoy 2012; Turetsky et al. 2011). At shorter timescales, weather patterns control ignition, fuel availability (i.e., the portion of total fuel load dry enough to burn), as well as fire behavior (Pyne 1984). Recent extreme fire events in Siberia have been linked to severe heatwaves and prolonged droughts that enhanced fire‐prone conditions (Scholten et al. 2022; Zheng et al. 2021). Walker, Rogers, et al. (2020) showed that C combustion in northwestern North American boreal forests was more strongly controlled by fine‐scale drainage conditions, overstory species composition, and fuel accumulation rates than by fire weather conditions. To our knowledge, the factors controlling fuel consumption have not yet been assessed in Siberian larch forests.

The present study builds on a field campaign conducted in 2019 in two fire scars in northeast Siberia, where 41 burned plots were sampled to assess the effects of fire on larch forests. In a previous study (Delcourt et al. 2021), field data were used to assess the potential of a spectral index, the differenced Normalized Burn Ratio (dNBR), as a proxy for fire severity in these ecosystems. Delcourt et al. (2021) evaluated the relationships between the dNBR and field measurements of fire severity, such as the geometrically structured Composite Burn Index (GeoCBI) (De Santis and Chuvieco 2009) and the depth of burn into the soil organic layers. Here, the same plot network was used to quantify C losses from both surface and stand‐replacing fires and to investigate the impact of various landscape and fire weather drivers on combustion rates. We used a multiple regression model to spatially extrapolate our estimates of combustion to the entire fire scar areas, and a Monte Carlo framework to assess uncertainty. This study aims to fill knowledge and data gaps associated with Siberian larch forests by contributing valuable insights to our understanding of fire and C dynamics in these ecosystems.

## 2 Methods

### 2.1 Study Area and Plot Selection

**FIGURE 1:**
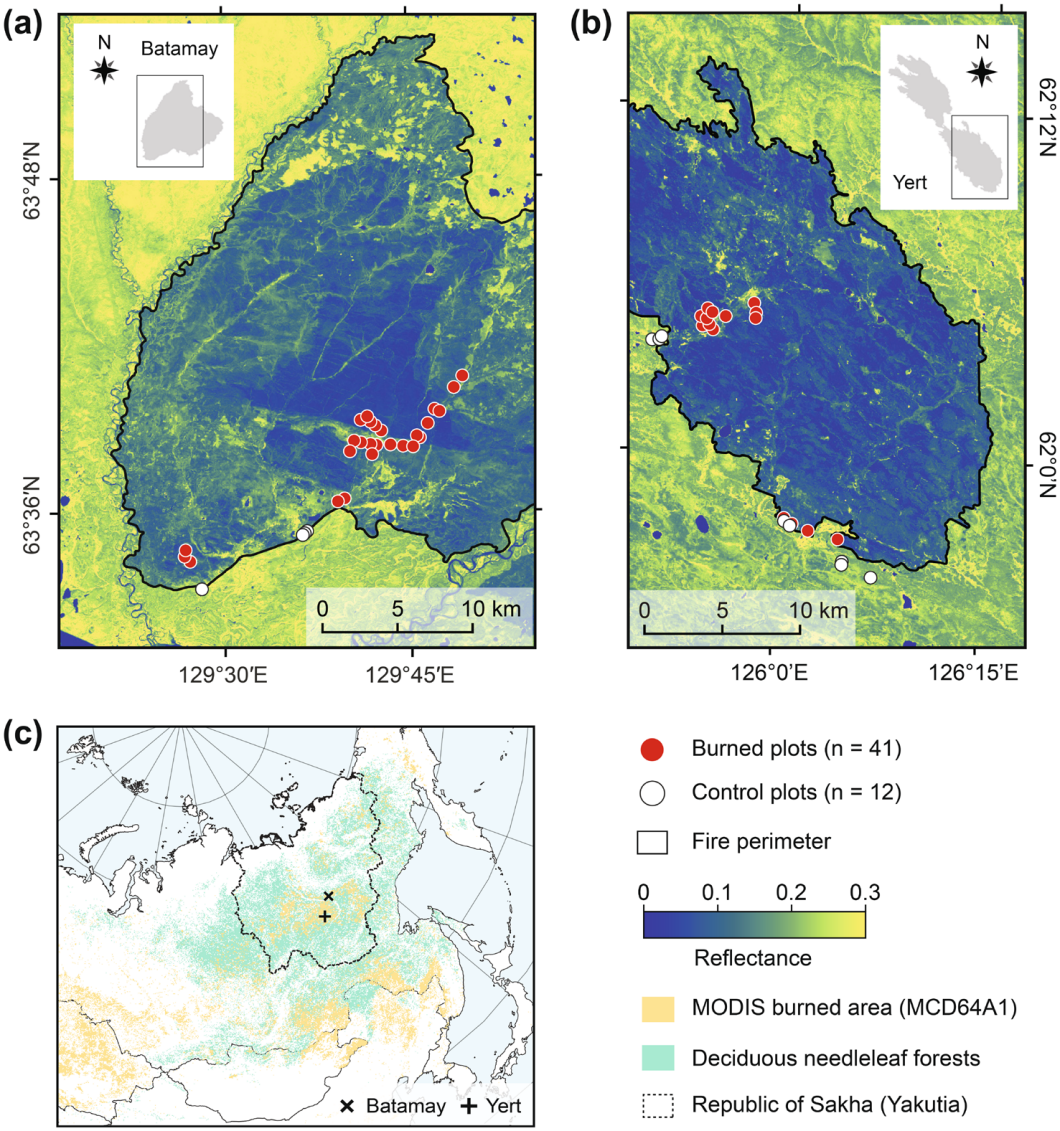
Study areas in the Republic of Sakha (Yakutia), Russia. (a) Batamay fire scar, (b) Yert fire scar, (c) study domain within the larch forests of northeastern Siberia. The background images in panels (a) and (b) are surface reflectance from Sentinel‐2A Multispectral Instrument (MSI) band 8A (855–875 nm, VNIR) post‐fire images (tile ID: T52VER, 13 August 2017; tile ID: T51VXK, 14 August 2018). The area of the entire fire scar is shown in grey in each inset in panels (a) and (b). Fires in panel (c) were retrieved for 2001–2022 using the Terra and Aqua combined MCD64A1 collection 6.1 burned area data product (Giglio et al. 2018). Deciduous needleleaf forest cover is from the land cover map for Northern Eurasia for the Year 2000 (Bartalev et al. 2003). Map lines delineate study areas and do not necessarily depict accepted national boundaries.

In the summer of 2019, we investigated two fire events that burned approximately 800 km^2^ during 25 June to 16 August 2017, and 910 km^2^ during 1 July to 21 July 2018 near Batamay (63.53° N, 129.42° E) and Yert (62.02° N, 125.79° E) towns, Russia (Figure 1). We sampled forest stands dominated by Cajander larch (*L. cajanderi*) with an understory of shrubs (e.g., *Alnus* spp., *Rosa acicularis* , *Salix* spp., *Vaccinium vitis‐idaea* ) and mosses ( *Ceratodon purpureus* , *Aulacomnium palustre* ). According to the permafrost‐landscape classification by Fedorov et al. (2018), this forest type covered approximately 60% of the area of each of the Batamay and Yert burn scars, and accounts for 15% of the land area in the Republic of Sakha (Delcourt et al. 2021). In some stands, Scots pine co‐occurred with larch, and lichen could be found in addition to shrubs and mosses. Roughly 30% of the area of Yert fire consisted of larch‐pine forests. The Batamay burn scar lies within the Central Yakutian Lowland, a vast depression stretching along the Lena river and its tributaries (Chevychelov and Bosikov 2010). The Batamay burn scar is situated around 15 km south of the Verkhoyansk range foothills, with elevations ranging between 80 and 160 m above sea level at our plots. The Yert burn scar is located 200 km west of Yakutsk in the Near Lena Plateau where elevations averaged 230 m at the study plots. Mean slope, calculated from a 32 m digital elevation model (ArcticDEM Mosaics v4.1) (Porter et al. 2023), at the study plots was 2.4°. Two‐thirds of sampled forest stands were located on well‐drained slopes, or flat to gentle sloping terrain with noticeable surface moisture, while the remaining occupied shallow depressions with moderate to considerable surface moisture. The climate in the region is strongly continental, with very low winter and relatively high summer temperatures, and small amounts of precipitation (Chevychelov and Bosikov 2010). A detailed description of the study areas can be found in Delcourt et al. (2021).

**FIGURE 2:**
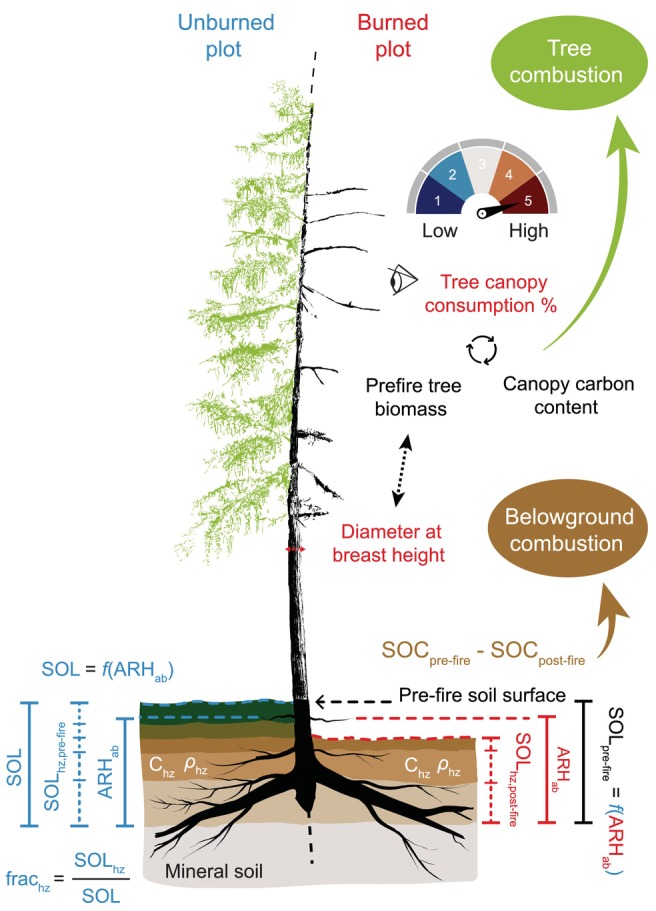
Reconstruction of pre‐fire carbon stocks and estimation of combustion from vegetation and belowground pools in burned plots. Tree combustion was calculated from pre‐fire biomass (derived from diameter‐based allometric equations), visually assessed canopy consumption, and canopy carbon content. For belowground pools, measurements from unburned plots were used to reconstruct pre‐fire soil organic carbon content (SOC) in burned plots. Belowground combustion was estimated as the difference between reconstructed pre‐fire SOC and post‐fire residual SOC. Variables measured in burned and unburned plots are shown in red and blue, respectively. See Section 2.2 for methodological details. SOL, soil organic layer depth (cm); hz, soil horizon; frac, fractional depth (ratio of horizon depth to total soil organic layer depth); ARH_ab_, adventitious root height above mineral soil (cm); C, carbon content (g C kg^−1^); *ρ*, bulk density (g cm^−3^); SOC, soil organic carbon content (kg C m^−2^).

Our approach aimed to collect measurements in burned plots that allow reconstruction of pre‐fire C pools, including vegetation, woody debris, and soil organic layers (Figure 2). In total, we sampled 41 burned plots and conducted control measurements in 12 unburned plots located in the vicinity. Burned plots were selected within a wide range of fire severity, stand age, species composition, and landscape position based on visual assessment and geospatial layers. For fire severity, we used dNBR layers computed using pre‐fire and post‐fire Sentinel‐2 imagery from one year prior to and after the fire events. Selected burned plots were classified into three dominant pre‐fire vegetation types based on significant differences in stand structure, species composition, and soil properties (Figure S1). Burned plots where larch trees were intermixed with Scots pine were classified as ‘mixed’. Despite the relatively low number of these plots (*n* = 4), they were included in a separate group because vegetation and soil conditions considerably differed from those of larch‐dominated stands. The latter were further divided into two groups using an agglomerative clustering technique with larch proportion, tree density (i.e., stems per area), and stand age as inputs (Delcourt et al. 2021). In total, 17 burned plots were assigned to dense, young‐aged (50–70 years old) larch‐dominated stands (‘larch dense’), and 20 burned plots were assigned to more open and mature (110–130 years old) larch‐dominated stands (‘larch open’). See Supporting Information Methods for information on stand age determination. This classification was integral to our methodology for estimating pre‐fire aboveground and belowground C stocks (see Section 2.2). It was also motivated by field observations of differences in fire severity between forest types and provided a framework for illustrating the influence of pre‐fire stand structure on fuel consumption. Control plots were selected to best match conditions at sampled burned plots, in terms of stand structure, dominant tree species, and landscape position: six were in ‘larch dense’, three in ‘larch open’, and three in ‘mixed’ forests. Rather than being paired with individual burned plots, control plots were pooled per forest type to derive pre‐fire vegetation and soil conditions. For each plot, we established a 30 m by 30 m quadrant, in which measurements were taken along a 2 m by 30 m belt transect oriented in the north–south direction through the plot's center, following a design used to investigate North American boreal forest fires (Boby et al. 2010; Dieleman et al. 2020; Rogers et al. 2014).

### 2.2 Carbon Combustion Estimates

#### 2.2.1 Aboveground Combustion

In each plot, we inventoried every tree and shrub inside the belt transect of the following dominant species: Cajander larch (*L. cajanderi*), Scots pine ( *P. sylvestris* ), silver birch ( *Betula pendula* ), alder (*Alnus* spp.), and willow (*Salix* spp.) species. We measured diameter at breast height (DBH, 1.3 m above ground) for each individual stem ≥ 1.3 m tall and basal diameter (D_0_) for stems < 1.3 m in height. We used a number of species‐specific allometric equations developed from naturally regenerated stands in Siberia, as well as from managed stands in northern Europe and the USA (Table S1). These equations relate tree diameter (DBH or D_0_) to the pre‐fire biomass associated with each tree component, including stem wood, stem bark, branches, and foliage. We developed new allometric equations for *P. sylvestris* based on measurements of 41 trees retrieved from Schepaschenko et al. (2017) using the same approach as in Delcourt and Veraverbeke (2022). See Supporting Information for further details about the development of these equations (Figures S2 and S3, Table S2). When several equations were available for a given species, we computed the average biomass. Dry mass values were then converted to C content using C concentrations of 0.47 for stem wood and bark, 0.48 for branches, and 0.46 for cones and needles (Alexander et al. 2012). We also visually assessed fire consumption of aboveground tree biomass using an approach similar to Rogers et al. (2014) and Dieleman et al. (2020), in which each tree was scored on a discrete scale spanning the possible range of fire consumption from low to high. In this study, we used a 12‐point scale, whereby each score was translated into fractional consumption for each tree component (Figure S4). The resulting consumption rates were multiplied with C content to estimate C consumption for each individual tree and summed up over the whole transect to derive vegetation C consumption per unit area.

We used the line‐intersect method (Brown 1971; Brown et al. 1982; Brown and Roussopoulos 1974; Van Wagner 1968, 1982; Warren and Olsen 1964) to estimate pre‐fire biomass of all dead and down woody material on the ground within each plot. This involved tallying every piece that intersected a transect line (i.e., the west side of our belt transect) using size classes for fine woody debris (FWD, ≤ 7 cm in diameter) and decay classes for coarse woody debris (CWD, > 7 cm). A detailed description of the approach used to determine fuel loads is provided in Supporting Information Methods (Tables S3 and S4). We visually assigned percentages of FWD and CWD consumption to each burned plot, which were multiplied by pre‐fire C stocks to determine plot‐level woody debris C consumption.

#### 2.2.2 Belowground Combustion

To quantitatively reconstruct C combustion of organic soils in burned plots, we combined measurements of residual C stocks with estimates of pre‐fire C stocks. Soil cores were taken every 7.5 m along each transect, resulting in a total of five cores per plot. Where possible, cores were taken at the base of the nearest tree as close to the bole as possible. Because sampling only at tree bases might bias the estimation of residual soil organic layer depth (Boby et al. 2010), at least two cores per transect were taken away from trees (i.e., more than 70 cm away from tree bases). First, a larger 20 cm by 20 cm block of soil was excavated with a shovel to the depth of the mineral soil to avoid compaction of the soil organic material. For each excavated soil core, we measured the distance from the surface to the mineral soil and the depth of the following soil organic horizons according to the classification of Manies et al. (2004): litter, live moss, lichen, dead moss, fibric, mesic, and humic. A 10 cm by 10 cm subset of the soil block was sampled, and soil horizons were separated using sharp serrated knives. Biomass that had accumulated after the fire event, such as regenerating vegetation and unscorched litter, was removed from the surface of the soil core at burned plots. Soil materials were wrapped in aluminum foil and kept cool until processed in the laboratory to determine bulk density and C concentration.

A total of 253 soil cores comprising 382 individual horizons were collected from burned and unburned plots. Materials greater than 2 mm in diameter, including roots, twigs, and gravels, were manually removed from soil samples, which were then oven dried at 65°C to constant weight. Bulk density was estimated as the ratio of the soil sample dry mass to the soil sample volume as measured in the field. After grinding and homogenization, soil samples were processed using an elemental analyzer (FlashEA 1112, Thermo Fisher Scientific, Waltham, MA, USA) to determine total C concentration (g C per kg of dry sample weight). Calibration was performed using internationally distributed reference materials, with two blanks and two standard samples analyzed every 25 runs.

For each soil core, we obtained pre‐fire soil organic layer depth (SOL_pre‐fire_, cm) from Delcourt et al. (2021). Delcourt et al. (2021) used the position of adventitious roots growing below the soil surface as a proxy for pre‐fire soil surface height. In response to wet and cold soil conditions, these fine lateral roots develop above the main root collar system and may still be visible many years after the fire. This method has been well established throughout boreal North America (Boby et al. 2010; Rogers et al. 2014; Walker, Baltzer, et al. 2018; Walker, Rogers, et al. 2018; Walker et al. 2019; Dieleman et al. 2020) and successfully deployed in eastern Siberia (Delcourt et al. 2021). In unburned plots, the position of the uppermost adventitious root below the organic soil surface (ARH_0_, cm) and the soil organic layer depth (SOL, cm) were recorded for each soil core made at the base of a tree. Using all unburned soil cores (*n* = 148), a linear relationship was derived as follows (Delcourt et al. 2021):

(1) $$\mathrm{SOL}=0.96\times {\mathrm{ARH}}_{\mathrm{ab}}+3.91$$

where ARH_ab_ is the adventitious root height above mineral soil (cm) defined as

(2) $${\mathrm{ARH}}_{\mathrm{ab}}=\mathrm{SOL}-{\mathrm{ARH}}_{0}$$

In burned plots, Equation (1) was applied to estimate SOL_pre‐fire_ at soil cores next to trees, and the plot average was used at cores away from trees. Delcourt et al. (2021) estimated SOL_pre‐fire_ and burn depth at the base of ten additional trees per plot, randomly selected outside the belt transect, to account for spatial variability in soil C distribution within plots.

In this study, pre‐fire organic soil C content (kg C m^−2^) was estimated at each soil core as follows:

(3) $$\text{Organic soil}\;C_{\mathrm{pre}‐\text{fire}}={\mathrm{SOL}}_{\mathrm{pre}‐\text{fire}}\times \sum_{\mathrm{hz}}{\text{frac}}_{\mathrm{hz}}^{\text{control}}\times \rho_{\mathrm{hz}}^{\text{control}}\times C_{\mathrm{hz}}^{\text{control}}$$

where ${\text{frac}}_{\mathrm{hz}}^{\text{control}}$, $\rho_{\mathrm{hz}}^{\text{control}}$, and $C_{\mathrm{hz}}^{\text{control}}$ are the fractional depth (i.e., ratio of horizon depth to total soil organic layer depth), bulk density (g cm^−3^), and C concentration of the soil horizon hz, respectively (Figure 2). These parameters were derived from measurements at unburned plots and are provided in Table 1. We calculated residual organic soil C content (kg C m^−2^) for each soil core as follows:

(4) $$\begin{array}{c} \text{Organic soil}\;C_{\text{post}‐\text{fire}}=\sum_{\mathrm{hz}}{\mathrm{SOL}}_{\mathrm{hz}}^{\text{post}}\times \rho_{\mathrm{hz}}^{\text{post}}\times C_{\mathrm{hz}}^{\text{post}} \end{array}$$

where ${\mathrm{SOL}}_{\mathrm{hz}}^{\text{post}}$, $\rho_{\mathrm{hz}}^{\text{post}}$, and $C_{\mathrm{hz}}^{\text{post}}$ are the depth (cm), bulk density (g cm^−3^), and C concentration of the residual soil horizon hz. C combustion was determined as the difference between pre‐fire and post‐fire organic soil C. In this calculation, we did not allow physically impossible negative combustion values. In the rare cases this occurred, the combustion value was set to zero. Using the C combustion estimates derived from all soil cores in burned plots (*n* = 109), we found a strong linear relationship between burn depth and soil C combustion (*R* ^2^ = 0.89, *p*‐value < 0.001; Figure S5). The mean belowground combustion at a given plot was finally computed as the average of (1) mean combustion from the soil cores within the belt transect and (2) combustion derived by applying this relationship to the burn depth at the ten trees outside the transect. All data analyses were performed using R statistical software version 4.4.2 (R Core Team 2024). Significant differences in carbon stocks and combustion between forest types were assessed using a one‐way analysis of variance (ANOVA) followed by a Tukey–Kramer post hoc analysis for multiple comparisons. In cases where the assumptions of ANOVA were not fulfilled, we used the non‐parametric Kruskal‐Wallis test and the Wilcoxon rank sum test for pairwise comparisons with Benjamini‐Hochberg corrections for multiple testing as implemented in the R ‘stats’ package (R Core Team 2024).

**TABLE 1:** Organic soil characteristics by horizon and forest types derived from unburned plots.

Soil horizon	Fraction of total depth (*n*)			Bulk density (g cm^−3^) (± SD)	C concentration (g C kg^−1^) (± SD)	Number of samples
	Yert (mesic)	Yert (no mesic)	Batamay			
*Larch dense*						
Live moss		0.06 (45)	0 (45)	0.016 (± 0.008)	401.6 (±69.7)	4
Litter		0.29 (45)	0.37 (45)	0.036 (± 0.016)	422.9 (±57.9)	25
Dead moss		0.39 (45)	0.05 (45)	0.045 (± 0.024)	371.4 (± 60.1)	11
Fibric		0.26 (45)	0.58 (45)	0.153 (± 0.079)	258.7 (± 78.7)	22
Mesic		0 (45)	0 (45)	0.534 (± 0.247)^a^	69.9 (± 30.7)^a^	10^a^
*Larch open*						
Live moss		0.02 (30)	0 (15)	0.016 (± 0.008)	401.6 (± 69.7)	0
Litter		0.23 (30)	0.37 (15)	0.036 (± 0.016)	422.9 (±57.9)	14
Dead moss		0.33 (30)	0.05 (15)	0.072 (± 0.030)	371.4 (± 60.1)	9
Fibric		0.42 (30)	0.58 (15)	0.153 (± 0.079)	258.7 (± 78.7)	14
Mesic		0 (30)	0 (15)	0.534 (± 0.247)^a^	69.9 (± 30.7)^a^	22^a^
*Mixed larch/pine*						
Live moss	0 (15)	0.05 (30)		0.016 (± 0.008)	401.6 (± 69.7)	2
Litter	0.68 (15)	0.42 (30)		0.063 (± 0.019)	422.9 (± 57.9)	15
Dead moss	0 (15)	0.05 (30)		0.057 (± 0.029)	371.4 (± 60.1)	0
Fibric	0.25 (15)	0.48 (30)		0.296 (± 0.157)	169.7 (± 90.5)	13
Mesic	0.07 (15)	0 (30)		0.254 (± 0.102)^a^	182.0 (± 107.9)^a^	6^a^

*Note:* These parameters were used to reconstruct pre‐fire organic soil carbon (C) stocks in burned plots. For averaged fractional depth, the number of measurements (soil cores and soil profiles at tree base) is given in parentheses. The two fire scars differed in soil horizon distributions: Yert plots were grouped based on the presence or absence of mesic horizons in the soil column, while Batamay plots were treated separately due to their distinct soil profiles (namely, the absence of live moss layer and a thin or absent dead moss layer). Note that bulk density and C concentration did not differ significantly between fire scars. These parameters are reported as different between forest types only when control plots exhibited significantly different means. SD, standard deviation.

^a^ Since mesic layers were very rare in unburned plots, the values provided here were derived from burned plots.

### 2.3 Uncertainties

We adopted a Monte Carlo framework to quantify uncertainties in our estimates of carbon stocks and combustion, similar to Rogers et al. (2014), Walker, Rogers, et al. (2018), and Dieleman et al. (2020). For each C pool, we identified the major sources of error associated with our approach. The three sources we identified for trees were (i) the available allometric equations, (ii) the percentage C of tree biomass, and (iii) our method for visually estimating the extent of biomass consumption for individual trees. For woody debris, uncertainty was assumed to come from the parameters used to calculate pre‐fire (i) FWD and (ii) CWD biomass from the line‐intersect method, (iii) the percentage C of woody debris biomass, and (iv) our method for visually estimating plot‐level woody debris consumption. For belowground, we assumed uncertainty to come from (i) the adventitious root height approach used to determine pre‐fire soil organic layer depth, (ii) the pre‐fire bulk density and C concentration values derived from unburned plots, (iii) the calculation of soil cores combustion, and (iv) the calculation of soil combustion at tree bases outside the belt transect using burn depth measurements. A total of 1000 simulations were performed in which inputs varied by either the distribution of influential parameters or methodological choices. For example, we systematically varied the percentage C of tree components within a normal distribution centered on literature‐derived values with a standard deviation of 3%. Additional details about the probability distributions and the alternative approaches used to introduce systematic errors in our simulations are provided in Tables S5 and S6. Uncertainty was estimated as the standard deviation of the combustion estimates obtained from the 1000 Monte Carlo runs. We also conducted a series of simulations to isolate the individual contributions of each C pool to overall uncertainty. Each series consisted of the following scenarios: (1) the so‐called “source removed” scenario, where the influence of the C pool was excluded across the 1000 iterations by maintaining constant values and/or methods for all associated sources of error; and the “source only” scenario, where the C pool exclusively influenced the outcome. Finally, this approach was applied within each C pool to identify the most influential sources of uncertainty impacting our combustion estimates.

### 2.4 Drivers of Combustion

We hypothesized that combustion was driven by plot‐level attributes, pre‐fire stand composition and structure, fire characteristics, and weather conditions, or some combination of these factors. Our combustion metrics included aboveground, belowground, and total C combustion, expressed both in absolute terms and relative to pre‐fire C pools. The influence of fire‐conducive weather was explored using the Canadian Forest Fire Weather Index (FWI) System. Originally developed to estimate fire danger in Canadian boreal forests, the FWI System consists of three fuel moisture codes and three fire behavior indices that have also proven useful for predicting fire activity and C emissions globally (de Groot et al. 2009; Flannigan et al. 2013). The fuel moisture codes, Fine Fuel Moisture Code (FFMC), Duff Moisture Code (DMC), and Drought Code (DC), represent the moisture content of surface litter and other fine fuels (1–2 cm depth), intermediate layers of loosely compacted organic material (5–10 cm depth), and deep layers of compact organic material (10–20 cm depth). The Initial Spread Index (ISI) estimates the rate of fire spread based on FFMC and wind speed, while the Buildup Index (BUI), derived from DMC and DC, indicates fuel availability for combustion. The Fire Weather Index (FWI) combines ISI and BUI to provide an overall indicator of fire intensity (Stocks et al. 1989; Van Wagner 1987; Wotton 2009). Additionally, the Daily Severity Ranking (DSR), a power function of FWI, represents the difficulty of suppressing fires. For each plot, we obtained FWI System components from the Global Fire WEather Database (GFWED) (Field et al. 2015), gridded at 0.5° latitude by 2°/3° longitude resolution, based on the plot location and the day of burn (DOB). FWI System calculations use daily weather variables (i.e., temperature, relative humidity, wind speed, and snow depth) from the Modern‐Era Retrospective Analysis for Research and Application version 2 (MERRA‐2). In this study, we used the set of calculations in which precipitation accumulated over the previous 24 h was gauge‐corrected. DOB was retrieved for each plot from the nearest active fire detection using the global monthly Visible Infrared Imaging Radiometer Suite (VIIRS) product (VNP14IMGML) at 375 m resolution (Schroeder et al. 2014). Our field plots in both fire scars were located within four separate grid cells of the GFWED product and spanned 12 different burn dates, resulting in 15 distinct values for each FWI System component. Additionally, we calculated the vapor pressure deficit (VPD), which represents the difference between the saturation vapor pressure and the actual vapor pressure at a given temperature. VPD describes the ability of the atmosphere to extract moisture from the land surface and has been used to analyze fire regimes in boreal forest ecosystems (Scholten et al. 2024; Sedano and Randerson 2014). We calculated VPD from the MERRA‐2 air temperature and relative humidity with the following expressions (Sedano and Randerson 2014):

(5) $$\mathrm{VPD}=\left(100-\mathrm{RH}\right)\times \mathrm{SVP}$$

where RH is the relative humidity (%) and SVP is the saturation vapor pressure computed as follows:

(6) $$\mathrm{SVP}=0.6107\times 10^{\left(\frac{7.5\times T}{T+237.3}\right)}$$

where *T* is surface air temperature (°C).

We first examined linear relationships between our six combustion metrics and a set of hypothesized drivers, including seven plot attributes, 16 pre‐fire stand composition variables, and 13 fire attributes (Table S7). To address model assumptions, absolute and relative aboveground combustion variables were transformed using Tukey Ladder of Powers (*𝑋*_*𝜆*_, 𝜆 = 0.35 and 𝜆 = 0.45 for absolute and relative aboveground combustion), with the ‘transformTukey’ function from the ‘rcompanion’ R package (Mangiafico 2025). Candidate predictors were tested for collinearity (Spearman's *p*‐value < 0.05) and excluded if significant correlations were found. We defined a set of candidate models to reflect our hypotheses about the main drivers of combustion (Table 2), and fitted a simple linear regression for each model. We conducted model selection using the size‐corrected Akaike information criterion (AICc) as implemented in the ‘AICcmodavg’ R package (Mazerolle 2023). A model was considered superior if it showed a decrease in AICc by 2 units or more and maximized AICc model weights (Dieleman et al. 2020). Residual plots were examined to verify homoscedasticity and normality.

**TABLE 2:** Variables included in each of the candidate models to predict absolute and relative aboveground C combustion (Ca, pCa), absolute and relative belowground C combustion (Cb, pCb), and absolute and relative total C combustion (Ct, pCt).

Model	Variables
Null model	None
M1: Plot attributes	Stand age
	Pre‐fire soil organic layer depth
	Condensed moisture class
M2: Pre‐fire stand composition	Pre‐fire tree biomass
	Cajander larch proportion
M3: Fire attributes	Fire Weather Index (FWI)
M4: Plot attributes + Pre‐fire stand composition	M1 + M2
M5: Plot attributes + Fire attributes	M1 + M3
M6: Pre‐fire stand composition + Fire attributes	M2 + M3
Full model	M1 + M2 + M3

### 2.5 Spatial Modeling

To spatially extrapolate our estimates of combustion over the Batamay and Yert fire scars, we developed a multiple linear regression model following Walker, Rogers, et al. (2018). We acquired 36 initial predictors related to topography, fire severity, burn date, tree cover, fire weather, and soil properties (Table S8). For each plot, values were extracted using weighted averages of pixels covering our 30 m by 30 m quadrants. We excluded variables that were significantly correlated with one another (Spearman's *p*‐value < 0.05) within each predictor set before fitting separate multiple linear regression models. Significant variables (*p*‐value < 0.05) within each predictor set were included in the final model. Model reduction was performed using backward stepwise selection based on the Akaike information criterion (AIC). We examined residual plots to test for heteroscedasticity and non‐normality. To test against overfitting, we conducted a 10‐fold cross validation, repeated 100 times. Final model predictors were re‐gridded to 30 m on a WGS 84 UTM Zone 52N projection (EPSG:36652) using bilinear interpolation. For consistency, we re‐ran our regression model with these downscaled variables. The regression model was finally applied to all pixels contained within both fire scars. A Monte Carlo framework was used to quantify uncertainty from our spatial modeling, combining the plot‐level uncertainty estimates described in Section 2.3 with uncertainty from our spatial model to predict combustion at any given 30 m pixel. For each of 1000 Monte Carlo simulations, trees, woody debris, and belowground combustion across all plots were adjusted based on their standard deviations. For each C pool, a random number was generated from a normal distribution, multiplied by plot‐level combustion uncertainty, and then added to our original estimate of plot‐level combustion. We derived a new regression model for each simulation based on updated estimates of combustion in each plot. To account for prediction uncertainty, we applied the updated model to each pixel and added an error term drawn from the normal distribution of residual model errors.

## 3 Results

**FIGURE 3:**
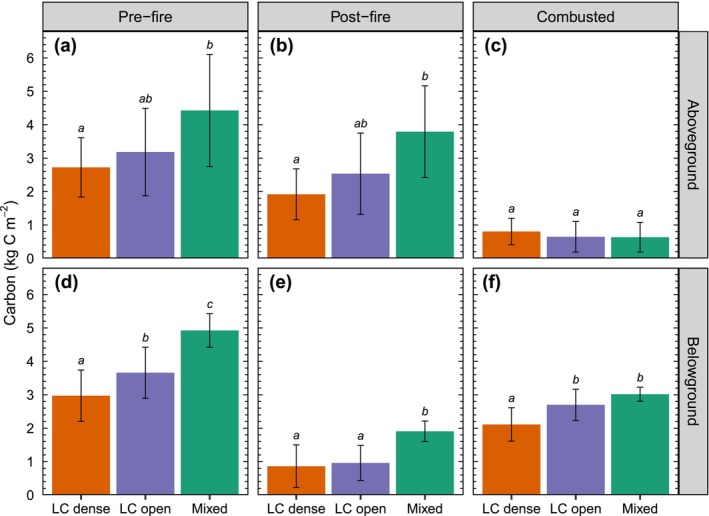
Average aboveground (vegetation and woody debris) and belowground pre‐fire (a, d), post‐fire (b, e), and combusted (c, f) carbon (C) across forest types (*n* = 17 for dense plots, *n* = 20 for open plots, and *n* = 4 for mixed plots). Error bars represent one standard deviation. Letters (a, b, c) indicate significant differences between forest types based on either a one‐way analysis of variance (ANOVA) followed by the Tukey–Kramer test (a, b, f), or the non‐parametric Kruskal‐Wallis and Wilcoxon rank sum tests in cases where the assumptions of ANOVA were not fulfilled (c–e). LC, *Larix cajanderi*.

Estimated pre‐fire C stocks in our 41 burned plots were 3.12 ± 1.26 kg C m^−2^ (mean ± standard deviation) and 3.50 ± 0.93 kg C m^−2^ in aboveground (trees and woody debris) and belowground pools. The ‘larch dense’ forest type was characterized by larch‐dominated stands (mean larch proportion = 80%), young aged (mean = 55 years old), and high tree density (mean = 24,700 trees ha^−1^) stands, while the ‘larch open’ type consisted of more mature (112 years old) and open (10,900 trees ha^−1^) forests. Mixed larch/pine stands (mean larch proportion = 30%) averaged 131 years old and were found on drier upland sites (Table S9). Despite differences in stand structure, species composition, and landscape position, the relative distribution of aboveground and belowground C showed no significant differences across the three forest types, with organic soil layers contributing an average of 54% of the total pre‐fire C stock (Figure 3a,d). Estimated mean aboveground combustion across all plots was 0.71 ± 0.43 kg C m^−2^. Young and dense larch‐dominated stands experienced the highest aboveground fuel consumption, with a loss of approximately 30% of pre‐fire C stocks. Burn depth ranged between 4.1 and 12.5 cm, with a mean of 8.0 ± 1.8 cm at dense larch plots (*n* = 17), 10.0 ± 1.6 cm at open larch plots (*n* = 20), and 10.9 ± 0.7 cm at mixed plots (*n* = 4) (Table S9). This corresponded to a range of soil combustion estimates between 0.99 and 3.43 kg C m^−2^, with a mean of 2.11 ± 0.50 kg C m^−2^ at dense larch plots, 2.70 ± 0.47 kg C m^−2^ at open larch plots, and 3.02 ± 0.21 kg C m^−2^ at mixed plots (Figure 3f). Fires consumed an average of 3.20 ± 0.75 kg C m^−2^ across our 41 burned plots, of which on average 78% (2.49 ± 0.56 kg C m^−2^) stemmed from organic soils.

**FIGURE 4:**
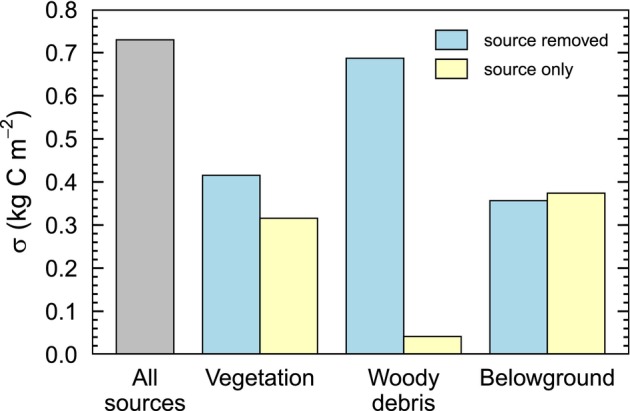
Attribution of uncertainty in total carbon (C) combustion estimates using a Monte Carlo framework. Uncertainty sources were grouped by the three main carbon pools contributing to combustion. In the all‐sources scenario (gray), uncertainty was introduced across all relevant parameters and methodological choices. For each pool, two additional scenarios were simulated: “source removed” (blue), in which the values and methods for the source category remained constant as in the main approach, and “source only” (yellow), in which the source category exclusively influenced the outcome. The standard deviation (𝜎) of combustion estimates from 1000 runs was calculated for each scenario.

Monte Carlo simulations, accounting for all sources of error in aboveground and belowground combustion, resulted in an uncertainty of 0.73 kg C m^−2^ for total combustion estimates across field plots (Figure 4). Systematic errors from organic soil and tree combustion were relatively similar, with total contributions of 0.37 and 0.32 kg C m^−2^. The average combustion estimate over 1000 runs was 3.33 kg C m^−2^, or 4.2% higher than the value obtained from our main approach. The primary contributors to uncertainty within each C pool were the allometric equations used to predict pre‐fire tree biomass, plot‐level visual estimates of woody debris consumption, and measurements of bulk density and C concentration in unburned plots (Figure S6).

**FIGURE 5:**
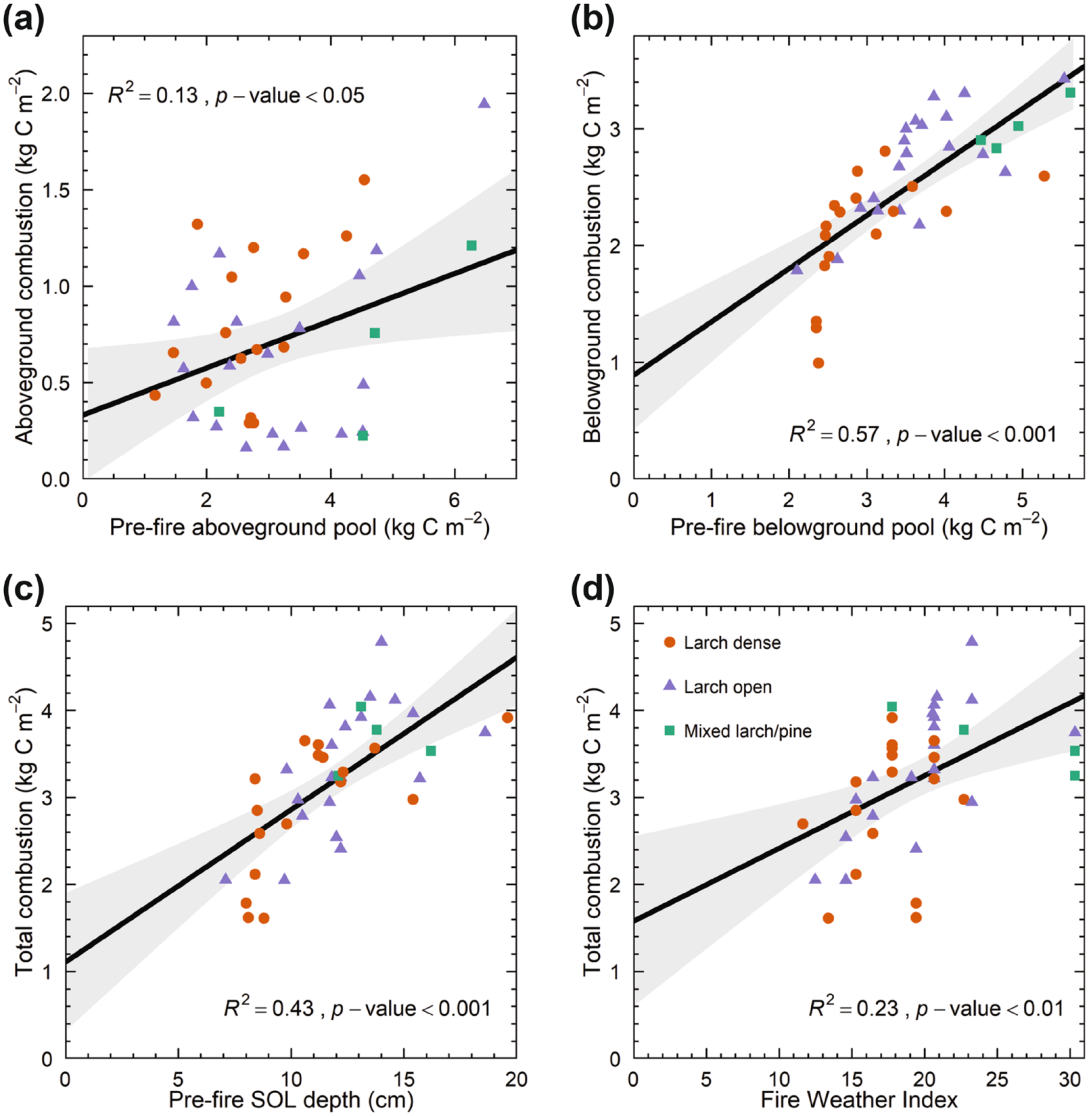
Relationships between plot‐level carbon (C) combustion estimates and key explanatory variables. (a) Aboveground combustion as a function of estimated pre‐fire aboveground C pool, (b) belowground combustion as a function of estimated pre‐fire belowground C pool, and total combustion as a function of (c) estimated pre‐fire soil organic layer (SOL) depth and (d) Fire Weather Index. The shaded area indicates the 95% confidence interval.

The strongest predictors for aboveground and belowground combustion were the corresponding pre‐fire C pools (Figure 5a,b). Belowground combustion was positively related to stand age (*R* ^2^ = 0.36, *p*‐value < 0.001), and all components of the FWI System, except for DMC (Table S7). Additionally, we found negative relationships between relative aboveground and belowground combustion and their corresponding pre‐fire C pools. Total combustion rates increased significantly with pre‐fire soil depth (*R* ^2^ = 0.44, *p*‐value < 0.001; Figure 5c) and pre‐fire soil C stock (*R* ^2^ = 0.40, *p*‐value < 0.001) across all plots. To a lesser extent, stand age and pre‐fire aboveground C stock also influenced total C combustion (Table S7). Total C combustion was positively related to all fuel moisture codes and fire behavior indices, with FWI showing the highest explanatory power (Figure 5d). The proportion of total pre‐fire C combusted decreased with tree basal area (*R* ^2^ = 0.28, *p*‐value < 0.001) and aboveground C pool (*R* ^2^ = 0.33, *p*‐value < 0.001). The fraction of combustion from organic soils increased with stand age (*R* ^2^ = 0.15, *p*‐value < 0.05) and decreased with the proportion of Cajander larch trees (*R* ^2^ = 0.17, *p*‐value < 0.01). Organic soils in mixed forests, where larch proportion averaged 29%, accounted for up to 83% of total C combustion. Of the tested hypotheses, the best‐supported model for predicting total C combustion was M4 (Table 3), which included covariates for plot attributes and pre‐fire stand composition (Table 2). The model including only plot‐level variables (M1) was the best for predicting belowground combustion and performed similarly to M4 for total combustion (ΔAICc < 2; Table 3). Across all combustion metrics, the only models with any probability (wi > 0) were M4 and the full model (Table 3, Table S10). Model selection indicated that fuel conditions and weather at the time of fire co‐influence aboveground and belowground combustion with pre‐fire stand composition (M6) and plot attributes (M5), respectively.

**TABLE 3:** Results of AICc‐based model selection assessing the relationships between response variables of total (Ct), aboveground (Ca), and belowground (Cb) carbon (C) combustion to a set of simple linear regression.

Model	*K*	Total C combustion (Ct)					Aboveground C combustion (Ca)					Belowground C combustion (Cb)				
		AICc	ΔAICc	wi	LogL	*R* ^2^	AICc	ΔAICc	wi	LogL	*R* ^2^	AICc	ΔAICc	wi	LogL	*R* ^2^
M4	8	**71.03**	**0**	**0.58**	**−25.19**	**0.62**	**−18.27**	**1.43**	**0.21**	**19.46**	**0.40**	34.31	5.65	0.04	−6.83	0.73
M1	6	**72.86**	**1.84**	**0.23**	**−29.16**	**0.54**	−8.91	10.79	0	11.73	0.11	**28.66**	**0**	**0.66**	**−7.06**	**0.73**
Full	9	74.32	3.29	0.22	−25.16	0.62	−15.23	4.47	0.05	19.62	0.40	36.15	7.50	0.02	−6.08	0.74
M5	7	75.29	4.26	0.07	−28.89	0.55	−5.96	13.74	0	11.73	0.11	**30.30**	**1.65**	**0.29**	**−6.40**	**0.74**
M6	5	85.38	14.36	0	−36.81	0.33	**−19.70**	**0**	**0.42**	**15.71**	**0.24**	58.19	29.53	0	−23.24	0.41
M3	3	85.99	14.97	0	−39.66	0.23	−13.88	5.82	0.02	10.26	0.01	59.99	31.33	0	−26.67	0.31
M2	4	93.74	22.72	0	−42.30	0.12	**−18.62**	**1.08**	**0.24**	**13.86**	**0.17**	66.18	37.52	0	−28.54	0.24
Null	2	93.86	22.83	0	−44.77	0	−15.72	3.98	0.06	10.02	0	72.62	43.96	0	−34.15	0

*Note:* Variables included in each model are listed in Table 2. For each model, the number of parameters (*K*), the sample size corrected Akaike information criterion (AICc), the change in AICc relative to the best model (ΔAICc), the model weight (wi), the Log‐Likelihood (LogL), and the marginal *R* ^2^ are given. Bold indicates the most probable models based on ΔAICc < 2.

**FIGURE 6:**
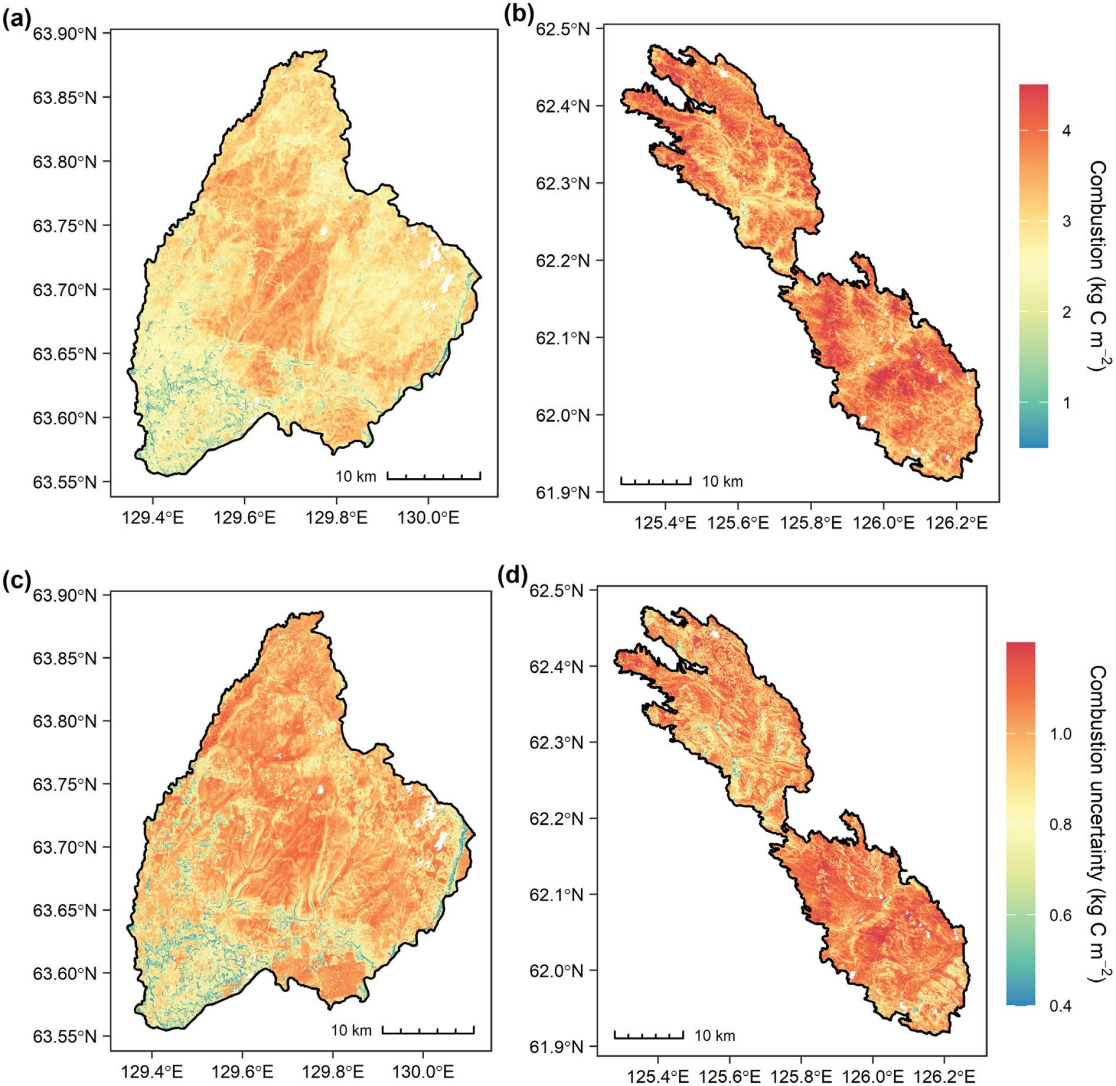
Maps of modeled total carbon (C) combustion (a, b) and associated uncertainty (c, d) within the Batamay (left) and Yert (right) fire perimeters.

The final spatial model predicted total combustion (kg C m^−2^) over the Batamay and Yert fire scars as a function of slope, dNBR, and percent sand in the top 15 cm of soil (Figure S7), and performed adequately (adjusted *R* ^2^ = 0.47, overall *R* ^2^ = 0.51, 10‐fold cross validation *R* ^2^ = 0.40) (Figure S8). Mean combustion across the Batamay and Yert fires was 2.90 and 3.67 kg C m^−2^, respectively (Figure 6). The model indicated an overarching pattern of high C combustion over the Yert burn scar (standard deviation = 0.48 kg C m^−2^; Figure 6b). In contrast, modeled C combustion displayed more spatial variability in the Batamay burn scar (standard deviation = 0.57 kg C m^−2^), with a core area of high combustion located in the center and lower values in the southwestern portion of the fire scar (Figure 6a). Mean pixel‐level combustion inside both fires was slightly higher than mean plot‐level combustion (3.31 kg C m^−2^ vs. 3.20 kg C m^−2^). Mean pixel‐level uncertainty from the 1000 Monte Carlo simulations was 0.97 kg C m^−2^.

## 4 Discussion

Although most boreal burned area occurs in Eurasia, there is a large paucity of field‐based combustion estimates in this region compared to boreal forests in Alaska and Canada (Veraverbeke et al. 2021). Hence, the magnitude, variability, and drivers of C combustion in Eurasia remain relatively unknown compared to boreal North America. Efforts have been made to quantify fuel consumption from experimental fires of varying intensity in Scots pine and larch‐mixed forests in central and southern Siberia (Ivanova et al. 2011, 2020; McRae et al. 2006). These surface fires, which consumed only down woody debris, litter, and belowground fuels, resulted in an average combustion of 0.8 kg C m^−2^. The lower estimates in these fires, compared to our study, can be attributed to a combination of lower pre‐fire fuel loads, burn depth, and combustion efficiency. Pre‐fire surface and ground fuel loads in these forests averaged approximately 2.5 kg C m^−2^, which is at the lower end of the range of our estimates (2.22–6.03 kg C m^−2^). Additionally, indicators of fire severity, such as burn depth and reduction in pre‐fire fuel loads, averaged 9.3 cm and 71% in our plots compared to 0.9–6.6 cm and 16%–74% in the light‐coniferous forests of central Siberia. Kukavskaya et al. (2017) studied the impact of surface fires in dark‐coniferous stands dominated by Siberian pine ( *Pinus sibirica* Du Tour), where ground fuel consumption averaged 1.69 kg C m^−2^. Dark‐coniferous forests are less likely to burn because they occur in areas of relatively higher water supply, such as river valleys or mountainous regions. However, extensive drought periods can result in large fires in these forests (Kharuk et al. 2021). Recent studies assessed C combustion in Scots pine forests following the extreme fire events of 2014 and 2018 in Sweden (Eckdahl et al. 2022; Granath et al. 2021; Gustafsson et al. 2019; Kelly et al. 2021). These fires exhibited substantial canopy fire spread, surface vegetation and litter removal, and deeper smoldering combustion, with combustion estimates ranging between 0.63 and 4.50 kg C m^−2^. The variability in estimates across different ecosystems highlights the need for ecosystem‐specific field data to better constrain regional estimates of C combustion in boreal Eurasia. Specifically, there is a lack of in situ measurements from key regions, such as larch forests, in which most of Siberian wildfires occur (Kharuk et al. 2021). Despite the importance of Siberian larch forests in the boreal biome, such data remain very scarce.

Our study highlights the potential of the adventitious root height approach to estimate C combustion in Siberian larch forests by reconstructing pre‐fire soil organic layers depth in 41 burned stands. By accounting for the significant variation in soil organic matter distribution at small spatial scales, this method provides an alternative to the common paired‐sample approach, where belowground fuel consumption is estimated as the difference between fuel loads in unburned sites and residual loads in burned sites. In this study, burned sites were sampled one to two years post‐fire, a time frame considered sufficient to minimize the influence of post‐fire vegetation recovery and organic matter accumulation on our estimates. This relatively short interval was partly determined by logistical constraints, but is consistent with other similar studies (Boby et al. 2010; Dieleman et al. 2020; Rogers et al. 2014; Walker, Baltzer, et al. 2018; Walker, Rogers, et al. 2018). While we acknowledge that additional C losses may have occurred through microbial respiration, leaching, and erosion, these processes were beyond the scope of this study, which focuses on combustion as the primary driver of C emissions. Our mean combustion estimate of 3.20 kg C m^−2^ is close to the estimate of 3.36 kg C m^−2^ reported in a recent study conducted in Cajander larch forests near our study plots (Webb et al. 2024). However, organic soils contributed only 13% of the total C loss compared to 78% in our plots. These discrepancies can result from differences in the characteristics of sampled sites. Pre‐fire aboveground and belowground pools averaged 3.12 and 3.50 kg C m^−2^ compared to 5.05 and 1.74 kg C m^−2^ in Webb et al. (2024). Remarkably, although these fires were classified as surface fires in this study, the results indicated that they consumed over 50% of the total pre‐fire aboveground biomass, more than twice the average proportion observed in our study plots. This contrasts with the relationships observed across our plots, where proportional aboveground combustion showed a negative relationship with pre‐fire aboveground biomass (Table S7). It is worth noting that methods used to estimate C combustion differed from our study, as site‐level C pool values between unburned and burned sites were compared using measurements collected 17 years after the fire. This approach may introduce bias in the estimation of C combustion, as post‐fire C accumulation may have occurred at different rates in early successional stands (i.e., burned sites) and late‐stage forests (i.e., unburned sites).

Although it is generally assumed that Siberian larch ecosystems rarely support crown fires due to the high moisture content of deciduous needles (de Groot et al. 2013), we found multiple examples of high‐severity crown fires in both fire scars, especially in young larch‐dominated stands (Figure S9a,b). Most of the forests burned in Siberia are affected by surface fires, yet the proximity of tree canopies in young and dense stands can promote the transition from surface to crown fires (Kharuk et al. 2021). This process is further enhanced in *L. cajanderi* forests, where successful regeneration after stand‐replacing fires leads to even‐aged or relatively even‐aged young stands with extremely high density (Abaimov et al. 1997). In some stands, we observed that the bark of larch trees peels off in stripes forming a fuel ladder (Figure S9c). This characteristic, documented for northern ecotypes in Siberia as a result of extreme growing conditions, could also promote crown fires (Wirth 2005). As fire return intervals in Siberian forests are expected to shorten with regional warming (Burrell et al. 2022; Ponomarev et al. 2016), the contribution of high‐intensity crown fires to regional fire‐induced C emissions may increase in the next decades. While young forests have smaller fuel loads, crown fires in these stands can still consume a substantial amount of pre‐fire C stocks. For example, high‐intensity crown fires consumed up to 76% of pre‐fire C pools in our young larch‐dominated plots. Our study also revealed that combustion from surface fires can be significant, mostly from belowground pools, in mature forest stands with greater pre‐fire fuel loads. The significant proportion of belowground C in total combustion observed within our plots aligns with findings from North American boreal forests. Walker, Rogers, et al. (2020) compiled C combustion estimates from 417 sites across 18 fire scars throughout the boreal forests in Alaska and Canada. They reported a mean combustion of 3.31 kg C m^−2^, 2.93 kg C m^−2^ from soils, and a mean burn depth of 10.2 cm. These values are comparable to mean soil combustion values of 2.70 and 3.02 kg C m^−2^ and mean burn depths of 10.0 and 10.9 cm found in open larch‐dominated and mixed forests, respectively (Table S9). The significance of belowground combustion in our study, likely driven by smoldering in deep organic soils, may explain why our estimates show combustion rates similar to those previously reported for North America, despite contrary expectations inferred from satellite observations (de Groot et al. 2013; Rogers et al. 2015). Smoldering combustion in pine forests of Sweden has also resulted in high combustion rates (Granath et al. 2021; Gustafsson et al. 2019). Kukavskaya et al. (2017) showed that fuel consumption in the dark‐coniferous forests of central Siberia was also driven by the rate of fire spread, with ground fuel consumption ranging between 1.01 kg C m^−2^ for a fast‐moving fire and 2.90 kg C m^−2^ for a slower‐moving fire of moderate to high severity. Our results, together with those from Webb et al. (2024), indicate that severe fires in Siberian larch forests can result in combustion rates comparable to those observed in boreal North America and exceeding those previously reported in Siberian light‐coniferous forests. This has implications for the regional C balance as fire severity in Siberian boreal forests is expected to increase with climate warming and increased drought events.

Our study aimed not only to quantify fuel consumption but also to provide insights into potential drivers of C combustion in Siberian larch forests. Weather conditions influence fuel dryness and flammability over short timescales and are thought to predominantly control C emissions (Amiro et al. 2009; Flannigan et al. 2005). However, fuel amount and structure, as well as topography, are also expected to play important roles in driving emissions from wildfires (Parisien et al. 2011; Walker, Rogers, et al. 2018). Fuel loading strongly influenced combustion across our plots, as shown by the significant relationships between aboveground, belowground, and total combustion with their corresponding pre‐fire C pools. Pre‐fire organic soil depth and C content were the most significant drivers of total combustion across our plots. This is consistent with Walker, Rogers, et al. (2020) who reported that belowground C was the strongest predictor of C combustion in Alaska and Canada. Pre‐fire stand composition has also been shown to influence fire severity and combustion metrics in the northwestern Canadian boreal forests (Walker, Rogers, et al. 2018; Whitman et al. 2018). In agreement, we found that higher pre‐fire tree basal area and aboveground C stocks were associated with lower proportions of pre‐fire C combusted. This pattern was further supported by our model selection results, which highlighted the importance of stand structure and species composition in predicting relative combustion (Table S10). We observed that lower‐severity surface fires were promoted in mature stands with larger trees, particularly in mixed forests, where relatively large post‐fire stocks remained. Stand age was also a significant driver of combustion, with combustion rates increasing in older stands. As stand age represents the time fuel has been accumulating since the last stand‐replacing disturbance, younger stands had accumulated relatively small carbon stocks, which in turn limited their combustion rates during the subsequent fire. Pre‐fire C stocks, pre‐fire soil organic layers depth, and burn depth all related positively to stand age (Figure S10). This may indicate that while young stands account for small amounts of total C emissions, they are likely to have relatively low post‐fire C stocks and thus may be more at risk of long‐term changes in ecosystem C dynamics. Such effects of increased fire frequency on the carbon storage of black spruce forests have been well documented (Brown and Johnstone 2011; Hoy et al. 2016; Walker, Rogers, et al. 2018).

We also found that soil combustion and total combustion were associated with components of the FWI system, with the FWI, a weather indicator of fire intensity, being the most significant factor. Siberian larch species are considered fire resistant, particularly due to the high moisture content of their needles (de Groot et al. 2013). However, with the expected intensification of regional warming and drying, our results suggest that the fire‐resisting properties of larch may be overcome by more extreme fire weather conditions. Indeed, we observed that fire weather conditions co‐influenced absolute and relative aboveground combustion (Tables 3 and S10). The results of our model selection highlight the complex interplay between environmental factors controlling fuel loading and weather patterns driving fuel availability for combustion. In contrast to the findings previously reported for the North American northwestern boreal forests (Walker, Rogers, et al. 2020), our results indicate a significant contribution of both fire weather conditions and landscape variables in predicting total C combustion. In the Walker, Rogers, et al. (2020) dataset, where most combustion stemmed from belowground pools, the variation in fuel loads and combustion was more important than fire weather effects. Therefore, the smaller variability in belowground fuel loading and consumption observed in our relatively small dataset may explain why fire weather was a more important driver of combustion compared to the findings from Walker, Rogers, et al. (2020). We acknowledge that logistical constraints limited our sampling to areas accessible by forest road or within a one‐day hike. Despite these limitations, our plots were carefully selected to ensure they were spaced several hundred meters apart, minimizing the risk of pseudoreplication in highly heterogeneous landscapes with variable fire behavior. Importantly, while our dataset represents a relatively small footprint, our field plots, collected in large fire events, are representative of the variability in fire severity, pre‐fire stand characteristics, and soil properties observed in eastern Siberian boreal forests (Figure S11). This supports the relevance of our findings for the broader Siberian larch forest ecosystems. Another limitation of our study is the coarser resolution of fire weather datasets compared to in situ measurements of C combustion and landscape predictors. While our study captures the spatial variation in fire weather indices between grid cells (coarse‐scale spatial variation) and the temporal variation associated with the day of burn, it does not characterize the fine‐scale spatial variation within grid cells. However, FWI System components have been shown to vary at synoptic scales of several hundreds of kilometers, suggesting that the spatial resolution of GFWED is suitable for capturing spatiotemporal variability in fire weather patterns (Walker, Rogers, et al. 2020).

To our knowledge, our study is the first to develop a statistical model to extrapolate combustion observations over space in Siberian boreal forests using field observations, satellite remote sensing imagery, and other geospatial data. The variance explained by our spatial model (overall *R* ^2^ = 0.51, adjusted *R* ^2^ = 0.47) was within the range of values obtained from previous efforts in North American boreal forests (Dieleman et al. 2020; Potter et al. 2023; Rogers et al. 2014; Veraverbeke et al. 2015, 2017; Walker, Rogers, et al. 2018). We did not find FWI System components to provide much predictive power. One potential explanation for why FWI does not appear to be significant in the spatial model could be the stronger importance of other geospatial variables, particularly dNBR and soil properties. The dNBR, a remotely sensed fire severity proxy, may be influenced by fire weather. As seen in previous upscaling efforts in boreal North America (Dieleman et al. 2020; Potter et al. 2023; Rogers et al. 2014; Veraverbeke et al. 2015; Walker, Rogers, et al. 2018), these variables have proven to be strong explanatory factors. Because of these reasons, the relative importance of FWI may be smaller in our spatial model. The distribution of fire‐wide combustion values from our spatial model closely matched the distribution from field observations (Table S11), supporting the validity of our site selection and its value in establishing typical emission rates for the region. Negative combustion values were observed around water streams in the Batamay fire scar, likely due to particularly high slope values along steep stream banks. While these localized features were not captured in our field plots, they do not reflect broader landscape patterns and are unlikely to significantly impact overall model performance. The relationships between geospatial variables and ecosystem properties influencing combustion have been shown to vary significantly across boreal regions. These differences can be effectively addressed by incorporating representative field observations. The strong agreement between observed and modeled combustion, along with the representativeness of the sampled gradients across the broader ecoregion (Figure S11), reinforces the reliability of our dataset for calibrating regional emission models and improving the accuracy of large‐scale C predictions. Our C stock and combustion estimates were incorporated into an updated field database that was used to calibrate fuel loads and consumption in the GFED 500‐m product (van Wees et al. 2022b). The global increase in fuel consumption compared to the previous model version (GFED4s) has mainly been attributed to higher organic soil C emissions from the boreal region as constrained by additional measurements (van Wees et al. 2022a). This underlines the importance of our new estimates and the need for additional field measurements of fuel loads and consumption in Siberia to better refine and advance models of fire‐induced C emissions. Forested peatlands, which store substantial amounts of soil organic C and promote deep belowground combustion, represent key ecosystems where additional in situ measurements would be particularly valuable (Turetsky et al. 2011).

## 5 Conclusions

This study aims to address persistent knowledge gaps associated with the impact of fires on the larch forests of northeastern Siberia. Our estimates of fuel consumption from stand‐replacing and surface fires averaged 3.20 kg C m^−2^, which is significantly higher than those previously reported for other Siberian forest ecosystems (Ivanova et al. 2011, 2020; Kukavskaya et al. 2013, 2017; McRae et al. 2006). We have shown that combustion rates in larch forests can be similar to those in North American boreal forests (Walker, Rogers, et al. 2020), with, on average, 78% of combusted C stemming from organic soils. C combustion was driven by both fire weather conditions and landscape variables. Our analysis provides new insights on combustion processes in Siberian larch forests, yet our dataset has a relatively small spatial footprint compared to the extent of these ecosystems. Our work urges for additional field‐based measurements across gradients of topography, fuel type and structure, soil conditions, fire weather, and fire severity. Such measurements are critical to calibrate and validate fire emission models, and thus to estimate the role of contemporary and future fires on the C balance of larch forest ecosystems in northeastern Siberia.

## Author Contributions

**Clement J. F. Delcourt:** conceptualization, formal analysis, investigation, methodology, visualization, writing – original draft, writing – review and editing. **Brendan M. Rogers:** investigation, methodology, resources, writing – review and editing. **Linar Akhmetzyanov:** investigation, writing – review and editing. **Brian Izbicki:** investigation, writing – review and editing. **Rebecca C. Scholten:** investigation, writing – review and editing. **Tatiana A. Shestakova:** investigation, writing – review and editing. **Dave van Wees:** investigation, writing – review and editing. **Michelle C. Mack:** methodology, resources, writing – review and editing. **Ute Sass‐Klaassen:** resources, writing – review and editing. **Sander Veraverbeke:** conceptualization, formal analysis, funding acquisition, investigation, methodology, visualization, writing – original draft, writing – review and editing.

## Conflicts of Interest

The authors declare no conflicts of interest.

## Supporting information


Data S1.

## Data Availability

The data that support the findings of this study are openly available in Zenodo at https://doi.org/10.5281/zenodo.10840088 and https://doi.org/10.5281/zenodo.6670869. The Sentinel‐2A MSI surface reflectance data were obtained from the Copernicus Data Space Ecosystem at https://doi.org/10.5270/S2_‐znk9xsj. The Northern Eurasia Land Cover Map was retrieved from the Global Land Cover 2000 database developed by the Joint Research Centre of the European Commission and available at https://forobs.jrc.ec.europa.eu/glc2000/data. https://doi.org/10.5067/MODIS/MCD64A1.061. The Moderate Resolution Imaging Spectrometer (MODIS) burned area data and the Visible Infrared Imaging Radiometer Suite (VIIRS) active fire locations can be retrieved from the NASA EOSDIS Land Processes Distributed Active Archive Center at https://doi.org/10.5067/MODIS/MCD64A1.061 and https://doi.org/10.5067/VIIRS/VNP14IMG.002, respectively. Fire weather data were obtained from the National Aeronautics and Space Administration (NASA) Center for Climate Simulation (NCCS) data portal at https://portal.nccs.nasa.gov/datashare/GlobalFWI/. Elevation data were obtained from the Polar Geospatial Center (University of Maryland) via Harvard Dataverse at https://doi.org/10.7910/DVN/3VDC4W. The Landsat 8 OLI/TIRS surface reflectance data were obtained from U.S. Geological Survey Earth Resources Observation and Science (EROS) Center at https://doi.org/10.5066/P9OGBGM6 and can also be accessed via Google Earth Engine (GEE) at https://developers.google.com/earth‐engine/datasets/catalog/LANDSAT_LC08_C02_T1_L2. Tree cover data were obtained from the NASA EOSDIS Land Processes Distributed Active Archive Center at https://doi.org/10.5067/MEaSUREs/GFCC/GFCC30TC.003. Soil properties were obtained from the International Soil Reference and Information Centre (ISRIC) at https://doi.org/10.17027/isric‐soilgrids.713396f4‐1687‐11ea‐a7c0‐a0481ca9e724 and can also be accessed at https://soilgrids.org/.

## References

[gcb70247-bib-0001] Abaimov, A. P. , J. A. Lesinski , O. Martinsson , and L. I. Milyutin . 1997. Variability and Ecology of Siberian Larch Species. Report 43, 123. Swedish University of Agricultural Sciences (SLU), Department of Silviculture.

[gcb70247-bib-0002] Abatzoglou, J. T. , A. P. Williams , L. Boschetti , M. Zubkova , and C. A. Kolden . 2018. “Global Patterns of Interannual Climate‐Fire Relationships.” Global Change Biology 24, no. 11: 5164–5175. 10.1111/gcb.14405. 30047195 PMC7134822

[gcb70247-bib-0003] Alexander, H. D. , M. C. Mack , S. Goetz , et al. 2012. “Carbon Accumulation Patterns During Post‐Fire Succession in Cajander Larch (*Larix cajanderi*) Forests of Siberia.” Ecosystems 15: 1065–1082. 10.1007/s10021-012-9567-6.

[gcb70247-bib-0004] Amiro, B. D. , A. Cantin , M. D. Flannigan , and W. J. De Groot . 2009. “Future Emissions From Canadian Boreal Forest Fires.” Canadian Journal of Forest Research 39, no. 2: 383–395. 10.1139/X08-154.

[gcb70247-bib-0005] Amiro, B. D. , A. L. Orchansky , A. G. Barr , et al. 2006. “The Effect of Post‐Fire Stand Age on the Boreal Forest Energy Balance.” Agricultural and Forest Meteorology 140: 41–50. 10.1016/j.agrformet.2006.02.014.

[gcb70247-bib-0006] Bartalev, S. A. , A. S. Belward , D. V. Erchov , and A. S. Isaev . 2003. “A New SPOT4‐VEGETATION Derived Land Cover Map of Northern Eurasia.” International Journal of Remote Sensing 24, no. 9: 1977–1982. 10.1080/0143116031000066297.

[gcb70247-bib-0007] Boby, L. A. , E. A. G. Schuur , M. C. Mack , D. Verbyla , and J. F. Johnstone . 2010. “Quantifying Fire Severity, Carbon, and Nitrogen Emissions in Alaska's Boreal Forest.” Ecological Applications 20, no. 6: 1633–1647. 10.1890/08-2295.1. 20945764

[gcb70247-bib-0009] Bowman, D. M. J. S. , J. K. Balch , P. Artaxo , et al. 2009. “Fire in the Earth System.” Science 324, no. 5926: 481–484. 10.1126/science.1163886. 19390038

[gcb70247-bib-0010] Brown, C. D. , and J. F. Johnstone . 2011. “How Does Increased Fire Frequency Affect Carbon Loss From Fire? A Case Study in the Northern Boreal Forest.” International Journal of Wildland Fire 20, no. 7: 829–837. 10.1071/WF10113.

[gcb70247-bib-0011] Brown, J. K. 1971. “A Planar Intersect Method for Sampling Fuel Volume and Surface Area.” Forest Science 17, no. 1: 96–102.

[gcb70247-bib-0012] Brown, J. K. , R. D. Oberheu , and C. M. Johnston . 1982. Handbook for Inventorying Surface Fuels as Biomass in the Interior West. General Technical Report INT‐GTR‐129. U.S. Department of Agriculture, Forest Service, Intermountain Forest and Range Experimental Station. 10.2737/INT-GTR-129.

[gcb70247-bib-0013] Brown, J. K. , and P. J. Roussopoulos . 1974. “Eliminating Biases in the Planar Intersect Method for Estimating Volumes of Small Fuels.” Forest Science 20, no. 4: 350–356.

[gcb70247-bib-0014] Burrell, A. L. , Q. Sun , R. Baxter , et al. 2022. “Climate Change, Fire Return Intervals and the Growing Risk of Permanent Forest Loss in Boreal Eurasia.” Science of the Total Environment 831: 154885. 10.1016/j.scitotenv.2022.154885. 35358519

[gcb70247-bib-0015] Chevychelov, A. P. , and N. P. Bosikov . 2010. “Natural Conditions.” In The Far North. Plant Vegetation, edited by E. I. Troeva , A. P. Isaev , M. M. Cherosov , and N. S. Karpov , vol. 3, 1–23. Springer. 10.1007/978-90-481-3774-9_1.

[gcb70247-bib-0016] de Groot, W. J. , A. S. Cantin , M. D. Flannigan , A. J. Soja , L. M. Gowman , and A. Newbery . 2013. “A Comparison of Canadian and Russian Boreal Forest Fire Regimes.” Forest Ecology and Management 294: 23–34. 10.1016/j.foreco.2012.07.033.

[gcb70247-bib-0017] de Groot, W. J. , J. M. Pritchard , and T. J. Lynham . 2009. “Forest Floor Fuel Consumption and Carbon Emissions in Canadian Boreal Forest Fires.” Canadian Journal of Forest Research 39, no. 2: 367–382. 10.1139/X08-192.

[gcb70247-bib-0018] De Santis, A. , and E. Chuvieco . 2009. “GeoCBI: A Modified Version of the Composite Burn Index for the Initial Assessment of the Short‐Term Burn Severity From Remotely Sensed Data.” Remote Sensing of Environment 113, no. 3: 554–562. 10.1016/j.rse.2008.10.011.

[gcb70247-bib-0019] Delcourt, C. J. F. , A. Combee , B. Izbicki , et al. 2021. “Evaluating the Differenced Normalized Burn Ratio for Assessing Fire Severity Using Sentinel‐2 Imagery in Northeast Siberian Larch Forests.” Remote Sensing 13, no. 12: 2311. 10.3390/rs13122311.

[gcb70247-bib-0020] Delcourt, C. J. F. , and S. Veraverbeke . 2022. “Allometric Equations and Wood Density Parameters for Estimating Aboveground and Woody Debris Biomass in Cajander Larch (*Larix cajanderi*) Forests of Northeast Siberia.” Biogeosciences 19, no. 18: 4499–4520. 10.5194/bg-19-4499-2022.

[gcb70247-bib-0021] Dieleman, C. M. , B. M. Rogers , S. Potter , et al. 2020. “Wildfire Combustion and Carbon Stocks in the Southern Canadian Boreal Forest: Implications for a Warming World.” Global Change Biology 26, no. 11: 6062–6079. 10.1111/gcb.15158. 32529727

[gcb70247-bib-0022] Eckdahl, J. A. , J. A. Kristensen , and D. B. Metcalfe . 2022. “Climatic Variation Drives Loss and Restructuring of Carbon and Nitrogen in Boreal Forest Wildfire.” Biogeosciences 19, no. 9: 2487–2506. 10.5194/bg-19-2487-2022.

[gcb70247-bib-0023] Eckdahl, J. A. , J. A. Kristensen , and D. B. Metcalfe . 2024. “Restricted Plant Diversity Limits Carbon Recapture After Wildfire in Warming Boreal Forests.” Communications Earth & Environment 5: 186. 10.1038/s43247-024-01333-7.

[gcb70247-bib-0024] Fan, L. , J. P. Wigneron , P. Ciais , et al. 2022. “Siberian Carbon Sink Reduced by Forest Disturbances.” Nature Geoscience 16, no. 1: 56–62. 10.1038/s41561-022-01087-x.

[gcb70247-bib-0025] Fedorov, A. N. , N. F. Vasilyev , Y. I. Torgovkin , et al. 2018. “Permafrost‐Landscape Map of the Republic of Sakha (Yakutia) on a Scale 1:1,500,000.” Geosciences 8, no. 12: 465. 10.3390/geosciences8120465.

[gcb70247-bib-0026] Field, R. D. , A. C. Spessa , N. A. Aziz , et al. 2015. “Development of a Global Fire Weather Database.” Natural Hazards and Earth System Sciences 15, no. 6: 1407–1423. 10.5194/nhess-15-1407-2015.

[gcb70247-bib-0027] Flannigan, M. , A. S. Cantin , W. J. de Groot , M. Wotton , A. Newbery , and L. M. Gowman . 2013. “Global Wildland Fire Season Severity in the 21st Century.” Forest Ecology and Management 294: 54–61. 10.1016/j.foreco.2012.10.022.

[gcb70247-bib-0028] Flannigan, M. , K. A. Logan , B. D. Amiro , W. R. Skinner , and B. J. Stocks . 2005. “Future Area Burned in Canada.” Climatic Change 72: 1–16. 10.1007/s10584-005-5935-y.

[gcb70247-bib-0029] Flannigan, M. , B. Stocks , M. Turetsky , and M. Wotton . 2009. “Impacts of Climate Change on Fire Activity and Fire Management in the Circumboreal Forest.” Global Change Biology 15, no. 3: 549–560. 10.1111/j.1365-2486.2008.01660.x.

[gcb70247-bib-0030] French, N. H. F. , W. J. de Groot , L. K. Jenkins , et al. 2011. “Model Comparisons for Estimating Carbon Emissions From North American Wildland Fire.” Journal of Geophysical Research: Biogeosciences 116: G00K05. 10.1029/2010JG001469.

[gcb70247-bib-0031] Genet, H. , A. D. McGuire , K. Barrett , et al. 2013. “Modeling the Effects of Fire Severity and Climate Warming on Active Layer Thickness and Soil Carbon Storage of Black Spruce Forests Across the Landscape in Interior Alaska.” Environmental Research Letters 8, no. 4: 045016. 10.1088/1748-9326/8/4/045016.

[gcb70247-bib-0032] Gibson, C. M. , L. E. Chasmer , D. K. Thompson , W. L. Quinton , M. D. Flannigan , and D. Olefeldt . 2018. “Wildfire as a Major Driver of Recent Permafrost Thaw in Boreal Peatlands.” Nature Communications 9: 3041. 10.1038/s41467-018-05457-1. PMC6072743 30072751

[gcb70247-bib-0033] Giglio, L. , L. Boschetti , D. P. Roy , M. L. Humber , and C. O. Justice . 2018. “The Collection 6 MODIS Burned Area Mapping Algorithm and Product.” Remote Sensing of Environment 217: 72–85. 10.1016/j.rse.2018.08.005. 30220740 PMC6136150

[gcb70247-bib-0034] Goodale, C. L. , M. J. Apps , R. A. Birdsey , et al. 2002. “Forest Carbon Sinks in the Northern Hemisphere.” Ecological Applications 12: 891–899. 10.2307/3060997.

[gcb70247-bib-0035] Granath, G. , C. D. Evans , J. Strengbom , et al. 2021. “The Impact of Wildfire on Biogeochemical Fluxes and Water Quality in Boreal Catchments.” Biogeosciences 18, no. 10: 3243–3261. 10.5194/bg-18-3243-2021.

[gcb70247-bib-0036] Gurney, K. R. , R. M. Law , A. S. Denning , et al. 2002. “Towards Robust Regional Estimates of CO_2_ Sources and Sinks Using Atmospheric Transport Models.” Nature 415: 626–630. 10.1038/415626a. 11832942

[gcb70247-bib-0037] Gustafsson, L. , M. Berglind , A. Granström , et al. 2019. “Rapid Ecological Response and Intensified Knowledge Accumulation Following a North European Mega‐Fire.” Scandinavian Journal of Forest Research 34, no. 4: 234–253. 10.1080/02827581.2019.1603323.

[gcb70247-bib-0038] Hanes, C. C. , X. Wang , P. Jain , M. A. Parisien , J. M. Little , and M. D. Flannigan . 2019. “Fire‐Regime Changes in Canada Over the Last Half Century.” Canadian Journal of Forest Research 49, no. 3: 256–269. 10.1139/cjfr-2018-0293.

[gcb70247-bib-0039] Harden, J. W. , S. E. Trumbore , B. J. Stocks , et al. 2000. “The Role of Fire in the Boreal Carbon Budget.” Global Change Biology 6: 174–184. 10.1046/j.1365-2486.2000.06019.x. 35026928

[gcb70247-bib-0040] Hobbie, S. E. , J. P. Schimel , S. E. Trumbore , and J. R. Randerson . 2000. “Controls Over Carbon Storage and Turnover in High‐Latitude Soils.” Global Change Biology 6: 196–210. 35026936 10.1046/j.1365-2486.2000.06021.x

[gcb70247-bib-0041] Hoy, E. E. , M. R. Turetsky , and E. S. Kasischke . 2016. “More Frequent Burning Increases Vulnerability of Alaskan Boreal Black Spruce Forests.” Environmental Research Letters 11, no. 9: 095001. 10.1088/1748-9326/11/9/095001.

[gcb70247-bib-0042] Ivanova, G. A. , S. G. Conard , E. A. Kukavskaya , and D. J. McRae . 2011. “Fire Impact on Carbon Storage in Light Conifer Forests of the Lower Angara Region, Siberia.” Environmental Research Letters 6, no. 4: 045203. 10.1088/1748-9326/6/4/045203.

[gcb70247-bib-0043] Ivanova, G. A. , E. A. Kukavskaya , V. A. Ivanov , S. G. Conard , and D. J. McRae . 2020. “Fuel Characteristics, Loads and Consumption in Scots Pine Forests of Central Siberia.” Journal of Forestry Research 31: 2507–2524. 10.1007/s11676-019-01038-0.

[gcb70247-bib-0044] Jain, P. , Q. E. Barber , S. Taylor , et al. 2024. Canada Under Fire–Drivers and Impacts of the Record‐Breaking 2023 Wildfire Season. ESS Open Archive. 10.22541/essoar.170914412.27504349/v1. PMC11335882 39164286

[gcb70247-bib-0045] Johnstone, J. F. , F. S. Chapin , T. N. Hollingsworth , M. C. Mack , V. Romanovsky , and M. Turetsky . 2010. “Fire, Climate Change, and Forest Resilience in Interior Alaska.” Canadian Journal of Forest Research 40, no. 7: 1302–1312. 10.1139/X10-061.

[gcb70247-bib-0046] Jones, M. W. , J. T. Abatzoglou , S. Veraverbeke , et al. 2022. “Global and Regional Trends and Drivers of Fire Under Climate Change.” Reviews of Geophysics 60, no. 3: e2020RG000726. 10.1029/2020RG000726.

[gcb70247-bib-0047] Jones, M. W. , S. Veraverbeke , N. Andela , et al. 2024. “Global Rise in Forest Fire Emissions Linked to Climate Change in the Extratropics.” Science 386: eadl5889. 10.1126/science.adl5889. 39418381

[gcb70247-bib-0048] Kane, E. S. , E. S. Kasischke , D. W. Valentine , M. R. Turetsky , and A. D. McGuire . 2007. “Topographic Influences on Wildfire Consumption of Soil Organic Carbon in Interior Alaska: Implications for Black Carbon Accumulation.” Journal of Geophysical Research: Biogeosciences 112: G03017. 10.1029/2007JG000458.

[gcb70247-bib-0049] Kasischke, E. S. , N. L. Christensen , and B. J. Stocks . 1995. “Fire, Global Warming, and the Carbon Balance of Boreal Forests.” Ecological Applications 5, no. 2: 437–451. 10.2307/1942034.

[gcb70247-bib-0050] Kasischke, E. S. , and E. E. Hoy . 2012. “Controls on Carbon Consumption During Alaskan Wildland Fires.” Global Change Biology 18, no. 2: 685–699. 10.1111/j.1365-2486.2011.02573.x.

[gcb70247-bib-0051] Kasischke, E. S. , E. J. Hyer , P. C. Novelli , et al. 2005. “Influences of Boreal Fire Emissions on Northern Hemisphere Atmospheric Carbon and Carbon Monoxide.” Global Biogeochemical Cycles 19, no. 1: GB1012. 10.1029/2004GB002300.

[gcb70247-bib-0052] Kelly, J. , T. S. Ibáñez , C. Santín , et al. 2021. “Boreal Forest Soil Carbon Fluxes One Year After a Wildfire: Effects of Burn Severity and Management.” Global Change Biology 27, no. 17: 4181–4195. 10.1111/gcb.15721. 34028945

[gcb70247-bib-0053] Kelly, J. , N. Kljun , Z. Cai , et al. 2024. “Wildfire Impacts on the Carbon Budget of a Managed Nordic Boreal Forest.” Agricultural and Forest Meteorology 351: 110016. 10.1016/j.agrformet.2024.110016.

[gcb70247-bib-0054] Kharuk, V. I. , E. I. Ponomarev , G. A. Ivanova , M. L. Dvinskaya , S. C. P. Coogan , and M. D. Flannigan . 2021. “Wildfires in the Siberian Taiga.” Ambio 50: 1953–1974. 10.1007/s13280-020-01490-x. 33512668 PMC8497666

[gcb70247-bib-0055] Köster, E. , K. Köster , F. Berninger , et al. 2018. “Changes in Fluxes of Carbon Dioxide and Methane Caused by Fire in Siberian Boreal Forest With Continuous Permafrost.” Journal of Environmental Management 228: 405–415. 10.1016/j.jenvman.2018.09.051. 30243076

[gcb70247-bib-0056] Krylov, A. , J. L. McCarty , P. Potapov , et al. 2014. “Remote Sensing Estimates of Stand‐Replacement Fires in Russia, 2002‐2011.” Environmental Research Letters 9, no. 10: 105007. 10.1088/1748-9326/9/10/105007.

[gcb70247-bib-0057] Kukavskaya, E. A. , L. V. Buryak , G. A. Ivanova , et al. 2013. “Influence of Logging on the Effects of Wildfire in Siberia.” Environmental Research Letters 8, no. 4: 045034. 10.1088/1748-9326/8/4/045034.

[gcb70247-bib-0058] Kukavskaya, E. A. , L. V. Buryak , O. P. Kalenskaya , and D. S. Zarubin . 2017. “Transformation of the Ground Cover After Surface Fires and Estimation of Pyrogenic Carbon Emissions in the Dark‐Coniferous Forests of Central Siberia.” Contemporary Problems of Ecology 10: 62–70. 10.1134/S1995425517010073.

[gcb70247-bib-0059] Kukavskaya, E. A. , E. G. Shvetsov , L. V. Buryak , P. D. Tretyakov , and P. Y. Groisman . 2023. “Increasing Fuel Loads, Fire Hazard, and Carbon Emissions From Fires in Central Siberia.” Fire 6, no. 2: 63. 10.3390/fire6020063.

[gcb70247-bib-0060] Li, Y. , T. A. J. Janssen , R. Chen , B. He , and S. Veraverbeke . 2024. “Trends and Drivers of Arctic‐Boreal Fire Intensity Between 2003 and 2022.” Science of the Total Environment 926: 172020. 10.1016/j.scitotenv.2024.172020. 38547987

[gcb70247-bib-0061] Mangiafico, S. S. 2025. “rcompanion: Functions to Support Extension Education Program Evaluation.” R Package Version 2.5.0. Accessed April 28, 2025. https://CRAN.R‐project.org/package=rcompanion/.

[gcb70247-bib-0062] Manies, K. L. , J. W. Harden , S. R. Silva , P. H. Briggs , and B. M. Schmid . 2004. Soil Data From Picea mariana Stands Near Delta Junction, Alaska of Different Ages and Soil Drainage Type. Open‐File Report 2004–1271. U.S. Department of the Interior, U.S. Geological Survey.

[gcb70247-bib-0063] Mazerolle, M. J. 2023. “AICcmodavg: Model Selection and Multimodel Inference Based on (Q)AIC(c).” R Package Version 2.3.3. Accessed April 28, 2025. https://cran.r‐project.org/package=AICcmodavg.

[gcb70247-bib-0064] McRae, D. J. , S. G. Conard , G. A. Ivanova , et al. 2006. “Variability of Fire Behavior, Fire Effects, and Emissions in Scotch Pine Forests of Central Siberia.” Mitigation and Adaptation Strategies for Global Change 11: 45–74. 10.1007/s11027-006-1008-4.

[gcb70247-bib-0065] O'Donnell, J. A. , J. W. Harden , A. D. McGuire , and V. E. Romanovsky . 2011. “Exploring the Sensitivity of Soil Carbon Dynamics to Climate Change, Fire Disturbance and Permafrost Thaw in a Black Spruce Ecosystem.” Biogeosciences 8, no. 5: 1367–1382. 10.5194/bg-8-1367-2011.

[gcb70247-bib-0066] O'Halloran, T. L. , B. E. Law , M. L. Goulden , et al. 2012. “Radiative Forcing of Natural Forest Disturbances.” Global Change Biology 18, no. 2: 555–565. 10.1111/j.1365-2486.2011.02577.x.

[gcb70247-bib-0067] Pan, Y. , R. A. Birdsey , J. Fang , et al. 2011. “A Large and Persistent Carbon Sink in the World's Forests.” Science 333, no. 6045: 988–993. 10.1126/science.1201609. 21764754

[gcb70247-bib-0068] Pan, Y. , R. A. Birdsey , O. L. Phillips , et al. 2024. “The Enduring World Forest Carbon Sink.” Nature 631: 563–569. 10.1038/s41586-024-07602-x. 39020035

[gcb70247-bib-0069] Parisien, M. A. , S. A. Parks , C. Miller , M. A. Krawchuk , M. Heathcott , and M. A. Moritz . 2011. “Contributions of Ignitions, Fuels, and Weather to the Spatial Patterns of Burn Probability of a Boreal Landscape.” Ecosystems 14: 1141–1155. 10.1007/s10021-011-9474-2.

[gcb70247-bib-0070] Ponomarev, E. I. , V. I. Kharuk , and K. J. Ranson . 2016. “Wildfires Dynamics in Siberian Larch Forests.” Forests 7, no. 6: 125. 10.3390/f7060125.

[gcb70247-bib-0071] Ponomarev, E. I. , N. Yakimov , T. Ponomareva , O. Yakubailik , and S. G. Conard . 2021. “Current Trend of Carbon Emissions From Wildfires in Siberia.” Atmosphere 12, no. 5: 559. 10.3390/atmos12050559.

[gcb70247-bib-0072] Porter, C. , I. Howat , M.‐J. Noh , et al. 2023. “ArcticDEM ‐ Mosaics, Version 4.1 [Dataset].” In ArcticDEM (Version V1). Harvard Dataverse. 10.7910/DVN/3VDC4W.

[gcb70247-bib-0073] Potter, S. , S. Cooperdock , S. Veraverbeke , et al. 2023. “Burned Area and Carbon Emissions Across Northwestern Boreal North America From 2001–2019.” Biogeosciences 20: 2785–2804. 10.5194/bg-20-2785-2023.

[gcb70247-bib-0074] Potter, S. , K. Solvik , A. Erb , et al. 2020. “Climate Change Decreases the Cooling Effect From Postfire Albedo in Boreal North America.” Global Change Biology 26, no. 3: 1592–1607. 10.1111/gcb.14888. 31658411

[gcb70247-bib-0075] Pyne, S. J. 1984. Introduction to Wildland Fire: Fire Management in the United States. John Wiley & Sons.

[gcb70247-bib-0076] R Core Team . 2024. R: A Language and Environment for Statistical Computing. R Foundation for Statistical Computing. Accessed April 5, 2025. https://www.R‐project.org/.

[gcb70247-bib-0077] Randerson, J. T. , H. Liu , M. G. Flanner , et al. 2006. “The Impact of Boreal Forest Fire on Climate Warming.” Science 314, no. 5802: 1130–1132. 10.1126/science.1132075. 17110574

[gcb70247-bib-0078] Rogers, B. M. , J. T. Randerson , and G. B. Bonan . 2013. “High‐Latitude Cooling Associated With Landscape Changes From North American Boreal Forest Fires.” Biogeosciences 10, no. 2: 699–718. 10.5194/bg-10-699-2013.

[gcb70247-bib-0079] Rogers, B. M. , A. J. Soja , M. L. Goulden , and J. T. Randerson . 2015. “Influence of Tree Species on Continental Differences in Boreal Fires and Climate Feedbacks.” Nature Geoscience 8: 228–234. 10.1038/ngeo2352.

[gcb70247-bib-0080] Rogers, B. M. , S. Veraverbeke , G. Azzari , et al. 2014. “Quantifying Fire‐Wide Carbon Emissions in Interior Alaska Using Field Measurements and Landsat Imagery.” Journal of Geophysical Research: Biogeosciences 119, no. 8: 1608–1629. 10.1002/2014JG002657.

[gcb70247-bib-0081] Schepaschenko, D. , A. Shvidenko , V. Usoltsev , et al. 2017. “A Dataset of Forest Biomass Structure for Eurasia.” Scientific Data 4: 170070. 10.1038/sdata.2017.70. 28509911 PMC5433390

[gcb70247-bib-0082] Scholten, R. , S. Veraverbeke , Y. Chen , and J. Randerson . 2024. Spatial Variability in Arctic‐Boreal Pyroregions Shaped by Climate and Human Influence. Research Square. 10.21203/rs.3.rs-3932189/v1. PMC11387193 39267694

[gcb70247-bib-0083] Scholten, R. C. , D. Coumou , F. Luo , and S. Veraverbeke . 2022. “Early Snowmelt and Polar Jet Dynamics Co‐Influence Recent Extreme Siberian Fire Seasons.” Science 378, no. 6623: 1005–1009. 10.1126/science.abn4419. 36454839

[gcb70247-bib-0084] Schroeder, W. , P. Oliva , L. Giglio , and I. A. Csiszar . 2014. “The New VIIRS 375m Active Fire Detection Data Product: Algorithm Description and Initial Assessment.” Remote Sensing of Environment 143: 85–96. 10.1016/j.rse.2013.12.008.

[gcb70247-bib-0085] Schuur, E. A. G. , J. Bockheim , J. G. Canadell , et al. 2008. “Vulnerability of Permafrost Carbon to Climate Change: Implications for the Global Carbon Cycle.” Bioscience 58, no. 8: 701–714. 10.1641/B580807.

[gcb70247-bib-0086] Sedano, F. , and J. T. Randerson . 2014. “Multi‐Scale Influence of Vapor Pressure Deficit on Fire Ignition and Spread in Boreal Forest Ecosystems.” Biogeosciences 11, no. 14: 3739–3755. 10.5194/bg-11-3739-2014.

[gcb70247-bib-0087] Seiler, W. , and P. J. Crutzen . 1980. “Estimates of Gross and Net Fluxes of Carbon Between the Biosphere and the Atmosphere From Biomass Burning.” Climatic Change 2: 207–247. 10.1007/BF00137988.

[gcb70247-bib-0088] Soja, A. J. , N. M. Tchebakova , N. H. F. French , et al. 2007. “Climate‐Induced Boreal Forest Change: Predictions Versus Current Observations.” Global and Planetary Change: 563274296. 10.1016/j.gloplacha.2006.07.028.

[gcb70247-bib-0089] Stocks, B. J. , T. J. Lynham , B. D. Lawson , et al. 1989. “The Canadian Forest Fire Danger Rating System: An Overview.” Forestry Chronicle 65, no. 6: 450–457. 10.5558/tfc65450-6.

[gcb70247-bib-0090] Stocks, B. J. , B. M. Wotton , M. D. Flannigan , M. A. Fosberg , D. R. Cahoon , and J. G. Goldammer . 2001. “Boreal Forest Fire Regimes and Climate Change.” In Remote Sensing and Climate Modeling: Synergies and Limitations. Advances in Global Change Research, edited by M. Beniston and M. M. Verstraete , vol. 7, 233–246. Springer. 10.1007/0-306-48149-9_10.

[gcb70247-bib-0091] Turetsky, M. R. , E. S. Kane , J. W. Harden , et al. 2011. “Recent Acceleration of Biomass Burning and Carbon Losses in Alaskan Forests and Peatlands.” Nature Geoscience 4: 27–31. 10.1038/ngeo1027.

[gcb70247-bib-0092] Ueyama, M. , H. Iwata , H. Nagano , N. Tahara , C. Iwama , and Y. Harazono . 2019. “Carbon Dioxide Balance in Early‐Successional Forests After Forest Fires in Interior Alaska.” Agricultural and Forest Meteorology 275: 196–207. 10.1016/j.agrformet.2019.05.020.

[gcb70247-bib-0093] van der Werf, G. R. , J. T. Randerson , L. Giglio , et al. 2017. “Global Fire Emissions Estimates During 1997–2016.” Earth System Science Data 9, no. 2: 697–720. 10.5194/essd-9-697-2017.

[gcb70247-bib-0094] van Leeuwen, T. T. , G. R. van Der Werf , A. A. Hoffmann , et al. 2014. “Biomass Burning Fuel Consumption Rates: A Field Measurement Database.” Biogeosciences 11, no. 24: 7305–7329. 10.5194/bg-11-7305-2014.

[gcb70247-bib-0095] Van Wagner, C. E. 1968. “The Line Intersect Method in Forest Fuel Sampling.” Forest Science 14, no. 1: 20–26.

[gcb70247-bib-0096] Van Wagner, C. E. 1982. Practical Aspects of the Line Intersect Method. Information Report PI‐X‐12E. Canadian Forestry Service, Maritimes Forest Research Centre.

[gcb70247-bib-0097] Van Wagner, C. E. 1987. Development and Structure of the Canadian Forest Fire Weather Index System. Forestry Technical Report 35. Canadian Forestry Service, Headquarters.

[gcb70247-bib-0098] van Wees, D. , G. R. van der Werf , J. T. Randerson , et al. 2022a. “Global Biomass Burning Fuel Consumption and Emissions at 500 m Spatial Resolution Based on the Global Fire Emissions Database (GFED).” Geoscientific Model Development 15: 8411–8437. 10.5194/gmd-15-8411-2022.

[gcb70247-bib-0099] van Wees, D. , G. R. van der Werf , J. T. Randerson , et al. 2022b. Field Data Synthesis Accompanying “Global Biomass Burning Fuel Consumption and Emissions at 500‐m Spatial Resolution Based on the Global Fire Emissions Database (GFED)”. Zenodo. 10.5281/zenodo.6670869.

[gcb70247-bib-0100] Veraverbeke, S. , C. J. F. Delcourt , E. Kukavskaya , et al. 2021. “Direct and Longer‐Term Carbon Emissions From Arctic‐Boreal Fires: A Short Review of Recent Advances.” Current Opinion in Environmental Science and Health 23: 100277. 10.1016/j.coesh.2021.100277.

[gcb70247-bib-0101] Veraverbeke, S. , B. M. Rogers , M. L. Goulden , et al. 2017. “Lightning as a Major Driver of Recent Large Fire Years in North American Boreal Forests.” Nature Climate Change 7: 529–534. 10.1038/nclimate3329.

[gcb70247-bib-0102] Veraverbeke, S. , B. M. Rogers , and J. T. Randerson . 2015. “Daily Burned Area and Carbon Emissions From Boreal Fires in Alaska.” Biogeosciences 12: 3579–3601. 10.5194/bg-12-3579-2015.

[gcb70247-bib-0103] Virkkala, A. M. , B. M. Rogers , J. D. Watts , et al. 2025. “Wildfires Offset the Increasing but Spatially Heterogeneous Arctic‐Boreal CO_2_ Uptake.” Nature Climate Change 15: 188–195. 10.1038/s41558-024-02234-5.

[gcb70247-bib-0104] Walker, X. J. , J. L. Baltzer , L. L. Bourgeau‐Chavez , et al. 2020. ABoVE: Synthesis of Burned and Unburned Forest Site Data, AK and Canada, 1983–2016. ORNL DAAC. 10.3334/ORNLDAAC/1744.

[gcb70247-bib-0105] Walker, X. J. , J. L. Baltzer , S. G. Cumming , et al. 2019. “Increasing Wildfires Threaten Historic Carbon Sink of Boreal Forest Soils.” Nature 572: 520–523. 10.1038/s41586-019-1474-y. 31435055

[gcb70247-bib-0106] Walker, X. J. , J. L. Baltzer , S. G. Cumming , et al. 2018. “Soil Organic Layer Consumption in Boreal Black Spruce and Jack Pine Stands of the Northwest Territories, Canada.” International Journal of Wildland Fire 27, no. 2: 125–134. 10.1071/WF17095.

[gcb70247-bib-0107] Walker, X. J. , B. M. Rogers , J. L. Baltzer , et al. 2018. “Cross‐Scale Controls on Carbon Emissions From Boreal Forest Megafires.” Global Change Biology 24, no. 9: 4251–4265. 10.1111/gcb.14287. 29697169

[gcb70247-bib-0108] Walker, X. J. , B. M. Rogers , S. Veraverbeke , et al. 2020. “Fuel Availability Not Fire Weather Controls Boreal Wildfire Severity and Carbon Emissions.” Nature Climate Change 10: 1130–1136. 10.1038/s41558-020-00920-8.

[gcb70247-bib-0109] Wang, J. A. , A. Baccini , M. Farina , J. T. Randerson , and M. A. Friedl . 2021. “Disturbance Suppresses the Aboveground Biomass Carbon Sink in North American Boreal Forests.” Nature Climate Change 11: 435–441. 10.1038/s41558-021-01027-4.

[gcb70247-bib-0110] Warren, W. , and P. Olsen . 1964. “A Line Intersect Technique for Assessing Logging Waste.” Forest Science 10, no. 3: 267–276.

[gcb70247-bib-0111] Webb, E. E. , H. D. Alexander , A. K. Paulson , et al. 2024. “Fire‐Induced Carbon Loss and Tree Mortality in Siberian Larch Forests.” Geophysical Research Letters 51, no. 1: e2023GL105216. 10.1029/2023GL105216.

[gcb70247-bib-0112] Whitman, E. , M.‐A. Parisien , D. K. Thompson , R. J. Hall , R. S. Skakun , and M. D. Flannigan . 2018. “Variability and Drivers of Burn Severity in the Northwestern Canadian Boreal Forest.” Ecosphere 9, no. 2: e02128. 10.1002/ecs2.2128.

[gcb70247-bib-0113] Wirth, C. 2005. “Fire Regime and Tree Diversity in Boreal Forests: Implications for the Carbon Cycle.” In Forest Diversity and Function. Ecological Studies, edited by M. Scherer‐Lorenzen , C. Körner , and E. D. Schulze , vol. 176, 309–344. Springer. 10.1007/3-540-26599-6_15.

[gcb70247-bib-0114] Wotton, B. M. 2009. “Interpreting and Using Outputs From the Canadian Forest Fire Danger Rating System in Research Applications.” Environmental and Ecological Statistics 16: 107–131. 10.1007/s10651-007-0084-2.

[gcb70247-bib-0115] Young, A. M. , P. E. Higuera , P. A. Duffy , and F. S. Hu . 2017. “Climatic Thresholds Shape Northern High‐Latitude Fire Regimes and Imply Vulnerability to Future Climate Change.” Ecography 40, no. 5: 606–617. 10.1111/ecog.02205.

[gcb70247-bib-0116] Zheng, B. , P. Ciais , F. Chevallier , E. Chuvieco , Y. Chen , and H. Yang . 2021. “Increasing Forest Fire Emissions Despite the Decline in Global Burned Area.” Science Advances 7: eabh2646. 10.1126/sciadv.abh2646. 34559570 PMC8462883

[gcb70247-bib-0117] Zheng, B. , P. Ciais , F. Chevallier , et al. 2023. “Record‐High CO_2_ Emissions From Boreal Fires in 2021.” Science 379, no. 6635: 912–917. 10.1126/science.ade0805. 36862792

