# Supplementary material for: Carbon Emissions From Fires in Eastern Siberian Larch Forests
Source: Glob Chang Biol. 2025 May 23;31(5):e70247. doi: 10.1111/gcb.70247 (PMC12100588; doi:10.1111/gcb.70247)
Supplement: Supplementary file 1 — Data S1. [file GCB-31-e70247-s001.docx]

# Supplementary Methods

## Stand age determination

To determine stand age, we collected cores or disks from five trees of the dominant cohort within each plot. Two cores were extracted per tree using an increment borer and stored for further processing. Samples were prepared and analysed using standard dendrochronological techniques. Tree cores were air-dried and glued onto wooden mounts. We then used a core-microtome to cut plane surfaces on the cores that were polished using a series of succesively finer sand-paper grits until tree rings were clearly discernible. We used high-resolution scans of the cores and CooRecorder (Cybis Elektronik & Data AB) software to enumerate tree rings. Basal disks were sanded and analysed using LINTAB measuring stage and WinTSAP (Rinntech) software.

## Estimation of pre-fire loads of fine and coarse woody debris

We used the line-intersect method to estimate pre-fire loads of all dead and down woody materials on the ground within each plot. For fine woody debris (FWD, ≤ 7 cm in diameter), this involved counting the number of pieces intersecting the transect line per diameter size class. Fuel load for any size class 𝑖 was then computed as follows (Brown, 1974; Delcourt & Veraverbeke 2022; Nalder et al., 1999; Van Wagner, 1982):

$\text{FWD}_{i}=\frac{\pi^{2}\times G_{i}\times\sec h\times n_{i}\times\text{MSD}_{i}\times s}{8\times L}$ (S1)

where FWD is the fine woody debris load (t ha^-1^), 𝐺 is the specific gravity (g cm^-3^), $\sec h$ is the tilt correction factor, 𝑛 is the number of intercepts over the transect line, MSD is the mean squared diameter (cm^2^), 𝐿 is the length of the transect line (m), and 𝑠 is the slope correction factor given by:

$s=\sqrt{1+\text{tan}^{2}\alpha}$ (S2)

where 𝛼 is the ground slope angle (degrees). FWD biomass was estimated for *Larix cajanderi* and *Pinus sylvestris* using a tilt correction factor of 1.13 (Brown, 1974), and size-class-specific values of 𝐺 and MSD derived from measurements collected in our study areas (Table S3). We used the methodology described in Delcourt & Veraverbeke (2022) to derive wood density parameters for *P. sylvestris*. Biomass estimates were multiplied by size-class-specific values of FWD carbon (C) concentration to determine pre-fire C content (Table S3). For coarse woody debris (CWD, > 7 cm in diameter), we measured the diameter of every piece that intersected the transect line and recorded its condition according to the degree of decomposition using the five-class system described in Fogel et al. (1973) and Maser et al. (1979). CWD biomass for any decay class 𝑗 was calculated as follows:

$\text{CWD}_{j}=\frac{\pi^{2}\times{G^{c}}_{j}\times\sum_{j} d^{2}}{8\times L}$ (S3)

where CWD is the coarse woody debris C content (t C ha^-1^), 𝐺^𝐶^ is the wood C density (g C cm^-3^) and ∑_𝑗_𝑑^2^ is the sum of squared diameters (cm^2^). CWD biomass was retrieved using decay-class-specific C density values for *L. cajanderi* and *P. sylvestris* (Table S4).

## Spatial representativeness of field plots

We assessed whether our field plots were representative of the variability in fire size, fire duration, fire severity, and pre-fire stand and soil properties observed within the larger pyrogeographically-defined region. The region of interest was defined as the extratropical forest pyrome 2 (ExTropF2) (Jones et al., 2024), cropped to northeastern Siberia. Jones et al. (2024) used the *k*-means clustering algorithm to group 414 forest ecoregions of the world (Olson et al., 2001) into 12 forest pyromes sharing a similar set of correlations between burned area and 14 predictor variables associated with climatic, vegetation, and human controls. Our study area covers approximately 500 Mha. All fire perimeters within the region for 2017 and 2018, the years of our fire events, were retrieved from the Arctic-boreal fire atlas recently developed by Scholten et al. (2024), totaling 6824 fires. Fire size and duration were extracted from the fire perimeter attributes. We assessed fire severity using the differenced Normalized Burn Ratio (dNBR) derived from Landsat-8 imagery in Google Earth Engine. The dNBR was calculated using the mean Normalized Burn Ratio (NBR) between June 1 and August 31 for both pre-fire and post-fire years. We also analyzed tree cover for pre-fire year 2015 (Sexton et al., 2013) and soil properties, including soil organic carbon, bulk density, pH, percent of sand, silt, and clay integrated over the top 30 cm of soil, from the latest release of SoilGrids250m (Hengl et al., 2017). Distributions of pixel values were compared with the variability observed in our field plots to assess spatial representativeness (Figure S11).

# Supplementary Tables

[**Table S1.** Species-specific allometric equations used to estimate pre-fire biomass associated with each tree component 4](#_Toc197105774)

[**Table S2**. Allometric equations relating diameter at breast height (DBH) to stem, stem wood, stem bark, branches, foliage, and aboveground tree biomass at Scots pine (*Pinus sylvestris*) sites 6](#_Toc197105775)

[**Table S3.** Mean squared diameter (MSD) and mean (± standard deviation) specific gravity (G) of fine dead and down woody debris (FWD) by diameter size class for Cajander larch (*Larix cajanderi*) and Scots pine (*Pinus sylvestris*) 7](#_Toc197105776)

[**Table S4.** Carbon (C) density of coarse woody debris (CWD) by decay class for Cajander larch (*Larix cajanderi*) and Scots pine (*Pinus sylvestris*) 8](#_Toc197105777)

[**Table S5.** Sources of error associated with our approach to quantifying carbon (C) combustion from vegetation, woody debris, and organic soils in burned plots 9](#_Toc197105778)

[**Table S6.** Mean site-level coefficients of variation from averaging species-specific allometric equations to retrieve pre-fire tree biomass 10](#_Toc197105779)

[**Table S7**. Coefficients of determination (*R*^2^) based on linear models between carbon (C) combustion metrics and hypothesized drivers 11](#_Toc197105780)

[**Table S8.** Spatial predictors used for spatial scaling of total carbon combustion 12](#_Toc197105781)

[**Table S9.** Summary of burned plot characteristics including pre-fire stand structure, carbon (C) pools, fire severity, and combustion 13](#_Toc197105782)

[**Table S10.** Results of AICc-based model selection assessing the relationships between response variables of relative total, aboveground, and belowground carbon (C) combustion, to a set of simple linear regression 14](#_Toc197105783)

[**Table S11.** Comparison of minimum, mean, maximum, and standard deviation of (C) combustion between the field plots and the spatial model across Batamay and Yert fire scars 16](#_Toc197105784)

**Table S1.** Species-specific allometric equations used to estimate pre-fire biomass associated with each tree component. All equations are of the form Y = a × D^b^ where Y is in units of dry weight and D is either the diameter at breast height (DBH) or the basal diameter (D_0_). Tree components include stem wood (SW), stem bark (SB), total stem (wood and bark, ST), branches (wood and bark, BR), foliage (FL), and total aboveground biomass (AB). Allometric equations were developed for Pinus sylvestris based on measurements of 41 trees retrieved from Schepaschenko et al. (2017) using the same approach as in Delcourt & Veraverbeke (2022). See Figure S2 and Table S2 for further details about the development of these equations. To retrieve pre-fire tree biomass when the diameter measured in the field differed from that used in the corresponding allometric equations, we derived DBH-D_0_ relationships for Larix cajanderi, Pinus sylvestris, and Salix spp. from in situ measurements (Figure S3). The conversion factor from D_0_ to DBH was set to 0.90 for Betula pendula and Alnus spp.

| ^*^Mäkelä & Vanninen (1998) provided two separate equations for sapwood and heartwood biomass that were summed up to compute stem wood biomass from DBH. ^†^Smith et al. (2014) provided two separate equations for live and dead branches biomass that were summed up to compute branches biomass from DBH. ^‡^Berner et al. (2015) provided allometric equations relating shrub basal diameter to new growth, which include leaves and new twigs. | *Salix* spp. | | *Alnus* spp. | | *Betula pendula* | *Pinus sylvestris* | | *Larix cajanderi* | | | **Species** |
| --- | --- | --- | --- | --- | --- | --- | --- | --- | --- | --- | --- |
|  | D_0_ | DBH | D_0_ | D_0_ | DBH | DBH | DBH | DBH | DBH | DBH | **Diameter** |
|  | ST, BR, FL^‡^, AB | ST, BR, FL  ST, BR, FL, AB  SW, SB, FL, AB | ST, BR, FL^‡^, AB | ST, BR, FL | SW, SB, BR^†^, FL, AB | ST, SW, SB, BR, FL, AB | SW^*^, SB, BR, FL, AB | SW, SB, BR, FL, AB | ST, BR, FL, AB | ST, BR, FL | **Tree component (*Y*)** |
|  | Berner et al. (2015) | Ter-Mikaelian & Korzukhin (1997) | Berner et al. (2015) | Alexander, Mack, Goetz, Beck, et al. (2012) | Smith et al. (2014) | This study | Mäkelä & Vanninen (1998) | Delcourt & Veraverbeke (2022) | Alexander, Mack, Goetz, Loranty, et al. (2012) | Kajimoto et al. (2006) | **References** |
|  | Cherskii, Sakha Republic, Russia | Maine, USA  Maine, USA  Upper Great Lakes, USA | Cherskii, Sakha Republic, Russia | Bonanza Creek Long Term Ecological Research Site (64.86°N, 147.85°W), Alaska, USA | Norway | Krasnoyarsk krai (60.72°N, 89.13°E), Russia | Southern Finland | Magadan Oblast (60.50°N, 148.00°E) Ust-Yansky district (69.88°N, 135.59°E), Sakha Republic, Russia | Cherskii (68.74°N, 161.40°E),  Sakha Republic, Russia | Cherskii (69°N, 160°E),  Oymyakon (63°N, 145°E),  Sakha Republic, Russia | **Location** |
|  | Natural | Unknown  Unknown  Managed | Natural | Natural | Managed | Natural | Natural/Managed | Natural | Natural | Natural | **Type of stands** |
|  | 1 | 3 | 1 | 1 | 1 | 1 | 1 | 2 | 1 | 2 | **Number of equation sets** |

**Table S2**. Allometric equations relating diameter at breast height (DBH) to stem, stem wood, stem bark, branches, foliage, and aboveground tree biomass at Scots pine (Pinus sylvestris) sites. Tree measurements (n = 97) were selected from an experiment conducted in Krasnoyarsk krai, Russia (Wirth et al., 1999). We filtered out trees under 30 years old and over 200 years old (n = 41). Allometry is expressed as a power-form equation Y = a × DBH^b^ where units of Y are in kilograms of dry weight and DBH is in centimeters. The coefficients a and b were derived using weighted nonlinear least squares regressions in which residuals were weighted by DBH^-2c^ to correct for non-constant residuals. The value of the exponent c was determined for each biomass component by (1) visual assessment of the plot of the weighted residuals against the fitted values and (2) approximation of the conditional variance of biomass following Picard et al. (2012). For each allometry model, the range of DBH used in regression is provided, as well as the number of trees, the standard errors (SEs) of the coefficients, and the root mean square error (RMSE).

| **Component *Y* (kg)** | **DBH range (cm)** | **Number of trees** | ***c*** | ***a*** | ***a* [SE]** | ***b*** | ***b* [SE]** | **RMSE (kg)** |
| --- | --- | --- | --- | --- | --- | --- | --- | --- |
| Stem | 1.1–40.7 | 41 | 2.0 | 0.110 | 0.022 | 2.35 | 0.074 | 53.707 |
| Stem wood | 1.1–30.1 | 34 | 2.5 | 0.077 | 0.014 | 2.48 | 0.076 | 17.561 |
| Stem bark | 1.1–30.1 | 34 | 1.5 | 0.020 | 0.006 | 2.04 | 0.108 | 1.521 |
| Branches | 1.1–40.7 | 41 | 2.0 | 0.009 | 0.004 | 2.45 | 0.170 | 8.482 |
| Foliage | 1.1–40.7 | 41 | 2.0 | 0.020 | 0.005 | 1.95 | 0.116 | 3.400 |
| Aboveground | 1.1–40.7 | 41 | 2.5 | 0.144 | 0.015 | 2.30 | 0.048 | 45.144 |

**Table S3.** Mean squared diameter (MSD) and mean (± standard deviation) specific gravity (G) of fine dead and down woody debris (FWD) by diameter size class for Cajander larch (Larix cajanderi) and Scots pine (Pinus sylvestris). MSD and G values for L. cajanderi were taken from Delcourt & Veraverbeke (2022), and values for P. sylvestris were derived following the same approach as for L. cajanderi. The number of pieces for each size class is reported in parentheses. Letters a and b represent significant differences (p-value < 0.05) between size classes as determined by the Tukey-Kramer test (specific gravity) and the Wilcoxon rank sum test (MSD) for multiple comparisons. To estimate biomass of size class I FWD, we used MSD, specific gravity, and carbon (C) concentration values from size class II when data were missing.

| **Diameter size class** | **MSD (cm^2^)** | ***G* (g cm^-3^)** | **C concentration** |
| --- | --- | --- | --- |
| *Larix cajanderi* |  |  |  |
| I (< 0.5 cm) | 0.042 (6) ^ab^ | Not measured |  |
| II (0.5–0.99 cm) | 0.668 (47) ^a^ | 0.672 ± 0.049 (30) ^a^ | 0.516 |
| III (1.0–2.99 cm) | 3.267 (123) ^b^ | 0.633 ± 0.066 (40) ^b^ | 0.515 |
| IV (3.0–4.99 cm) | 13.886 (32) ^b^ | 0.625 ± 0.052 (13) ^ab^ | 0.500 |
| V (5.0–6.99 cm) | 35.158 (15) ^ab^ | 0.659 ± 0.069 (12) ^ab^ | 0.491 |
| *Pinus sylvestris* |  |  |  |
| I (< 0.5 cm) | Not measured | Not measured |  |
| II (0.5–0.99 cm) | 0.611 (9) ^a^ | 0.669 ± 0.089 (11) ^a^ | 0.516 |
| III (1.0–2.99 cm) | 3.707 (18) ^a^ | 0.593 ± 0.052 (14) ^b^ | 0.515 |
| IV (3.0–4.99 cm) | 16.070 (8) ^a^ | 0.598 ± 0.060 (6) ^ab^ | 0.500 |
| V (5.0–6.99 cm) | 39.083 (6) ^a^ | 0.536 ± 0.060 (5) ^b^ | 0.491 |

*Source*: Carbon concentration values were taken from Currie & Nadelhoffer (2002) using measurements performed on sound woody debris collected from a pine stand (*Pinus resinosa* Ait.) of the Harvard Forest (Massachusetts, USA). Note that the size classes in the original study were slightly different (FS: 0.5–1.0 cm, FM: 1.0–2.5 cm, FL: 2.5–5.0 cm, FX: 5.0–10.0 cm).

**Table S4.** Carbon (C) density of coarse woody debris (CWD) by decay class for Cajander larch (Larix cajanderi) and Scots pine (Pinus sylvestris). The five-class system of CWD decomposition used in this study is described in Fogel et al. (1973) and Maser et al. (1979).

| **Decay class** | **Wood C density (g C cm^-3^)** | |
| --- | --- | --- |
|  | ***Larix cajanderi*** | ***Pinus sylvestris*** |
| I | 0.274 | 0.201 |
| II | 0.209 | 0.160 |
| III | 0.151 | 0.117 |
| IV | 0.113 | 0.071 |
| V | 0.071 | 0.065 |

Sources for parameter values: *Larix cajanderi* (Yang et al., 2018), *Pinus sylvestris* (Kankrina & Harmon, 1995)

**Table S5.** Sources of error associated with our approach to quantifying carbon (C) combustion from vegetation, woody debris, and organic soils in burned plots. An alternative approach was determined for each source and included in a series of simulations to quantify the uncertainty of our method using a Monte Carlo framework. σ: standard deviation; FWD: fine woody debris; MSD: mean squared diameter; G: wood specific gravity; CWD: coarse woody debris; SOL: soil organic layer.

| **Source** | **Main approach** | **Alternative approach** |
| --- | --- | --- |
| *Vegetation* |  |  |
| Pre-fire biomass of tree components | Averaged biomass from all applicable allometric equations | Varied randomly, applied systemically to every tree biomass component, tree, and plot. σ derived for each species and plot from separate equations (Table S6) |
| C concentration of tree components | Stem wood and bark: 47%, foliage: 46%, branches: 48% (Alexander, Mack, Goetz, Loranty, et al., 2012) | Varied randomly, applied systemically to every tree biomass component, tree, and plot. σ = 3% |
| Fire consumption of tree components | Visually assessed in the field using a 12-point scale (Figure S4) | Varied randomly, applied systemically to every tree biomass component, tree, and plot. σ = 5% for cones and needles, σ = 10% for branches, σ = 15% for stem bark and stem wood, σ = 20% when overall tree consumption was estimated |
| *Woody debris* |  |  |
| FWD loads parameters | Size-class specific MSD and *G* values for larch and pine trees (Table S3), determined from in situ measurements. Tilt correction factor: 1.13 (Brown, 1974) | Varied randomly, applied systemically to every species, size class, and plot. *G* values varied randomly by standard deviation (Table S3). σ = 10% for MSD values. σ = 5% for tilt correction factor |
| CWD loads parameters | Decay-class wood C density for larch and pine trees (Table S4) | Varied randomly, applied systemically to every species, decay class, and plot. σ = 20% |
| FWD C concentration | Used size-class specific values (Table S3) | Varied randomly, applied systemically to every species, size class, and plot. σ = 3% |
| Fire consumption of woody debris | Visually assessed in the field using a plot-level fractional consumption estimate | Varied randomly, applied systemically to every type of woody debris and plot. σ = 30% |
| *Belowground* |  |  |
| Reconstructing pre-fire SOL depth | Relationship between pre-fire SOL depth and adventitious root height above mineral soil derived from measurements in unburned plots | Varied randomly by standard errors of the slope and intercept, applied systemically to every soil core/profile and plot |
| Pre-fire bulk density and C concentration | Mean values per soil horizon derived from measurements in unburned plots (Table 1) | Varied randomly by standard deviation (Table 1), applied systemically to every forest type, soil horizon, and plot |
| Mean soil core combustion | Averaged combustion from five soil cores per plot | Varied randomly by standard error of the mean, applied systemically to every plot |
| Combustion at additional soil profiles | Estimated from burn depth using a relationship derived from soil cores (Figure S5) | Varied randomly by standard errors of the slope and intercept, applied systemically to every soil profile and plot |

**Table S6.** Mean site-level coefficients of variation from averaging species-specific allometric equations to retrieve pre-fire tree biomass.

| **Species** | **Number of plots** | **Coefficient of variation** | | | |
| --- | --- | --- | --- | --- | --- |
|  |  | **Stem wood** | **Stem bark** | **Branches** | **Foliage** |
| Larch dense |  |  |  |  |  |
| *Larix cajanderi* | 17 | 0.20 | 0.50 | 0.41 | 0.49 |
| *Pinus sylvestris* | 0 |  |  |  |  |
| *Betula pendula* | 16 | 0.20 | 0.46 | 0.40 | 0.49 |
| *Alnus* spp. | 4 | 0.18 | 0.18 | 0.22 | 0.49 |
| *Salix* spp. | 6 | 0.23 | 0.23 | 0.55 | 0.53 |
| All species | 17 | 0.20 | 0.45 | 0.41 | 0.49 |
| Larch open |  |  |  |  |  |
| *Larix cajanderi* | 20 | 0.27 | 0.48 | 0.42 | 0.48 |
| *Pinus sylvestris* | 0 |  |  |  |  |
| *Betula pendula* | 15 | 0.24 | 0.35 | 0.36 | 0.49 |
| *Alnus* spp. | 10 | 0.19 | 0.19 | 0.18 | 0.47 |
| *Salix* spp. | 7 | 0.24 | 0.24 | 0.58 | 0.54 |
| All species | 20 | 0.24 | 0.37 | 0.36 | 0.49 |
| Mixed |  |  |  |  |  |
| *Larix cajanderi* | 4 | 0.26 | 0.49 | 0.42 | 0.48 |
| *Pinus sylvestris* | 4 | 0.57 | 0.34 | 0.63 | 0.85 |
| *Betula pendula* | 3 | 0.40 | 0.43 | 0.52 | 0.67 |
| *Alnus* spp. | 0 |  |  |  |  |
| *Salix* spp. | 0 |  |  |  |  |
| All species | 4 | 0.44 | 0.41 | 0.55 | 0.71 |
| All sites |  |  |  |  |  |
| *Larix cajanderi* | 41 | 0.24 | 0.49 | 0.41 | 0.48 |
| *Pinus sylvestris* | 4 | 0.57 | 0.34 | 0.63 | 0.85 |
| *Betula pendula* | 34 | 0.24 | 0.41 | 0.39 | 0.51 |
| *Alnus* spp. | 14 | 0.18 | 0.18 | 0.19 | 0.48 |
| *Salix* spp. | 13 | 0.24 | 0.24 | 0.57 | 0.53 |
| All species | 41 | 0.24 | 0.41 | 0.40 | 0.51 |

**Table S7**. Coefficients of determination (R^2^) based on linear models between carbon (C) combustion metrics and hypothesized drivers. Ca: aboveground carbon combustion (kg C m^−2^); pCa: proportion of pre-fire aboveground carbon combusted (0–1); Cb: belowground carbon combustion (kg C m^−2^); pCb: proportion of pre-fire belowground carbon combusted (0–1); Ct: total carbon combustion (kg C m^−2^); pCt: proportion of total pre-fire carbon combusted (0–1). Shaded cells indicate significant relationships (p-value < 0.05), with light and dark grey indicating positive and negative relationships, respectively.

| **Set** | **Variables** | **Ca** | ***p*Ca** | **Cb** | ***p*Cb** | **Ct** | ***p*Ct** |
| --- | --- | --- | --- | --- | --- | --- | --- |
| Plot attributes | Latitude (degrees) | 0.065 | 0.018 | 0.189 | 0.013 | 0.235 | 0.006 |
|  | Elevation (m.a.s.l) | 0.139 | 0.078 | 0.225 | 0.011 | 0.330 | 0.041 |
|  | Slope (degrees) | 0.015 | 0.015 | 0.000 | 0.037 | 0.005 | 0.005 |
|  | Moisture class (xerix–subhygric) | 0.104 | 0.191 | 0.118 | 0.211 | 0.090 | 0.162 |
|  | Condensed moisture class (*n* = 3) | 0.023 | 0.083 | 0.072 | 0.171 | 0.068 | 0.058 |
|  | Stand age (years) | 0.018 | 0.061 | 0.359 | 0.011 | 0.145 | 0.002 |
|  | Pre-fire SOL depth (cm) | 0.036 | 0.013 | 0.520 | 0.101 | 0.435 | 0.001 |
| Pre-fire stand composition | Stand basal area (m^2^ ha^−1^) | 0.049 | 0.132 | 0.021 | 0.002 | 0.080 | 0.216 |
|  | Stand density (stems m^−2^) | 0.127 | 0.087 | 0.050 | 0.005 | 0.000 | 0.004 |
|  | Proportion of *Larix cajanderi* among vegetation (0–1) | 0.105 | 0.107 | 0.050 | 0.043 | 0.000 | 0.060 |
|  | Proportion of *Pinus sylvestris* among vegetation (0–1) | 0.005 | 0.022 | 0.050 | 0.044 | 0.015 | 0.022 |
|  | Proportion of *Betula pendula* among vegetation (0–1) | 0.102 | 0.116 | 0.096 | 0.005 | 0.007 | 0.037 |
|  | Proportion of *Salix* spp. among vegetation (0–1) | 0.085 | 0.031 | 0.201 | 0.092 | 0.254 | 0.088 |
|  | Proportion of *Alnus* spp. among vegetation (0–1) | 0.019 | 0.015 | 0.019 | 0.019 | 0.027 | 0.027 |
|  | Tree basal area (m^2^ ha^−1^) | 0.035 | 0.176 | 0.017 | 0.000 | 0.065 | 0.278 |
|  | Tree density (stems m^−2^) | 0.084 | 0.035 | 0.013 | 0.000 | 0.003 | 0.000 |
|  | Proportion of *Larix cajanderi* among tree species (0–1) | 0.094 | 0.178 | 0.229 | 0.008 | 0.044 | 0.062 |
|  | Proportion of *Pinus sylvestris* among tree species (0–1) | 0.005 | 0.022 | 0.050 | 0.044 | 0.015 | 0.022 |
|  | Proportion of *Betula pendula* among tree species (0–1) | 0.083 | 0.137 | 0.151 | 0.001 | 0.025 | 0.034 |
|  | Proportion of trees (0–1) | 0.002 | 0.000 | 0.018 | 0.002 | 0.020 | 0.000 |
|  | Pre-fire tree biomass (kg C m^−2^) | 0.035 | 0.189 | 0.049 | 0.009 | 0.105 | 0.322 |
|  | Pre-fire vegetation biomass (kg C m^−2^) | 0.038 | 0.182 | 0.049 | 0.009 | 0.107 | 0.315 |
|  | Pre-fire aboveground biomass (kg C m^−2^) | 0.072 | 0.139 | 0.033 | 0.017 | 0.119 | 0.327 |
| Fire attributes | Date of burn (Julian day) | 0.008 | 0.064 | 0.002 | 0.000 | 0.000 | 0.062 |
|  | Temperature (°C) | 0.000 | 0.025 | 0.054 | 0.056 | 0.035 | 0.018 |
|  | Relative humidity (%) | 0.006 | 0.012 | 0.130 | 0.083 | 0.062 | 0.006 |
|  | Precipitation (kg m^−2^) | 0.022 | 0.000 | 0.001 | 0.023 | 0.012 | 0.013 |
|  | Wind speed (m s^−1^) | 0.019 | 0.040 | 0.000 | 0.002 | 0.012 | 0.017 |
|  | Fine Fuel Moisture Code (FFMC) | 0.012 | 0.027 | 0.196 | 0.042 | 0.167 | 0.035 |
|  | Duff Moisture Code (DMC) | 0.110 | 0.122 | 0.091 | 0.055 | 0.172 | 0.046 |
|  | Drought Code (DC) | 0.063 | 0.010 | 0.135 | 0.022 | 0.189 | 0.000 |
|  | Initial Spread Index (ISI) | 0.001 | 0.000 | 0.238 | 0.039 | 0.125 | 0.007 |
|  | Buildup Index (BUI) | 0.104 | 0.062 | 0.146 | 0.049 | 0.229 | 0.015 |
|  | Fire Weather Index (FWI) | 0.012 | 0.009 | 0.306 | 0.071 | 0.233 | 0.012 |
|  | Daily Severity Ranking (DSR) | 0.002 | 0.001 | 0.298 | 0.074 | 0.194 | 0.004 |
|  | Vapor Pressure Deficit (VPD) | 0.018 | 0.005 | 0.131 | 0.111 | 0.043 | 0.001 |

**Table S8.** Spatial predictors used for spatial scaling of total carbon combustion. Variables that were retained in the final model are marked with a *. VIIRS: Visible Infrared Imaging Radiometer Suite; MERRA-2: Modern-Era Retrospective Analysis for Research and Application version 2.

| **Predictor set** | **Predictors** | **Spatial resolution (m)** | **Source** |
| --- | --- | --- | --- |
| Topographic | Elevation | 10 | Porter et al. (2023)  Arctic DEM Mosaics version 4.1  TWI calculated using QGIS 3.22 |
|  | Slope* |  |  |
|  | Aspect |  |  |
|  | Topographic Wetness Index (TWI) |  |  |
| Fire severity | differenced Normalized Burn Ratio (dNBR)* | 30 | Google Earth Engine Landsat 8 Level 2 Collection 2 Tier 1 Clouds masked with cfmask dNBR calculated using the mean NBR between June 1 and August 31 for both pre‑fire and post‑fire years.  *Batamay*: 31 pre‑fire images (2016) and 32 post‑fire images (2018)  *Yert*: 33 pre‑fire images (2017) and 36 post‑fire images (2019) |
| Burn timing | Date of burn | Closest VIIRS active fire detection | Schroeder et al. (2014)  VIIRS monthly global active fire product (VNP14IMGML) at 375 m resolution |
| Tree cover | Pre‑fire tree cover | 30 | Sexton et al. (2013)  Global Forest Cover Change (GFCC) tree cover (V003) for pre‑fire year 2015 |
| Fire weather | Fine Fuel Moisture Code (FFMC) | ~50,000 (0.5° × 2/3°) | Field et al. (2015)  Global Fire Weather Database (GFWED) version 2 using MERRA-2 reanalysis with bias-corrected precipitation |
|  | Duff Moisture Code (DMC) |  |  |
|  | Drought Code (DC) |  |  |
|  | Buildup Index (BUI) |  |  |
|  | Initital Spread Index (ISI) |  |  |
|  | Fire Weather Index (FWI) |  |  |
|  | Daily Severity Ranking (DSR) |  |  |
|  | Temperature |  |  |
|  | Precipitation |  |  |
|  | Wind speed |  |  |
|  | Relative humidity |  |  |
| Soil properties | Soil organic carbon | 250 | Hengl et al. (2017)  Latest release of SoilGrids250m (May 2020) |
|  | Bulk density |  |  |
|  | pH |  |  |
|  | % Sand* |  |  |
|  | % Silt |  |  |
|  | % Clay |  |  |
|  | Integrated over the top 15 cm, 30 cm, 100 cm |  |  |

**Table S9.** Summary of burned plot characteristics including pre-fire stand structure, carbon (C) pools, fire severity, and combustion. Mean ± standard deviation as well as full range (in parentheses) are given for each variable. SOL: soil organic layers.

| **Variables** | **Units** | **Larch dense** | **Larch open** | **Mixed larch/pine** | **All forest types** |
| --- | --- | --- | --- | --- | --- |
| Burned sites | number of burned sites | 17 | 20 | 4 | 41 |
| Burn date | day of the year | 188.6 | 188.6 | 191 | 188.8 |
| GeoCBI | 0–3 | 2.49 ± 0.54 (1.33–3.00) | 2.43 ± 0.51 (1.53–3.00) | 1.97 ± 0.47 (1.67–2.66) | 2.41 ± 0.53 (1.33–3.00) |
| Stand age | years | 55.3 ± 17.7 (9–67) | 112.2 ± 33.3 (63–214) | 131.3 ± 39.7 (86–160) | 89.5 ± 40.7 (9–214) |
| Pre-fire tree density | stems m^-2^ | 2.47 ± 1.42 (0.82–5.55) | 1.09 ± 1.46 (0.12–6.98) | 2.01 ± 1.04 (0.57–3.02) | 1.75 ± 1.53 (0.12–6.98) |
| Pre-fire tree basal area | m^2^ ha^-1^ | 19.69 ± 6.25 (5.98–31.13) | 22.70 ± 8.07 (10.58–37.52) | 22.60 ± 6.65 (13.79–28.07) | 21.44 ± 7.22 (5.98–37.52) |
| Cajander larch proportion | 0–1 | 0.80 ± 0.13 (0.51–1.00) | 0.65 ± 0.34 (0.09–1.00) | 0.29 ± 0.28 (0.06–0.65) | 0.68 ± 0.30 (0.06–1.00) |
| Pre-fire tree C pool | kg C m^-2^ | 2.44 ± 0.86 (0.82–4.50) | 3.05 ± 1.18 (1.46–5.43) | 4.07 ± 1.64 (2.03–5.99) | 2.90 ± 1.18 (0.82–5.99) |
| Pre-fire aboveground C pool | kg C m^-2^ | 2.72 ± 0.89 (1.17–4.54) | 3.18 ± 1.31 (1.47–6.48) | 4.43 ± 1.68 (2.20–6.27) | 3.12 ± 1.26 (1.17–6.48) |
| Moisture class | 1–6 | 3.29 ± 0.92 (2–5) | 2.60 ± 1.05 (1–5) | 2.25 ± 0.96 (1–3) | 2.85 ± 1.04 (1–5) |
| Pre-fire SOL depth | cm | 11.0 ± 3.1 (8.0–19.6) | 12.4 ± 2.5 (7.1–18.6) | 13.8 ± 1.7 (12.1–16.2) | 11.9 ± 2.8 (7.1–19.6) |
| Pre-fire belowground C pool | kg C m^-2^ | 2.97 ± 0.77 (2.35–5.28) | 3.66 ± 0.76 (2.10–5.54) | 4.92 ± 0.50 (4.46–5.62) | 3.50 ± 0.93 (2.10–5.62) |
| Total pre-fire C pool | kg C m^-2^ | 5.70 ± 1.17 (3.51–7.66) | 6.84 ± 1.51 (4.68–10.53) | 9.35 ± 1.87 (6.67–10.94) | 6.61 ± 1.74 (3.51–10.94) |
| Proportion of total pre-fire C from belowground | 0–1 | 0.53 ± 0.10 (0.38–0.74) | 0.55 ± 0.11 (0.37–0.76) | 0.54 ± 0.10 (0.43–0.67) | 0.54 ± 0.10 (0.37–0.76) |
| Burn depth | cm | 8.0 ± 1.8 (4.1–10.7) | 10.0 ± 1.6 (6.8–12.5) | 10.9 ± 0.7 (10.1–11.7) | 9.3 ± 1.9 (4.1–12.5) |
| Residual SOL depth | cm | 2.9 ± 2.3 (0.3–9.6) | 2.4 ± 1.8 (0.3–6.1) | 2.9 ± 1.3 (1.4–4.6) | 2.7 ± 1.9 (0.3–9.6) |
| Residual belowground C pool | kg C m^-2^ | 0.86 ± 0.64 (0.24–2.68) | 0.96 ± 0.52 (0.31–2.15) | 1.91 ± 0.31 (1.56–2.31) | 1.01 ± 0.63 (0.24–2.68) |
| Aboveground C combusted | kg C m^-2^ | 0.81 ± 0.39 (0.29–1.55) | 0.65 ± 0.46 (0.16–1.94) | 0.63 ± 0.45 (0.22–1.21) | 0.71 ± 0.43 (0.16–1.94) |
| Belowground C combusted | kg C m^-2^ | 2.11 ± 0.50 (0.99–2.81) | 2.70 ± 0.47 (1.79–3.43) | 3.02 ± 0.21 (2.83–3.31) | 2.49 ± 0.56 (0.99–3.43) |
| Total C combusted | kg C m^-2^ | 2.92 ± 0.74 (1.61–3.92) | 3.35 ± 0.75 (2.05–4.79) | 3.65 ± 0.34 (3.25–4.04) | 3.20 ± 0.75 (1.61–4.79) |
| Proportion of total pre-fire C combusted | 0–1 | 0.52 ± 0.12 (0.32–0.76) | 0.50 ± 0.13 (0.32–0.72) | 0.40 ± 0.06 (0.35–0.49) | 0.50 ± 0.12 (0.32–0.76) |
| Proportion of total C combusted from belowground | 0–1 | 0.73 ± 0.09 (0.58–0.89) | 0.82 ± 0.10 (0.59–0.95) | 0.83 ± 0.10 (0.70–0.94) | 0.78 ± 0.10 (0.58–0.95) |

**Table S10.** Results of AICc-based model selection assessing the relationships between response variables of relative total, aboveground, and belowground carbon (C) combustion, to a set of simple linear regression. Variables included in each model are listed in Table 2. For each model, the number of parameters (K), the sample size-corrected Akaike information criterion (AICc), the change in AICc relative to the best model (ΔAICc), the model weight (wi), the Log-Likelihood (LogL), and the marginal R^2^ are given. Bold indicates the most probable models based on ΔAICc < 2.

| **Relative belowground C combustion (*p*Cb)** | ***R*^2^** | 0.01 | 0.08 | 0.23 | 0.38 | **0** | **0.07** | 0.20 | **0.31** |
| --- | --- | --- | --- | --- | --- | --- | --- | --- | --- |
|  | **LogL** | 27.98 | 29.44 | 32.18 | 36.52 | **27.70** | **29.21** | 31.45 | **34.47** |
|  | **wi** | 0.03 | 0.03 | 0.01 | 0.08 | **0.22** | **0.31** | 0.06 | **0.27** |
|  | **ΔAICc** | 4.91 | 4.60 | 8.04 | 2.71 | **0.68** | **0** | 3.41 | **0.32** |
|  | **AICc** | −46.85 | −47.16 | −43.72 | −49.05 | **−51.08** | **−51.76** | −48.35 | **−51.44** |
|  |  |  |  |  |  |  |  |  |  |
| **Relative aboveground C combustion (*p*Ca)** | ***R*^2^** | **0.29** | **0.35** | **0.49** | 0.49 | 0 | 0.01 | 0.19 | 0.20 |
|  | **LogL** | **25.55** | **27.24** | **31.14** | 31.18 | 18.49 | 18.67 | 21.82 | 22.13 |
|  | **wi** | **0.29** | **0.42** | **0.24** | 0.05 | 0 | 0 | 0 | 0 |
|  | **ΔAICc** | **0.78** | **0** | **1.13** | 4.40 | 10.10 | 12.08 | 13.67 | 16.00 |
|  | **AICc** | **−41.99** | **−42.77** | **−41.64** | −38.37 | −32.67 | −30.69 | −29.10 | −26.77 |
|  |  |  |  |  |  |  |  |  |  |
| **Relative total C combustion (*p*Ct)** | ***R*^2^** | **0.33** | **0.36** | **0.51** | 0.53 | 0 | 0.01 | 0.09 | 0.09 |
|  | **LogL** | **37.42** | **38.28** | **42.58** | 43.38 | 29.11 | 29.35 | 30.26 | 30.27 |
|  | **wi** | **0.41** | **0.27** | **0.22** | 0.09 | 0 | 0 | 0 | 0 |
|  | **ΔAICc** | **0** | **0.88** | **1.22** | 2.97 | 11.82 | 13.67 | 19.75 | 22.70 |
|  | **AICc** | **−65.73** | **−64.85** | **−64.51** | −62.76 | −53.91 | −52.06 | −45.98 | −43.03 |
|  |  |  |  |  |  |  |  |  |  |
|  | **K** | 4 | 5 | 8 | 9 | 2 | 3 | 6 | 7 |
|  | **Model** | M2 | M6 | M4 | Full | Null | M3 | M1 | M5 |

**Table S11.** Comparison of minimum, mean, maximum, and standard deviation of (C) combustion between the field plots and the spatial model across Batamay and Yert fire scars.

| **Metric** | **Field plots** | **Spatial model** |
| --- | --- | --- |
| Minimum combustion (kg C m^−2^) | 1.61 | −4.26 |
| Mean combustion (kg C m^−2^) | 3.20 | 3.31 |
| Maximum combustion (kg C m^−2^) | 4.79 | 5.03 |
| Standard deviation of combustion (kg C m^−2^) | 0.75 | 0.65 |

# Supplementary Figures

[**Figure S1.** Forest types sampled within Batamay and Yert fire scars 18](#_Toc185710975)

[**Figure S2.** Allometry models developed for Scots pine (*Pinus sylvestris*) from measurements collected in the Krasnoyarsk krai, Russia 19](#_Toc185710976)

[**Figure S3**. Relationships between basal diameter (D_0_) and diameter at breast height (DBH) for (a) *Larix cajanderi*, (b) *Pinus sylvestris*, and (c) *Salix* spp 20](#_Toc185710977)

[**Figure S4.** Consumption scale used during field sampling to assess fire-induced fractional consumption of tree components 21](#_Toc185710978)

[**Figure S5.** Relationship between burn depth and soil organic carbon (C) combustion at burned cores with adventitious root measurements 22](#_Toc185710979)

[**Figure S6.** Attribution of uncertainty in (a) vegetation, (b) woody debris, and (c) belowground carbon (C) combustion estimates using a Monte Carlo framework 23](#_Toc185710980)

[**Figure S7.** Maps of geospatial predictors used in the final model to upscale combustion over Batamay (left) and Yert (right) fire scars 24](#_Toc185710981)

[**Figure S8.** Observed and predicted carbon (C) combustion at field plots from a multiple linear regression with inputs of slope, differenced Normalized Burn Ratio, and percent sand in the top 15 cm of soil 25](#_Toc185710982)

[**Figure S9.** Field pictures of young and dense larch-dominated stands 26](#_Toc185710983)

[**Figure S10.** Influence of stand age on (a) total pre-fire carbon (C) stock, (b) pre-fire soil organic layer (SOL) depth, and (c) burn depth 27](#_Toc185710984)

[**Figure S11.** Spatial representativeness of field plots 28](#_Toc185710985)


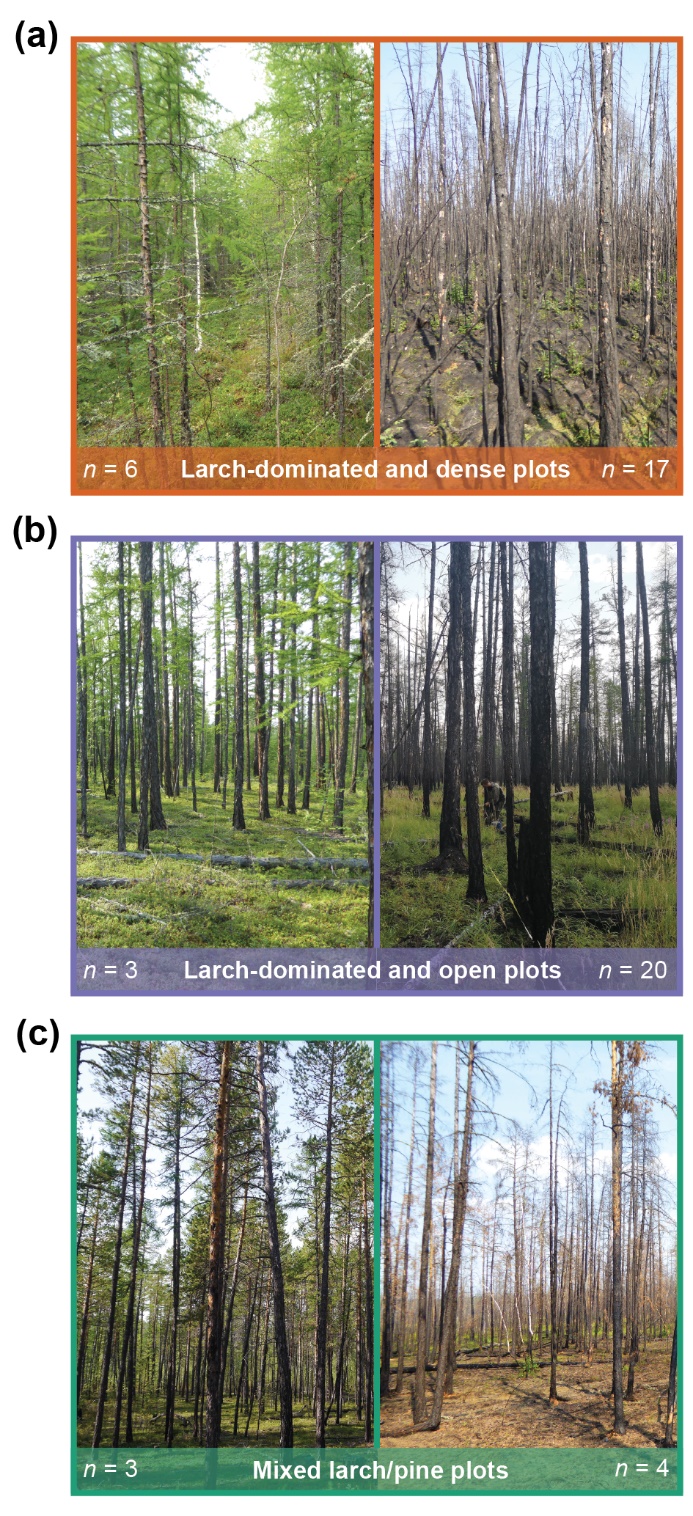


**Figure S1.** Forest types sampled within Batamay and Yert fire scars. (a) Larch-dominated, young, and dense stands; (b) larch-dominated, open, and mature stands; (c) mixed larch/pine, open, and mature stands. For each forest type, the left picture depicts an unburned stand, while the right picture shows a burned stand. The corresponding number of plots are given below each picture.


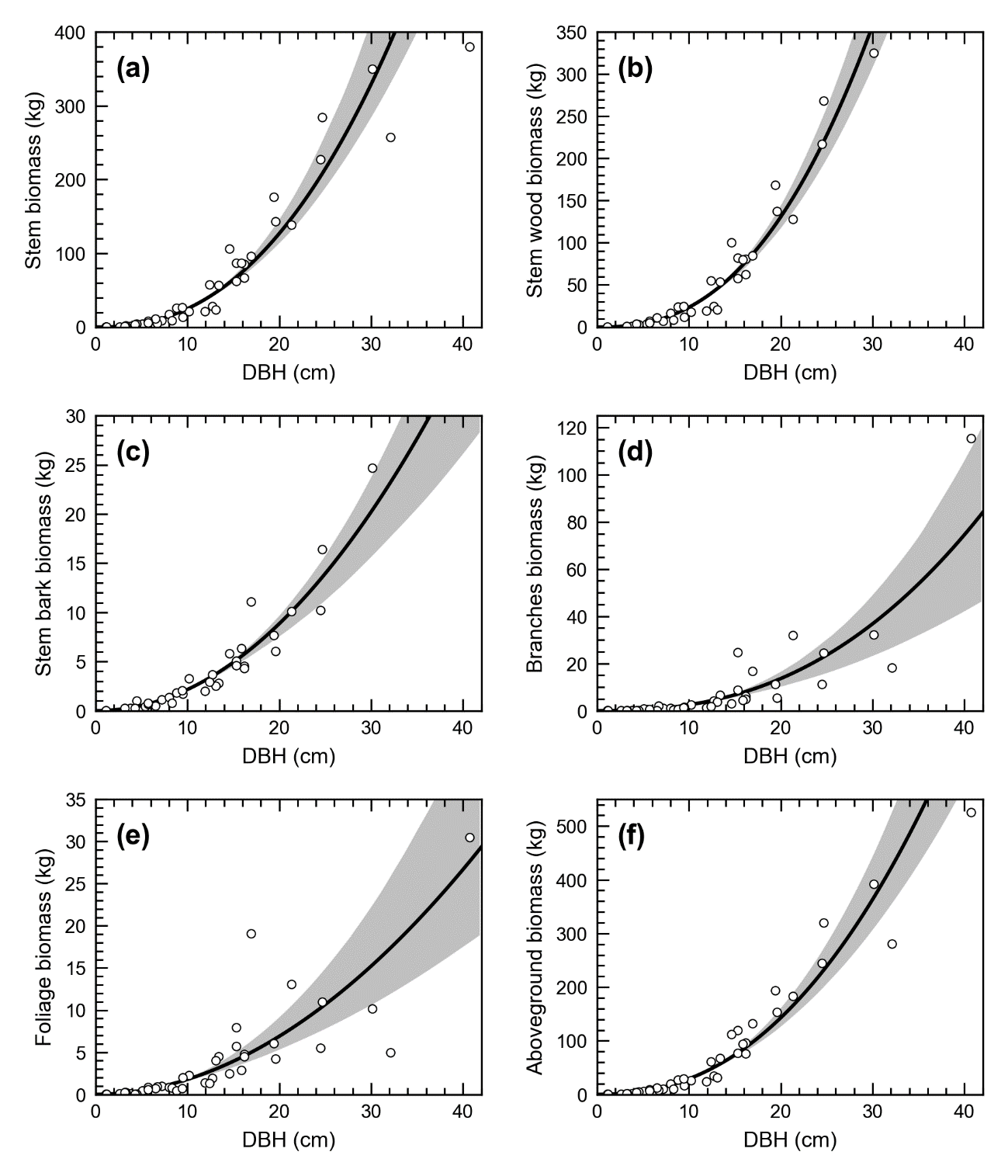


**Figure S2.** Allometry models developed for Scots pine (Pinus sylvestris) from measurements collected in the Krasnoyarsk krai, Russia (Wirth et al., 1999). The allometric relationships relate diameter at breast height (DBH) to (a) stem, (b) stem wood, (c) stem bark, (d) branches, (e) foliage, and (f) aboveground tree biomass. Regression coefficients are given in Table S2.


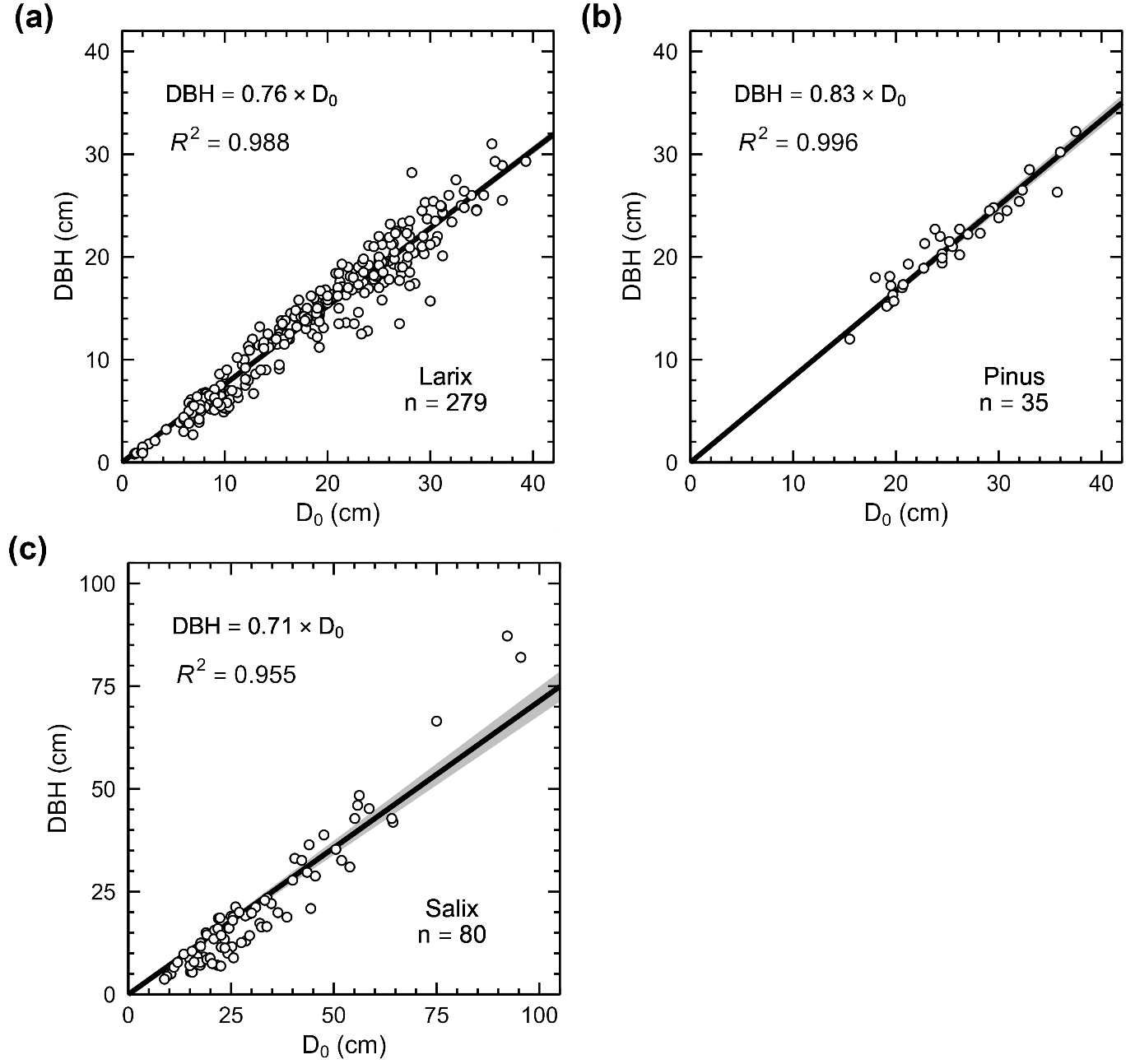


**Figure S3**. Relationships between basal diameter (D_0_) and diameter at breast height (DBH) for (a) Larix cajanderi, (b) Pinus sylvestris, and (c) Salix spp. Measurements on larch and pine trees were collected in both of our fire scars whereas measurements on willow trees were performed in disturbed and undisturbed forests at four sites around Fairbanks, Alaska.


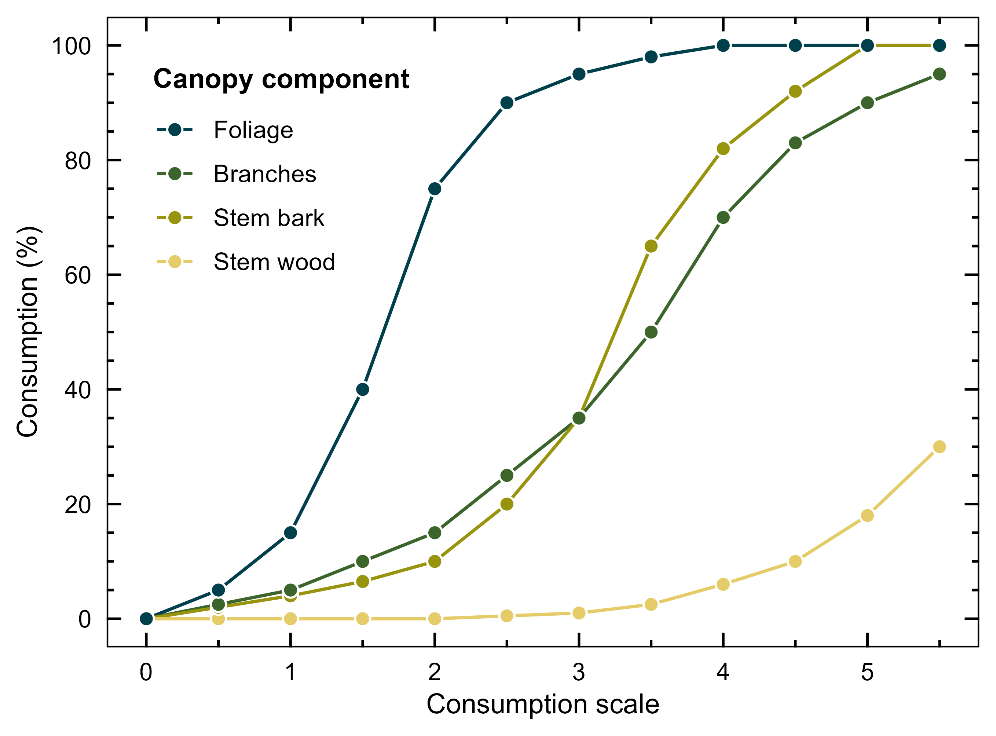


**Figure S4.** Consumption scale used during field sampling to assess fire-induced fractional consumption of tree components (modified from Dieleman et al., 2020).


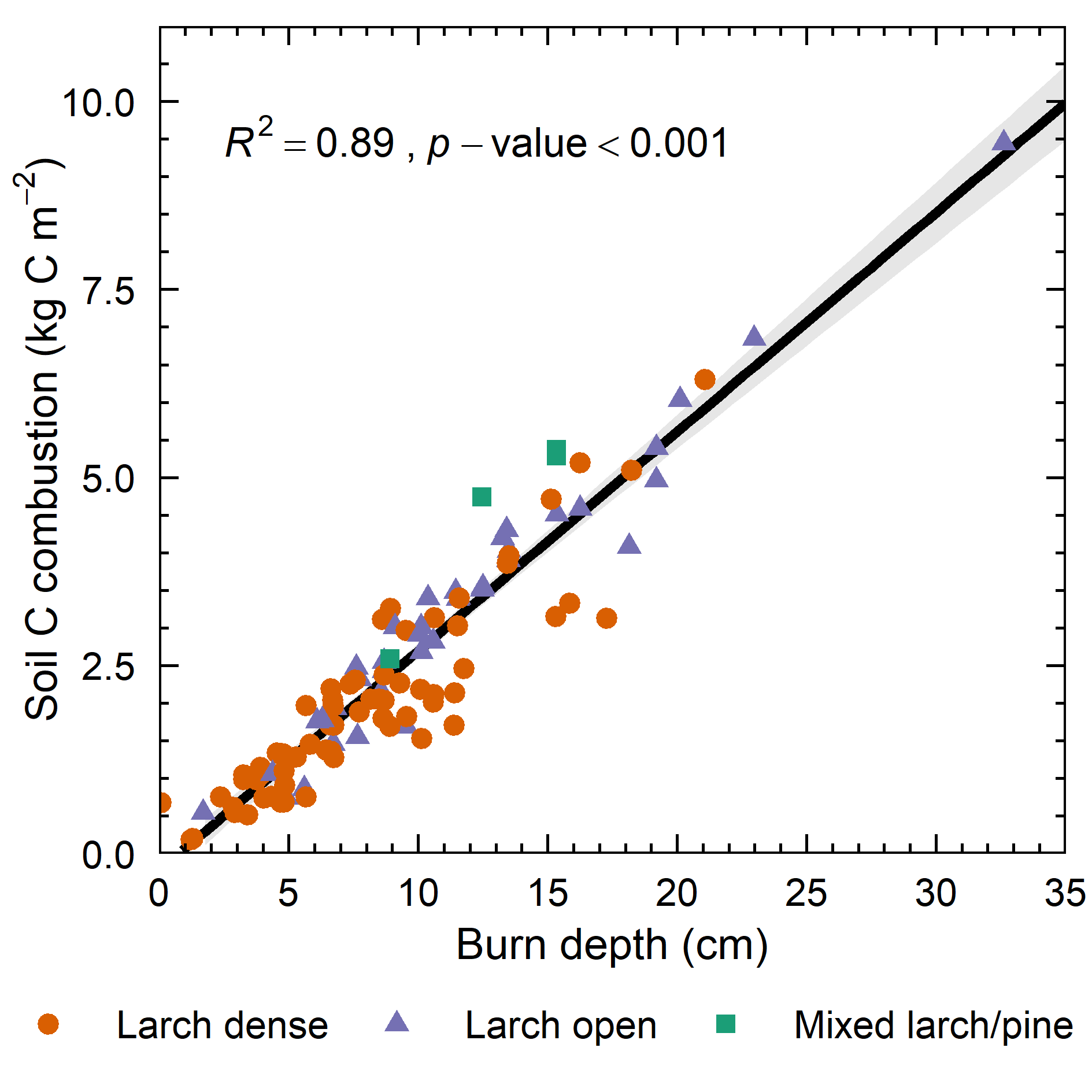


**Figure S5.** Relationship between burn depth and soil organic carbon (C) combustion at burned cores with adventitious root measurements.


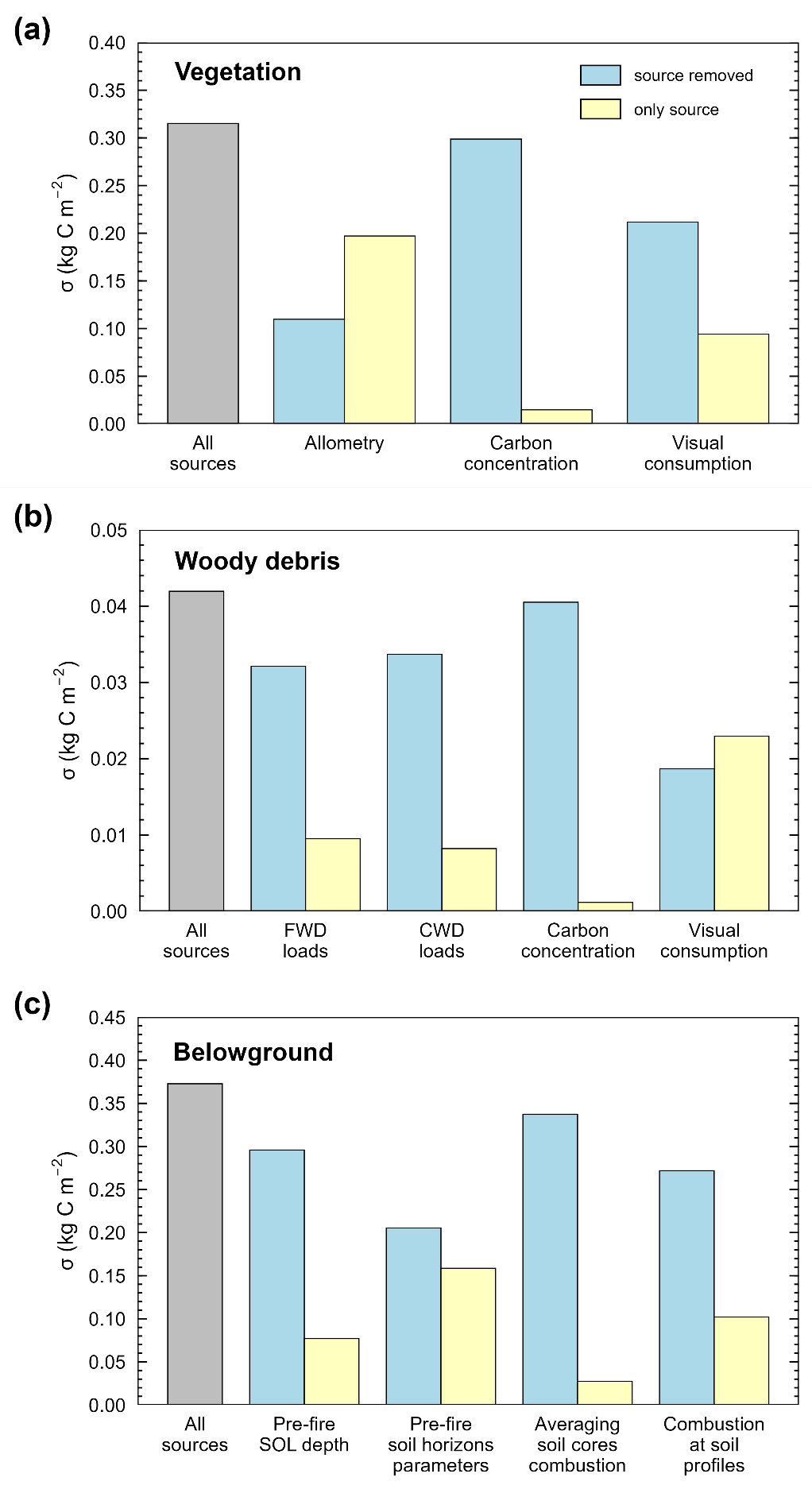


**Figure S6.** Attribution of uncertainty in (a) vegetation, (b) woody debris, and (c) belowground carbon (C) combustion estimates using a Monte Carlo framework. In the all-sources scenario (gray), uncertainty was introduced across all relevant parameters and methodological choices. For each source, two additional scenarios were simulated: ‘source removed’ (blue), in which the values and methods for the source remained constant as in the main approach, and ‘source only’ (yellow), in which the source exclusively influenced the outcome. The standard deviation (σ) of the combustion estimates from 1000 runs was calculated for each scenario. FWD: fine woody debris; CWD: coarse woody debris; SOL: soil organic layer.


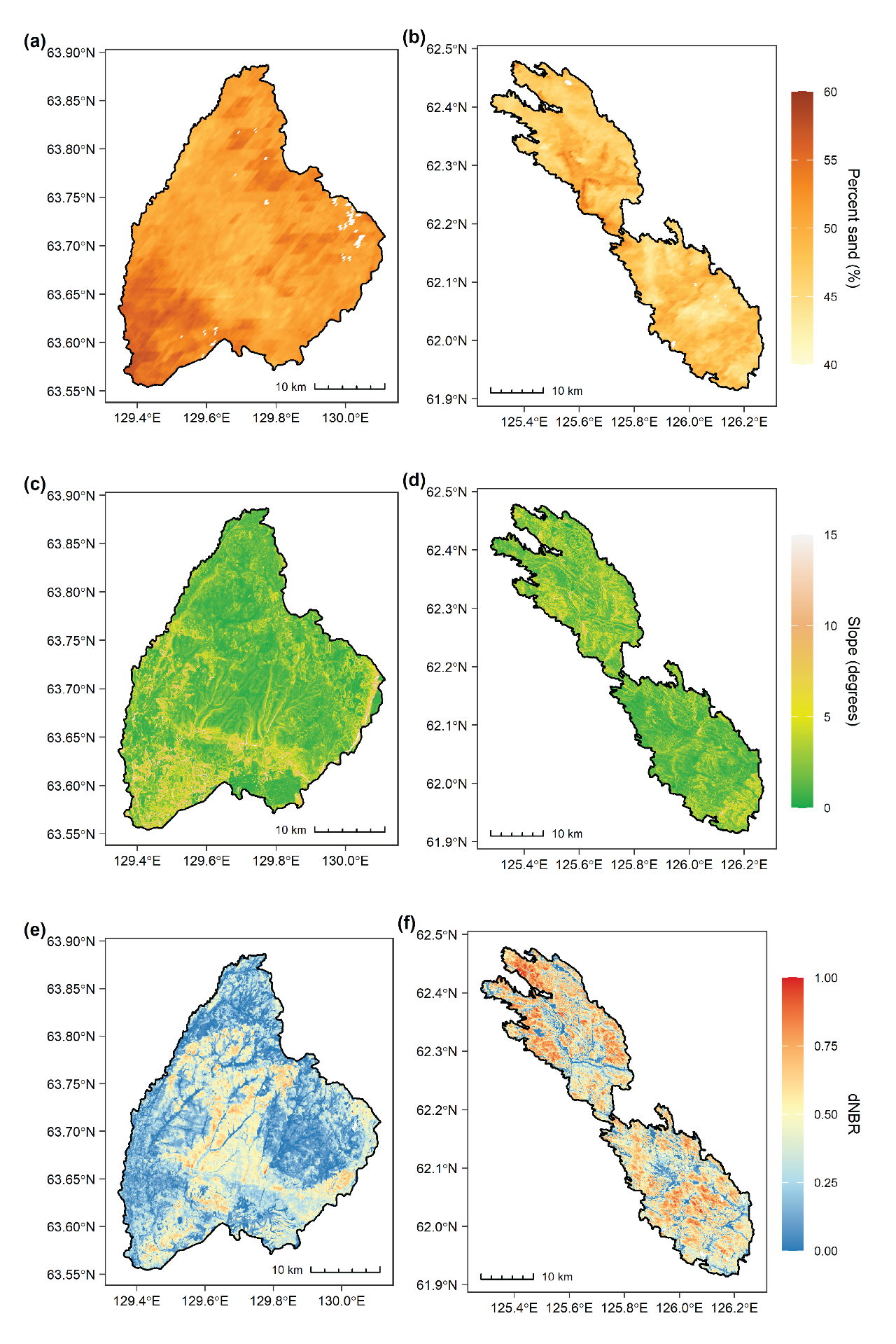


**Figure S7.** Maps of geospatial predictors used in the final model to upscale combustion over Batamay (left) and Yert (right) fire scars. (a,b) percent sand in the top 15 cm of soil (%), (c,d) slope (degrees), and (e,f) differenced Normalized Burn Ratio (dNBR).


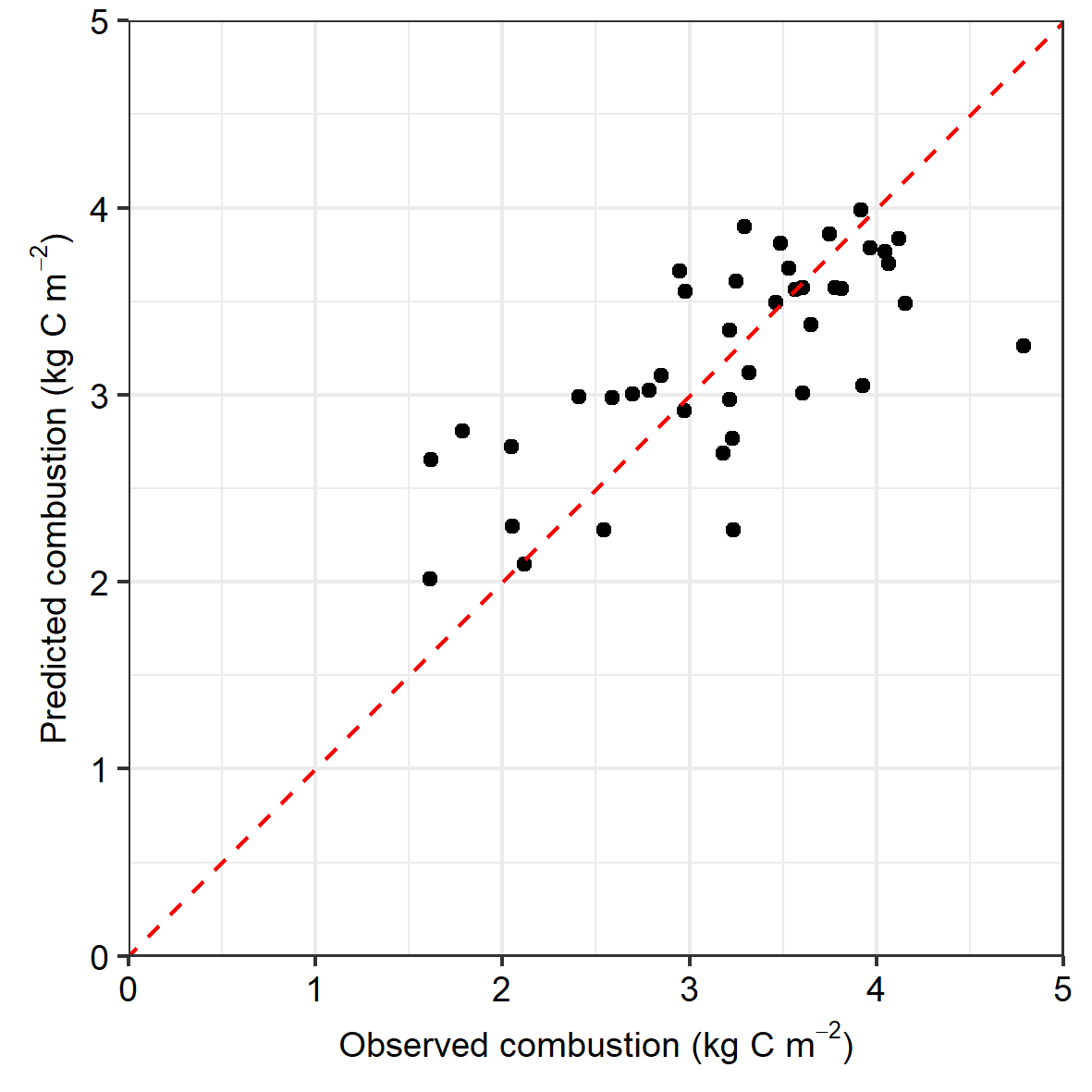


**Figure S8.** Observed and predicted carbon (C) combustion at field plots from a multiple linear regression with inputs of slope, differenced Normalized Burn Ratio, and percent sand in the top 15 cm of soil.

**
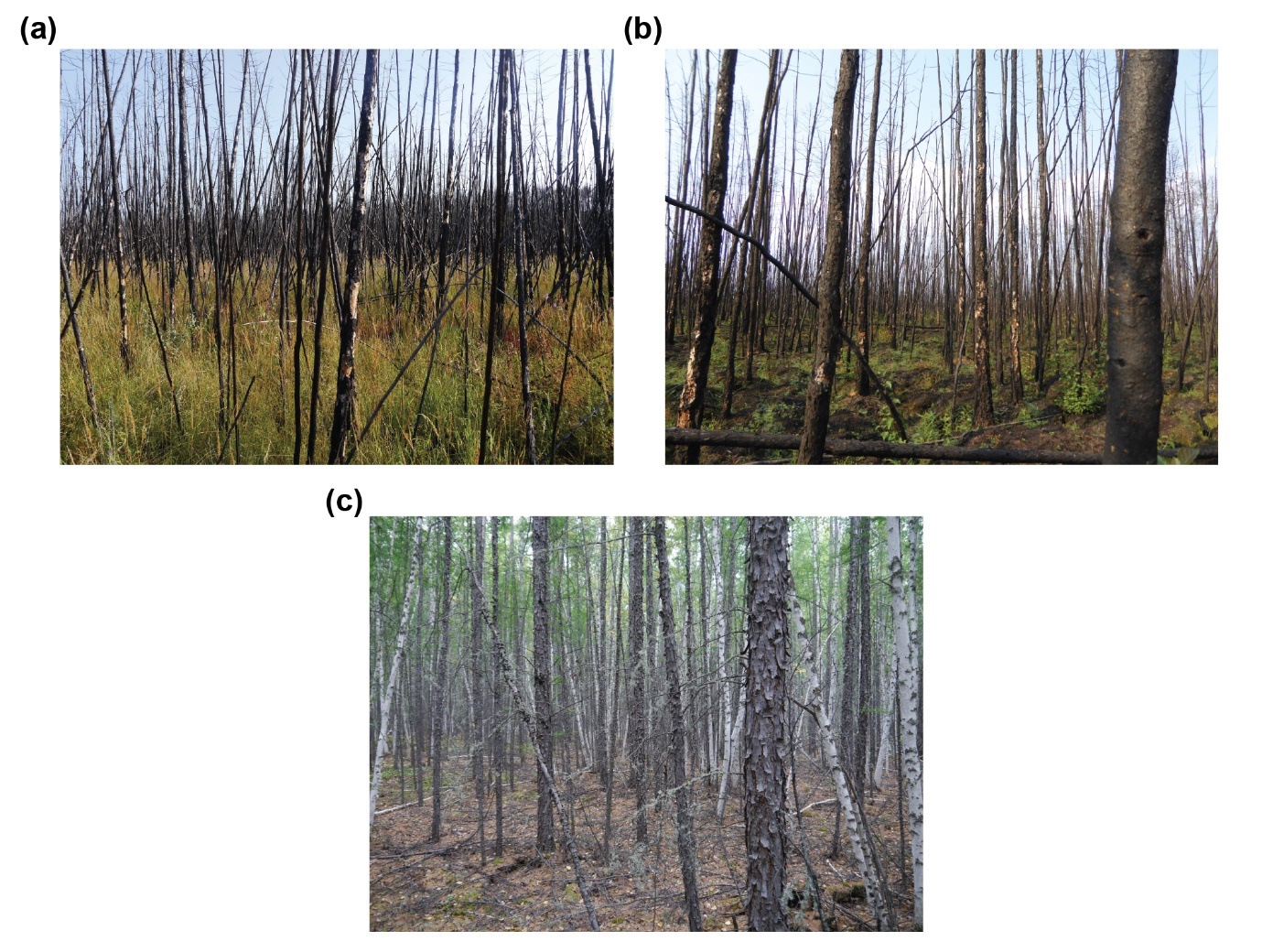
**

**Figure S9.** Field pictures of young and dense larch-dominated stands. High-severity crown fires were observed in (a) Batamay and (b) Yert fire scars. (c) Peeling bark of larch trees.


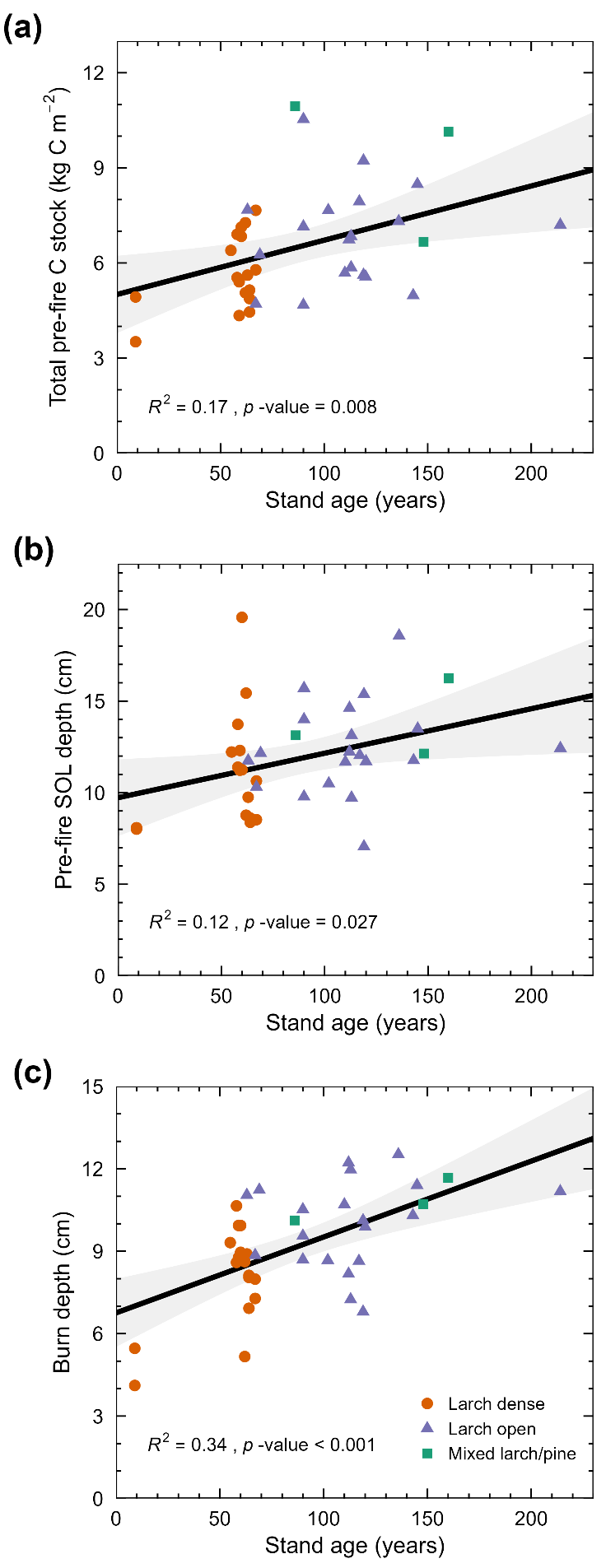


**Figure S10.** Influence of stand age on (a) total pre-fire carbon (C) stock, (b) pre-fire soil organic layer (SOL) depth, and (c) burn depth.


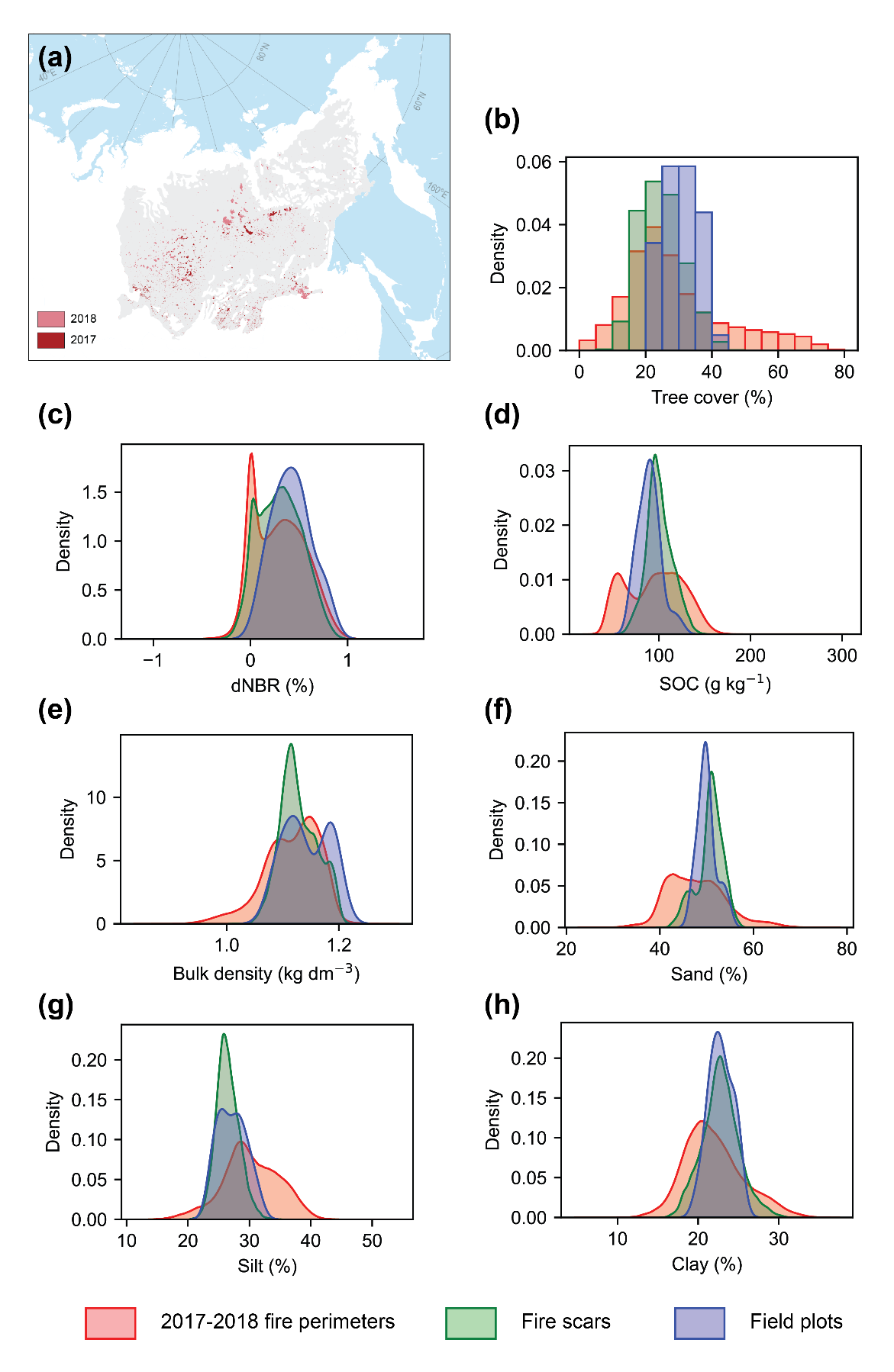


**Figure S11.** Spatial representativeness of field plots. (a) Fire perimeters for 2017 (light red) and 2018 (dark red) (Scholten et al., 2024) in the extratropical forest pyrome 2 (ExTropF2, Jones et al., 2024) cropped to northeastern Siberia (light grey). Histograms (b) and kernel density estimates (KDE) (c–h) show the variability of some of the geospatial variables described in Table S8: tree cover, differenced Normalized Burn Ratio (dNBR), soil organic carbon (SOC), bulk density, and percent sand, silt, and clay in the top 30 cm of soil. Distributions are shown for values extracted at the field plot locations (blue), across the Batamay and Yert fire scars (green), and across the fire perimeters shown in panel (a).

# References

Alexander, H. D., Mack, M. C., Goetz, S., Beck, P. S. A., & Belshe, E. F. (2012). Implications of increased deciduous cover on stand structure and aboveground carbon pools of Alaskan boreal forests. *Ecosphere*, *3*(5), 1−21. <https://doi.org/10.1890/ES11-00364.1>

Alexander, H. D., Mack, M. C., Goetz, S., Loranty, M. M., Beck, P. S. A., Earl, K., Zimov, S., Davydov, S., & Thompson, C. C. (2012). Carbon accumulation patterns during post-fire succession in Cajander larch (*Larix cajanderi*) forests of Siberia. *Ecosystems*, *15*, 1065–1082. <https://doi.org/10.1007/s10021-012-9567-6>

Berner, L. T., Alexander, H. D., Loranty, M. M., Ganzlin, P., Mack, M. C., Davydov, S. P., & Goetz, S. J. (2015). Biomass allometry for alder, dwarf birch, and willow in boreal forest and tundra ecosystems of far northeastern Siberia and north-central Alaska. *Forest Ecology and Management*, *337*, 110–118. <https://doi.org/10.1016/j.foreco.2014.10.027>

Boby, L. A., Schuur, E. A. G., Mack, M. C., Verbyla, D., & Johnstone, J. F. (2010). Quantifying fire severity, carbon, and nitrogen emissions in Alaska’s boreal forest. *Ecological Applications*, *20*(6), 1633–1647. <https://doi.org/10.1890/08-2295.1>

Brown, J. K. (1974). *Handbook for inventorying downed woody material* (General Technical Report INT-16). Ogden, UT, USA: U.S. Department of Agriculture, Forest Service, Intermountain Forest and Range Experimental Station.

Currie, W. S., & Nadelhoffer, K. J. (2002). The imprint of land-use history: patterns of carbon and nitrogen in downed woody debris at the Harvard Forest. *Ecosystems, 5*, 446–460. <https://doi.org/10.1007/s10021-002-1153-x>

Delcourt, C. J. F., Combee, A., Izbicki, B., Mack, M. C., Maximov, T., Petrov, R., Rogers, B. M., Scholten, R. C., Shestakova, T. A., van Wees, D., & Veraverbeke, S. (2021). Evaluating the differenced Normalized Burn Ratio for assessing fire severity using Sentinel-2 imagery in northeast Siberian larch forests. *Remote Sensing*, *13*(12), 2311. <https://doi.org/10.3390/rs13122311>

Delcourt, C. J. F., & Veraverbeke, S. (2022). Allometric equations and wood density parameters for estimating aboveground and woody debris biomass in Cajander larch (*Larix cajanderi*) forests of northeast Siberia. *Biogeosciences*, *19*(18), 4499–4520. <https://doi.org/10.5194/bg-19-4499-2022>

Dieleman, C. M., Rogers, B. M., Potter, S., Veraverbeke, S., Johnstone, J. F., Laflamme, J., Solvik, K., Walker, X. J., Mack, M. C., & Turetsky, M. R. (2020). Wildfire combustion and carbon stocks in the southern Canadian boreal forest: Implications for a warming world. *Global Change Biology*, *26*(11), 6062–6079. <https://doi.org/10.1111/gcb.15158>

Field, R. D., Spessa, A. C., Aziz, N. A., Camia, A., Cantin, A., Carr, R., De Groot, W. J., Dowdy, A. J., Flannigan, M. D., Manomaiphiboon, K., Pappenberger, F., Tanpipat, V., & Wang, X. (2015). Development of a Global Fire Weather Database. *Natural Hazards and Earth System Sciences*, *15*(6), 1407–1423. <https://doi.org/10.5194/nhess-15-1407-2015>

Fogel, R., Ogawa, M., & Trappe, J. M. (1973). *Terrestrial decomposition: a synopsis* (Coniferous Forest Biome Internal Report 135). Seattle, WA, USA: University of Washington.

Hengl, T., Mendes de Jesus, J., Heuvelink, G. B. M., Ruiperez Gonzalez, M., Kilibarda, M., Blagotić, A., Shangguan, W., Wright, M. N., Geng, X., Bauer-Marschallinger, B., Antonio Guevara, M., Vargas, R., MacMillan, R. A., Batjes, N. H., Leenaars, J. G. B., Ribeiro, E., Wheeler, I., Mantel, S., & Kempen, B. (2017). SoilGrids250m: Global gridded soil information based on machine learning. *PLoS ONE*, *12*(2), e0169748. <https://doi.org/10.1371/journal.pone.0169748>

Jones, M. W., Veraverbeke, S., Andela, N., Doerr, S. H., Kolden, C., Mataveli, G., Lucrecia Pettinari, M., Le Quéré, C., Rosan, T. van der Werf, G., van Wees, D., & Abatzoglou, J. T. (2024). Global rise in forest fire emissions linked to climate change in the extratropics. *Science*, *386*, eadl5889. <https://doi.org/10.1126/science.adl5889>

Kajimoto, T., Matsuura, Y., Osawa, A., Abaimov, A. P., Zyryanova, O. A., Isaev, A. P., Yefremov, D. P., Mori, S., & Koike, T. (2006). Size-mass allometry and biomass allocation of two larch species growing on the continuous permafrost region in Siberia. *Forest Ecology and Management*, *222*, 314–325. <https://doi.org/10.1016/j.foreco.2005.10.031>

Krankina, O. N., & Harmon, M. E. (1995). Dynamics of the dead wood carbon pool in northwestern Russian boreal forests. *Water Air Soil Pollution, 82*, 227–238. <https://doi.org/10.1007/BF01182836>

Mäkelä, A., & Vanninen, P. (1998). Impacts of size and competition on tree form and distribution of aboveground biomass in Scots pine. *Canadian Journal of Forest Research*, *28*(2), 216–227. <https://doi.org/10.1139/x97-199>

Manies, K. L., Harden, J. W., Silva, S. R., Briggs, P. H., & Schmid, B. M. (2004). *Soil data from Picea mariana stands near delta junction, Alaska of different ages and soil drainage type* (Open File Report 2004–1271). Anchorage, AK, USA: U.S. Geological Survey, Department of the Interior. <https://doi.org/10.3133/ofr20041271>

Maser, C., Anderson, R. G., Cromack Jr, K., Williams, J. T., & Martin, R. E. (1979). Dead and down woody material. In: J. W. Thomas (Ed), Wildlife Habitats in Managed Forests: the Blue Mountains of Oregon and Washington (Agricultural Handbook No. 553, pp. 78–95). U.S. Department of Agriculture, Forest Service.

McRae, D. J., Alexander, M. E., & Stocks, B. J. (1979). *Measurement and description of fuels and fire behavior on prescribed burns: A handbook* (Information Report O-X-287). Sault Ste. Marie, Ontario, Canada: Great Lakes Forest Research Centre, Canadian Forestry Service.

Nalder, I. A., Wein, R. W., Alexander, M. E., & de Groot, W. J. (1999). Physical properties of dead and downed round-wood fuels in the Boreal forests of western and Northern Canada. *International Journal of Wildland Fire*, *9*(2), 85–99. <https://doi.org/10.1071/WF00008>

Olson, D. M., Dinerstein, E., Wikramanayake, E. D., Burgess, N. D., Powell, G. V. N., Underwood, E. C., D'amico, J. A., Itoua, I., Strand, H. E., Morrison, J. C., Loucks, C. J., Allnutt, T. F., Ricketts, T. H., Kura, Y., Lamoreux, J. F., Wettengel, W. W., Hedao, P., & Kassem, K. R. (2001). Terrestrial Ecoregions of the World: A New Map of Life on Earth: A new global map of terrestrial ecoregions provides an innovative tool for conserving biodiversity. *BioScience*, *51*(11), 933–938. [https://doi.org/10.1641/0006-3568(2001)051[0933:TEOTWA]2.0.CO;2](https://doi.org/10.1641/0006-3568(2001)051%5b0933:TEOTWA%5d2.0.CO;2)

Picard, N., Saint-André, L., & Henry, M. (2012). *Manual for building tree volume and biomass allometric equations*. Rome, Italy: Food and Agricultural Organization of the United Nations, Montpellier, France: Centre de Coopération Internationale en Recherche Agronomique pour le Développement.

Porter, C., Howat, I., Noh, M.-J., Husby, E., Khuvis, S., Danish, E., Tomko, K., Gardiner, J., Negrete, A., Yadav, B., Klassen, J., Kelleher, C., Cloutier, M., Bakker, J., Enos, J., Arnold, G., Bauer, G., & Morin, P. (2023). ArcticDEM - Mosaics, Version 4.1 [dataset]. In *ArcticDEM* (Version V1). Harvard Dataverse. <https://doi.org/doi:10.7910/DVN/3VDC4W>

Schepaschenko, D., Shvidenko, A., Usoltsev, V., Lakyda, P., Luo, Y., Vasylyshyn, R., Lakyda, I., Myklush, Y., See, L., McCallum, I., Fritz, S., Kraxner, F., & Obersteiner, M. (2017). A dataset of forest biomass structure for Eurasia. *Scientific Data*, *4,* 170070. <https://doi.org/10.1038/sdata.2017.70>

Scholten, R. C., Chen, Y., Veraverbeke, S., & Randerson, J. T. (2024). Arctic–boreal fire atlas: 12-hourly perimeters of individual fires in the Arctic–boreal domain from 2012 to 2023. Pangaea. <https://doi.org/10.1594/PANGAEA.967653>

Schroeder, W., Oliva, P., Giglio, L., & Csiszar, I. A. (2014). The New VIIRS 375m active fire detection data product: Algorithm description and initial assessment. *Remote Sensing of Environment*, *143*, 85–96. <https://doi.org/10.1016/j.rse.2013.12.008>

Sexton, J. O., Song, X. P., Feng, M., Noojipady, P., Anand, A., Huang, C., Kim, D.-H., Collins, K. M., Channan, S., DiMiceli, C., & Townshend, J. R. (2013). Global, 30-m resolution continuous fields of tree cover: Landsat-based rescaling of MODIS vegetation continuous fields with lidar-based estimates of error. *International Journal of Digital Earth*, *6*(5), 427–448. <https://doi.org/10.1080/17538947.2013.786146>

Smith, A., Granhus, A., Astrup, R., Bollandsås, O. M., & Petersson, H. (2014). Functions for estimating aboveground biomass of birch in Norway. *Scandinavian Journal of Forest Research*, *29*(6), 565–578. <https://doi.org/10.1080/02827581.2014.951389>

Ter-Mikaelian, M. T., & Korzukhin, M. D. (1997). Biomass equations for sixty-five North American tree species. *Forest Ecology and Management*, *97*(1), 1–24. <https://doi.org/10.1016/S0378-1127(97)00019-4>

Van Wagner, C. E. (1982). *Practical aspects of the line intersect method* (Information Report PI-X-12E). Fredericton, New Brunswick, Canada: Canadian Forestry Service, Maritimes Forest Research Centre.

Wirth, C., Schulze, E. D., Von Stünzner-Karbe, D., Ziegler, W., Miljukova, I. M., Sogatchev, A., Varlagin, A. B., Panvyorov, M., Grigoriev, S., Kusnetzova, W., Siry, M., Hardes, G., Zimmermann, R., & Vygodskaya, N. N. (1999). Above-ground biomass and structure of pristine Siberian Scots pine forests as controlled by competition and fire. *Oecologia*, *121*, 66–80. <https://doi.org/10.1007/s004420050908>

Yang, Y. Z., Cai, W. H., Yang, J., White, M., & Lhotka, J. M. (2018). Dynamics of postfire aboveground carbon in a chronosequence of Chinese boreal larch forests. *Journal of Geophysical Research: Biogeosciences*, *123*(12), 3490–3506. <https://doi.org/10.1029/2018JG004702>
